# Most sleep does not serve a vital function: Evidence from *Drosophila melanogaster*

**DOI:** 10.1126/sciadv.aau9253

**Published:** 2019-02-20

**Authors:** Quentin Geissmann, Esteban J. Beckwith, Giorgio F. Gilestro

**Affiliations:** Department of Life Sciences, Imperial College London, London, UK.

## Abstract

Sleep appears to be a universally conserved phenomenon among the animal kingdom, but whether this notable evolutionary conservation underlies a basic vital function is still an open question. Using a machine learning–based video-tracking technology, we conducted a detailed high-throughput analysis of sleep in the fruit fly *Drosophila melanogaster*, coupled with a lifelong chronic and specific sleep restriction. Our results show that some wild-type flies are virtually sleepless in baseline conditions and that complete, forced sleep restriction is not necessarily a lethal treatment in wild-type *D. melanogaster*. We also show that circadian drive, and not homeostatic regulation, is the main contributor to sleep pressure in flies. These results offer a new perspective on the biological role of sleep in *Drosophila* and, potentially, in other species.

## Teaser

Sleepless fruit flies live, an unexpected finding that provides a new perspective on sleep for this and perhaps other species.

## INTRODUCTION

It is widely speculated that sleep serves a fundamental biological need, an idea derived from three distinct lines of evidence: (i) Sleep is a universally conserved phenomenon across evolution, (ii) chronic sleep restriction is often associated to death, and (iii) a sleepless animal has never been found [all reviewed in (*1*–*4*)].

The first of these three aspects—the striking evolutionary conservation of sleep—constitutes an important conundrum for scientists, but alone cannot be taken as proof that sleep plays a vital function. Circadian rhythms, for instance, are also universally conserved, ultimately providing a clear evolutionary advantage, but they are not intrinsically vital to the individual given that animals can survive without a functional internal clock (*5*, *6*).

The fundamental question therefore is: “Can an animal survive without sleep?”

The study of chronic sleep deprivation could, at least in principle, address this challenge. Unfortunately, the literature on the chronic effects of sleep restriction is not comprehensive, partly dated, and intrinsically complicated by the many confounding factors that correlate with sleep restriction. To date, experiments addressing this question have been reported in a handful of species only: dogs [reviewed in (*7*)], rats [reviewed in (*8*)], cockroaches (*9*), pigeons (*10*), and fruit flies (*11*). In four of the five tested animal species, sleep deprivation experiments eventually terminated with the premature death of the animals, but the underlying cause of lethality still remains unknown. In rats and dog pups, death is associated with a severe systemic syndrome bearing important metabolic changes and clear signs of suffering, making it difficult to ultimately conclude whether lethality is caused by the mere removal of sleep or rather by the very invasive and stressful procedures used to keep the animals awake (*7*, *12*, *13*). In the cockroach *Diploptera punctata*, sleep deprivation was achieved by continuously startling the animals (*9*), without, however, accounting for exhaustion-induced stress, a known lethal factor for other species of cockroaches (*14*–*16*). The observations in *Drosophila* are limited in terms of throughput and methodology (*11*). In pigeons, chronic sleep deprivation was shown not to be lethal (*10*). In conclusion, chronic sleep deprivation experiments appear suggestive, but inconclusive, for multiple reasons.

The third line of evidence supporting the hypothesis that sleep serves a fundamental biological need is perhaps the strongest and concerns the fact that sleepless individuals could never be identified, neither in nature nor through artificial laboratory screenings. We know that some species, such as elephants (*17*) or giraffes (*18*), have evolved to cope with limited amount of sleep and several genetic mutations, conferring that short-sleeping phenotypes in flies, rodents, and humans have been characterized in the past two decades [reviewed in (*19*)]; some animals are also able to forego sleep for days or weeks in particular ecological conditions (*17*, *20*–*23*), but the identification of a constantly sleepless animal can be considered a holy grail of the field.

Given that we still ignore what sleep does at the cell biological level, in all animals sleep quantification relies exclusively on bona fide macroscopic correlates, either electrophysiological or behavioral. Therefore, a technological development able to improve the characterization of these correlates may provide a more accurate description of sleep, laying the conditions for a more specific sleep deprivation procedure. To this end, we recently created a system that allows a faithful high-throughput analysis and manipulation of *Drosophila* sleep using activity as its behavioral correlate [ethoscopes (*24*)]. Here, we report two surprising findings that were uncovered using this system, challenging the notion that sleep is a vital necessity: the discovery of virtually sleepless flies and the finding that chronic sleep restriction in *Drosophila melanogaster* has notably less pronounced effects on longevity than previously thought.

## RESULTS

### Virtually sleepless flies are found in a nonmutant population

**Fig. 1:**
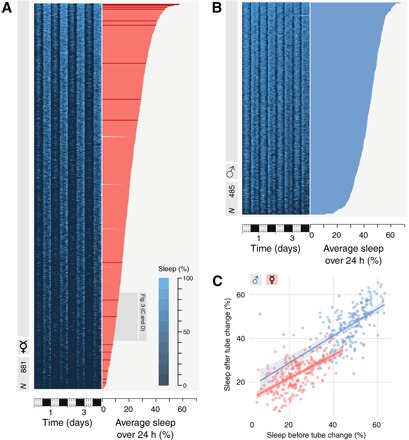
Great variability in sleep amounts in a nonmutant population of *D. melanogaster*. Descending sorted distribution of sleep amount (**A**) in a group of 881 female CantonS flies and (**B**) in a group of 485 male CantonS flies. In both panels, the left graph shows sleep amount for each individual fly over a period of 5 days in bouts of 30 min [legend in (A)]. The right graph indicates the average sleep amount in 24 hours for female [pink in (A) and the rest of the figures] and male [cyan in (B) and the rest of the figures] flies. The 19 female animals whose sleep is highlighted in red are the ones for which raw video sample is available at the ZENODO repository (*37*). (**C**) Average sleep amount measured in a tube predicts sleep amount measured in a different tube. Average of 6 days for both, with 1 day in between (*n*_male_ = 242 and *n*_female_ = 242).

Prolonged periods of inactivity are an evolutionarily conserved, experimentally convenient behavioral correlate of sleep (*25*). Absence of movement is therefore routinely used as a proxy to measure sleep across a wide range of animals, spanning from jellyfish to elephants (*17*, *26*, *27*). In *Drosophila* too, sleep can be estimated by measuring the absence of walking bouts, generally using a commercially available device to detect whenever an isolated fly crosses the midline of a tube (*28*). This system, however, provides only limited spatial resolution that—unsurprisingly—results in an overestimation of sleep amounts (*29*). A growing number of laboratories are therefore transitioning to more accurate systems based on computer-assisted video tracking (*29*–*33*). To further improve our confidence in sleep estimation, we recently introduced a machine learning approach that uses supervised learning to detect not only walking activity but also micromovements (for example, in-place movements such as grooming, egg laying, and feeding) (*24*). How much do flies really sleep when, besides their walking activity, we measure their micromovements too? To answer this question, we analyzed sleep for four consecutive days in 881 female (Fig. 1A) and 485 male (Fig. 1B) CantonS flies, a commonly used laboratory “wild-type” strain. As expected, in both males and females, sleep amounts were widely distributed, with male flies sleeping for 618.5 (CI_95%_, 606.7 to 630.3) min a day and female flies sleeping for 299.2 (CI_95%_, 288.8 to 309.6) min a day [mean (95% bootstrap confidence interval)]. The distribution of sleep amount in females uncovered a previously undescribed fraction of extreme short sleepers: 50% of female flies slept less than 20% of their time and 6% slept for less than 5% of their time (72 min a day). At the very end of the curve laid three flies that spontaneously slept an average of 15, 14, and 4 min a day, respectively (Fig. 1A and fig. S1). In both males and females, sleep amount is an endogenous feature: When flies are transferred into a novel environment (i.e., a fresh tube in a novel ethoscope inside a different incubator), their sleep amount remains mostly similar to their past sleep (*R*^2^ = 0.77; CI_95%_, 0.73 to 0.81; Fig. 1C).

### Micromovements explain the short-sleeping phenotype

**Fig. 2:**
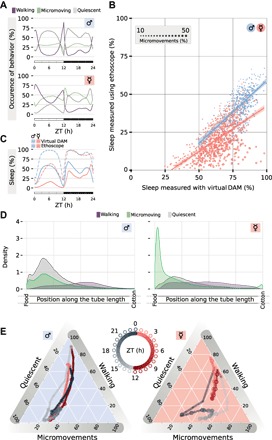
Micromovements account for the newly described short-sleeping phenotype. (**A**) Average occurrence of behavior over the 24-hour period in male (top) and female (bottom) CantonS flies. (**B**) Sleep amount for each individual male (cyan) and female (pink) fly plotted as computed with ethoscopes (*y* axis) and with virtual Drosophila Activity Monitor (vDAM) analysis (*x* axis) (*31*). The size of each dot represents the average amount of micromovements observed over the 24-hour period. (**C**) Average sleep amount over the 24-h period in male and female flies, plotted as computed with ethoscopes (continuous lines) or virtual DAM analysis (dashed lines). (**D**) Average positional distribution of behaviors for male (left) and female (right) flies over the 24-hour period, broken into the three behavioral states identified by ethoscopes. (**E**) Four-dimensional representation of behavioral transitions over the 24-hour period. Gray shades indicate the dark period (ZT12 to ZT24), while red shades indicate the light period (ZT0 to ZT12). Same dataset shown in Fig. 1 (A and B).

Short-sleeping flies have been identified in the past, either through experimental selection (*34*, *35*) or through selected mutagenesis (*36*), but flies (and, in fact, animals) sleeping as little as few minutes a day were never identified before. To confirm the validity of our results, we reviewed the positional tracings of all 881 female flies in the dataset (fig. S1) and acquired and reviewed videos for 19 flies with representative sleep amounts ranging from 823 to 42 min a day to compare the tracking record at the single fly level [dark red lines in Fig. 1A; raw whole videos are available at (*37*)]. Manual inspection (fig. S1) and quantitative analysis (Fig. 2) confirmed that the activity repertoire oscillates in a stereotyped, sexually dimorphic manner (Fig. 2A), with micromovements being mostly present in females [Fig. 2B—in females, 623.4 (CI_95%_, 615.3 to 631.4) min a day had at least one micromovement episode, while in males 411.4 (CI_95%_, 404.2 to 418.8) min did]. As expected, micromovements (Fig. 2B) and movements that do not span the entire tube length (fig. S1) are responsible for the quantitative difference in sleep analysis between recording platforms (Fig. 2C). Female micromovements and quiescence are spatially (Fig. 2D) and, to a certain extent, temporally (Fig. 2A) exclusive: 37.3% (CI_95%_, 36.9 to 37.7) of the micromovements happen at night (ZT12 to ZT21), of which 51.3% (CI_95%_, 50.3 to 52.2) within 4 mm from the food (Fig. 2, A and D, respectively, green). In short, expanding on previously reported findings (*38*, *39*), micromovements in females are concentrated to those times of the day when flies are known to increase feeding activity (i.e., during mid-day and in the early phase of the night), mostly located by the food and away from the preferred site for quiescence (movie S1 and Fig. 2), suggesting that the micromovements observed in female flies are not a sleeping-related behavior but a feeding-related behavior.

### Qualitatively different types of short-sleeping females

**Fig. 3:**
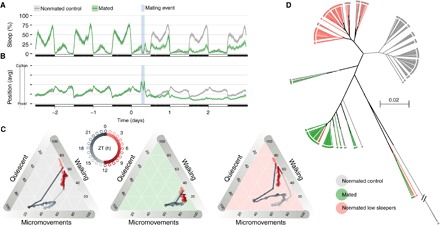
Mating reduces sleep amount. (**A**) Sleep profile of all the female flies used in the mating experiment: green, flies that underwent successful mating event (*n* = 86); gray, flies that met a male but did not engage in copulation (*n* = 152). The light blue vertical shade indicates the timing of the mating event. (**B**) Average position along the tube of the same flies shown in (A) in 30-min bins. (**C**) Four-dimensional representation of behavioral transitions over the 24-hour period for nonmated flies (gray background), mated flies (green background), and naturally short-sleeping unmated flies (pink background; same dataset highlighted in gray in Fig. 1A). (**D**) Hierarchical clustering based on pairwise distance, in the time-behavior domain, of the same three cohorts shown in (C).

High-throughput ethoscope analysis allowed us to identify wild-type female flies that sleep as little as few minutes a day (Fig. 1A and fig. S1). Could this be a peculiarity of some virgin flies, hence an ethological laboratory artefact? In *Drosophila*, mating status is known to be acting as a major behavioral switch (*40*) that modifies, among other behaviors, the animals’ dietary preference (*38*, *41*) and their preference for feeding time (*42*). However, reaching sexual maturity only few hours after ecdysis, virgin female flies are likely to be a rare occurrence in the wild (*43*, *44*). To test how sleep changes with mating status, we recorded sleep in female flies before and after a successful (green) or unsuccessful (gray) mating event (Fig. 3A). In newly mated females, sleep dramatically decreased for at least three consecutive days [Fig. 3A—in the first full day after mating, lowering from 383.2 (CI_95%_, 363.4 to 404.9) to 175.3 (CI_95%_, 153.0 to 200.2) min a day], correlating with a major change in the positional preference of the animals toward the food (Fig. 3B). This change in positional preference (Fig. 3B) and the strong increase in micromovements (Fig. 3C) are likely to represent an increase in food intake and egg-laying activity and may explain why such a strong decline in sleep amount was never identified using different tools (*29*, *45*, *46*). Four-dimensional behavioral fingerprinting showed that the short-sleeping phenotype observed upon mating is qualitatively different from the one observed as natural variation in the CantonS population (Fig. 3, C and D, and fig. S2).

### Prolonged sleep deprivation has little or any lethal consequences

**Fig. 4:**
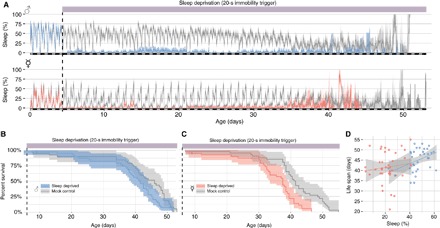
Chronic mechanical sleep deprivation is largely not lethal in *D. melanogaster*. (**A**) Lifelong sleep restriction in male (top) or female (bottom) CantonS flies subjected to mechanical sleep deprivation triggered by a 20-s inactivity bout. (**B**) Survival curve for male (cyan) or (**C**) female (pink) sleep-deprived flies and their sex-matched undisturbed mock control [gray in both (B) and (C)]. Sleep measurements become noisier as the number of flies decreases. *n* = 38 to 40 for all four groups. (**D**) Linear regression analysis in search of a correlation between sleep amount (*x* axis) and life span (*y* axis) in individual undisturbed female (pink) and male (cyan) flies. Same dataset as the gray flies in (A) and (B).

The experiments described so far unveiled that a fraction of female flies necessitate little sleep, with some being almost completely sleepless. Is sleeplessness a peculiarity of a few special individuals, or can any fruit fly cope with little or no sleep? To answer this question, we conducted a lifelong sleep deprivation experiment using a closed-loop sleep deprivation device able to interact with single animals by triggering a tube rotation after a predefined period of immobility (*24*), a system created to minimize the extent of disturbance and conceptually inspired by the disc-over-water apparatus developed by the Rechtschaffen laboratory (*8*), in which a rat receives a waking physical challenge only when it is factually asleep but is left undisturbed otherwise. In our setup, flies were housed in individual tubes and each tube experienced a 1-s rotation at the approximate speed of 300 rpm whenever the animal housed inside had shown 20 s of continuous immobility (*24*). The treatment led to a highly efficient sleep deprivation, with flies losing, on average, 95.6% (CI_95%_, 93.5 to 98.2) of their sleep (Fig. 4A), and yet, surprisingly, we could not detect any major effect on survival (Fig. 4, B and C). In particular, sleep-deprived male flies lived as long as the control group [with a median of 41.5 (CI_95%_, 38.0 to 44.0) days against 46.0 (CI_95%_, 41.0 to 48.5) days for the controls], and a statistically relevant effect was only evident in female flies, with a reduction of median life span of 3.5 days [37.5 (CI_95%_, 33.0 to 38.5) and 41.0 (CI_95%_, 38.5 to 44.0)]. In flies, forced sleep restriction has little or no consequences on life span when performed in a controlled, specific manner. Given the variability of sleep within our wild-type population (Fig. 1, A and B), we also wondered whether sleep amount in an individual fly could predict its life span. We performed a linear regression (see Materials and Methods) and found no overall effect, neither in males [+1.05 days of life per hour of sleep a day (CI_95%_, −0.08 to 1.91); Fig. 4D, cyan] nor in females [+0.41 days of life per hour of sleep a day (CI_95%_, −0.51 to 1.55); Fig. 4D, pink] (overall *R*^2^ = 0.12). That is, as previously suggested (*34*), we confirm here that spontaneous short sleepers do not die faster and, conversely, high sleepers do not tend to live longer.

### Sleep rebound after sleep deprivation only partly correlates with sleep loss

**Fig. 5:**
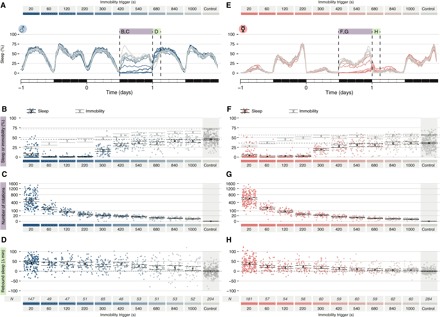
Sleep rebound is not linearly proportional to sleep loss. (**A** and **E**) Sleep profile for the entire dataset: 818 male (A to **D**) and 912 female (E to **H**) CantonS flies. (B and F) Sleep (cyan and pink dots and black markers) or immobility (gray markers) for the entire dataset spanning 10 different immobility interval triggers (20 to 1000 s). Control flies were never actively stimulated but laid adjacently to the experimental flies. (C and G) Number of tube rotations triggered by immobility bouts. (D and H) Amount of rebound sleep in the ZT0 to ZT3 interval following the sleep deprivation for the entire dataset.

If most (all?) sleep does not serve a direct and immediate vital function, do we need to rethink the current prevailing concept of sleep homeostasis? Is sleep rebound truly a way to make up for a loss of an otherwise impaired biological process, or is it—totally or in part—a phenomenon evolved to guarantee that a constant, largely species-specific amount of sleep is met (*47*, *48*)? To explore this dichotomy, we analyzed how different treatments of sleep deprivation would affect sleep rebound. To start, we conducted an acute sleep deprivation experiment on a total of 818 male (Fig. 5, A to D) and 992 female (Fig. 5, E to H) CantonS flies, with a comprehensive range of immobility triggers spanning from 20 to 1000 s, to deprive flies of sleep episodes of specific length. As expected, the total amount of sleep lost during the 12 hours of deprivation positively correlated with the length of the immobility trigger adopted (Fig. 5, B and F), while the number of stimuli delivered was inversely correlated (Fig. 5, C and G). In all cases, we could observe a statistically significant sleep rebound in the first 3 hours following the sleep deprivation, also when the sleep loss was not statistically different from control (840- and 1000-s inactivity triggers; Fig. 5F). In particular, depriving female flies of only the longest sleep episodes (≥1000 s) still led to a significant sleep rebound the subsequent morning (i.e., the first 3 hours after sleep deprivation), although flies experienced, on average, only 5.8 (CI_95%_, 4.8 to 6.8) tube rotations per night.

**Fig. 6:**
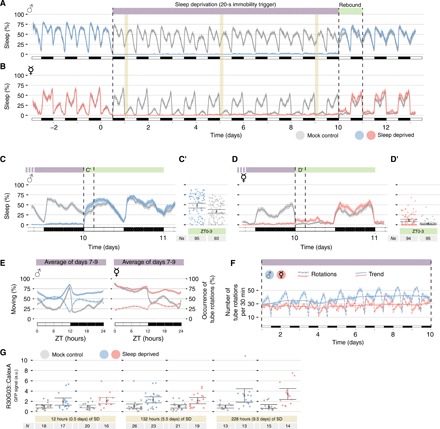
Sleep pressure is largely under the control of the circadian rhythm. (**A** and **B**) Sleep profile for male (A, cyan) and female (B, pink) CantonS flies during the length of the experiment compared with their sex-matched undisturbed mock controls (gray in both). Day 0 marks the beginning of the chronic sleep deprivation procedure, lasting 228 hours (indicated by a purple shade on top). The green shade indicates the rebound day blown up in (C) and (D). The three yellow shades mark the timings chosen for the CaLexA quantifications shown in (G). (**C** and **D**) Magnification of sleep deprivation to rebound transition. (C′ and D′) Quantification of sleep amount during ZT0 to ZT3 of rebound day. (**E**) Moving activity of flies (continuous lines) and number of rotations over the average 24-hour period (dashed lines). Moving activity combines both walking and micromoving. (**F**) Average number of tube rotations over the length of the sleep deprivation experiment (dashed lines) or seasonal trend (continuous lines; see Materials and Methods for details). *n* = 93 to 95 for all four groups. (**G**) Quantification of CaLexA-dependent green fluorescent protein (GFP) levels in the subregion of the ellipsoid bodies labeled by the expression of the R30G03 driver after 0.5, 5.5, and 9.5 days. *Ns* are indicated in the panel. a.u., arbitrary units.

The data shown so far indicate that the increase in sleep pressure that drives rebound after sleep deprivation is not linearly correlated with the amount of sleep lost over the length of one night, but how do flies react to prolonged sleep restriction spanning multiple days? To answer this question, we conducted a “Randy Gardner”–like experiment (*49*), in which we subjected flies to 228 hours (9.5 days) of uninterrupted sleep deprivation, using a 20-s immobility trigger as waking event (Fig. 6). The experiment was conducted in both male and female flies, using mock control individuals in adjacent tubes, for a total of 377 animals. Even after almost 10 days of chronic sleep deprivation, male flies manifested a sleep rebound that was not dissimilar from the rebound observed after one night of acute sleep restriction (compare Fig. 6C to Fig. 5A). While in male flies rebound sleep was again limited to the first 3 hours of the rebound day (Fig. 6, A and C), in female flies the observed sleep rebound was quantitatively modest but protracted in time for the subsequent 3 days at least (Fig. 6, B and D). As expected, walking activity in these animals was increased by the persistent stimulation, with a stark increase in walked distance at the beginning of the treatment slowly decreasing with time, possibly due to fatigue (fig. S3). Because the tube rotations were triggered by immobility, we could use the number of rotations (Fig. 6E, dashed lines) as proxy for endogenous sleep pressure sleep-deprived (pink and cyan) flies. In both male and female flies, the main changes in sleep pressure were cycling in a circadian fashion, with the clock-regulated bouts of activity still showing no sign of subsidence, despite the long sleep deprivation (Fig. 6E, solid lines). That is, even after days of continuous sleep deprivation, flies were spontaneously very active at dusk and dawn and hardly any stimulation was needed at those times to keep them awake (Fig. 6E), suggesting that when the circadian clock commands activity, the flies are active also after days and days of cumulated sleep pressure. Analyzing the seasonal decomposition of rotations over the 9.5 days of sleep deprivation, we concluded that only a small amount of the variance in sleep pressure could be explained by the long-range trend in sleep deprivation (21% in males and 11% in females; Fig. 6F), while the main contributor of sleep pressure was instead circadian periodicity (69% in males and 61% in females; Fig. 6F, continuous lines). To analyze changes in the molecular correlates of sleep pressure, we used the CaLexA system (*50*) to gauge the neuronal activity of the R2 neurons of the ellipsoid body after 0.5, 5.5, and 9.5 days of chronic sleep deprivation (Fig. 6G). At all three time points, we observed an increase in bona fide R2 activity in both male and female flies to an extent similar to what was previously reported (*20*, *51*), with sleep-deprived flies showing a two- to fourfold increase when compared to their age-matched controls (Fig. 6G). To further confirm the role of a functioning circadian clock in regulating sleep pressure during prolonged sleep deprivation, we subjected *Clk*^*Jrk*^ mutant flies to prolonged sleep deprivation (fig. S4). Overall sleep pressure, as measured through the number of induced tube rotations (fig. S4, C and D), was decreased in *Clk* mutant animals, and importantly, complete removal of any environmental zeitgeber obtained rearing *Clk*^*Jrk*^ flies in a constant dark environment resulted in no oscillation in the number of tube rotations along the day (fig. S4D). These data, taken together, indicate that the main stimulus to rest in flies is driven by the circadian clock.

## DISCUSSION

The idea that sleep fulfills a vital biological need—we initially argued—relies on one fundamental question: Can we find an animal able to survive without sleep? According to the data presented here, the answer could be “yes.” In wild-type *D. melanogaster*, the need for sleep is not a vital necessity and lack of sleep—either endogenously driven (Fig. 1) or artificially imposed (Figs. 4 and 6)—is compatible with life. The utmost conceptual importance of these findings commands caution, and caveats must be critically examined.

First, it is important to converge on the definition of sleep as a nonvital necessity. In all animals studied so far, sleep deprivation is associated with severe physiological and cognitive decline. It remains plausible that, in a less “forgiving” environment in which flies experience predation and competition, sleep deprivation could end up being a lethal treatment in the same way sleep deprivation may become a lethal treatment to a human conducting a motor vehicle. Using an analogy, a wingless fly would certainly have normal life span in the laboratory but probably not in the wild. On this line, one could argue that sleep is an evolutionary, but not physiologically, vital phenomenon.

Second, we cannot rule out that, in our sleep deprivation experiments, flies still experience enough sleep to satisfy a hypothetical vital need. That is, prolonged or consolidated sleep is not a vital necessity but micro-intervals of sleep that last only few seconds at the time may be sufficient to satisfy whatever basic biological need that sleep may serve. The concept that micro-episodes of sleep account for whichever vital biological function comes with the caveat that microsleep reductionism asymptotically converges toward a tautology.

Our results uncover an interesting sexual dimorphism in terms of natural sleep need and in terms of response to sleep deprivation: While female flies are able to cope with much less sleep in baseline conditions, they are more sensitive to sleep deprivation, with an extended rebound upon long sleep restriction (Fig. 6, B and D) and a moderate but significant effect on lethality upon lifelong sleep deprivation (Fig. 4). This sexual dichotomy may be instrumental in the future to dissect the difference between different sleep functions, confirming that flies are an excellent model to study the function of sleep, not just its correlates. One interesting aspect of this dimorphism that remains to be explored is the source of variability in sleep amount. Given that individual flies appear to retain an unchanged sleep profile under different environmental conditions (Fig. 1C), it is safe to assume that this variability is somehow internally driven, for instance, through uncontrolled genetic variability or stochastic developmental variability. Previous studies on isogenic lines reached similar conclusions (*34*, *35*), and the topic remains to be explored.

At first sight, the results presented here appear to be clashing with some of the existing knowledge. In our view, they command, instead, for a thorough review of existing sleep deprivation literature. The experiments of chronic sleep deprivation performed in dog pups at the end of the 1800s are universally considered too primitive to be trustworthy and too unethically stressful to be reproducible in modern times (*7*). The early *Drosophila* experiments were too preliminary to depict a whole picture (*11*). Other lines of research have also shown no correlation between sleep loss and survival in flies: Loss of the *insomniac* (*52*) or *fumin* (*36*) genes leads to strong sleep restriction that is still compatible with life. Likewise, artificially selected short-sleeping fruit flies have unaltered longevity (*34*). With flies joining pigeons in the list of animals surviving chronic sleep deprivation, the only solid evidence in favor of lethality upon sleep deprivation lies with the chronic sleep deprivation in rats using the disc-over-water system. Those experiments, however, were not free of confounding factors, and one cannot exclude a stress or metabolic component, given that animals were thrown into water up to hundreds of times a day (*13*). In humans, for obvious ethical reasons, we have no experimental evidence that prolonged sleep deprivation is incompatible with life. A human prion disease, fatal familial insomnia (FFI), is sometimes brought as evidence of a vital function of sleep, yet bearing too many confounding factors, considering the devastating nature of the pathology (*53*). Transmitted (*54*) and transgenic mouse models (*55*) of FFI reproduce clear signs of neurodegeneration and premature death, but not sleeplessness, suggesting that, in humans, insomnia is a symptom of the disease but not necessarily the cause of death (*56*). In conclusion, we believe that our results imply that sleep does not serve a unique, evolutionarily conserved function, but it is rather the combination of different biological and evolutionary drives. At the same time, they confirm that *Drosophila* is an excellent model to attempt a transition from the study of sleep correlates to the study of sleep functions.

## MATERIALS AND METHODS

### Fly stocks and rearing conditions

Flies were raised under a 12-hour light/12-hour dark (LD) regimen at 25°C on standard corn and yeast media. CantonS flies from R. Stanewsky (University of Münster, Germany) were used as wild-type flies, and *Clk*^Jr.k^ flies (Bloomington Drosophila Stock Center #24515) (*57*) were used as a clock mutant. To estimate the activity on the R2 neurons of the ellipsoid body, we used the R30G03-GAL4 (Bloomington Drosophila Stock Center #49646) (*51*) strain in combination with CaLexA (*50*). All analyzed animals were socially naïve, unless otherwise stated.

### Neuronal activity in the R2 neurons of the ellipsoid body: CaLexA measurements

Animals were grown and treated in the same conditions as in behavioral experiments. After a sleep deprivation of 0.5, 5.5, or 9.5 days, animals were anesthetized on ice, and brains were dissected in phosphate buffer and fixed with 4% paraformaldehyde, as previously described (*58*). For the quantification of green fluorescent protein (GFP), fly brains were labeled with anti-GFP (1:300; ab290, Abcam). Images were taken under ×400 magnification using a Leica SP8 inverted scanning confocal microscope in the Facility for Imaging by Light Microscopy (FILM), Imperial College London. Data were analyzed using Fiji/ImageJ (*59*). The measurement of signal intensities was performed as previously described (*20*).

### Behavioral experiments

For all experiments, 7- to 8-day-old pupae were sorted into glass tubes [70 mm × 5 mm × 3 mm (length × external diameter × internal diameter)] containing the same food used for rearing. After eclosion, animals were sorted according to their sex, and then the tubes were loaded into ethoscope “sleep arenas” (20 animals per device) (*24*). Three days of baseline were recorded before any treatment. All experiments were carried out under LD conditions (50 to 70% humidity) in incubators set at 25°C and with ad libitum access to regular food. Animals that died during the experiment were excluded from the analysis, except for the longevity experiments.

To evaluate the effect of mating on sleep (Fig. 3), a naïve male was introduced in the tube of each naive female and allowed to interact for 2 hours from ZT06 to ZT08 (zeitgeber time). After the interaction, males were removed and the activity profile of the females was recorded for another 3.5 days. The short duration of the interaction and the restrictive space of the glass tube reduce the probability of mating, and only about 50% of the flies underwent successful mating. This setup provides the two necessary groups: mated females and females that were courted but not mated. Effective mating was scored as the presence of larvae in the food 4 days after the interaction.

The “rotational module” of the ethoscope platform was used to perform the 12-hour dynamic sleep deprivation treatments shown in Fig. 5. Different durations of immobility were used to trigger the rotation of the tube, as listed in the figure (from 10 to 1000 s).

The effects of long-lasting dynamic sleep deprivation shown in Figs. 4 and 6 were tested using the “optomotor module,” programmed with a 20-s immobility trigger. Once a week, flies were transferred to a fresh tube to ensure good quality food during the entire experiment. For the experiment shown in Fig. 1C, the behavior of both males and females was recorded for 7 days and then transferred to fresh tubes and recorded for another 7 days. To avoid confounding effects related to the location of the tube on sleep amount (e.g., an ethoscope and incubator), the new position of all the tubes was systematically interspersed (*60*). Namely, low and high sleepers from the same experiment and sex were paired as neighbors in a new arena, and their behavior was recorded for another week. Comparison was between days 2 to 7 and days 8 to 13, ignoring the first day and the day after the change of tube. For the experiment shown in Fig. 4, once a week, flies were transferred to a fresh tube to ensure good quality food during the entire experiment. For the experiment shown in Fig. 6, sleep deprivation was stopped after 9.5 days of treatment, and animals were allowed to recover for 3 days in the ethoscopes at 25°C to measure sleep rebound.

### Behavioral scoring

Immobility was scored by thresholding maximal velocity on 10-s epochs, as previously described (*24*). Sleep was computed using the so-called 5-min rule, according to which all immobility bouts longer than 300 s were counted as sleep bouts (including the first 300 s). During mechanical sleep deprivation, velocity measurements subsequent to stimuli were masked to avoid false positive of fly movement (*24*). Specifically, data in the 6 s following the onset of each rotation were not considered for sleep scoring. Sleep rebound shown in Fig. 5 (D and H) was expressed as the difference between the sleep amount measured during rebound and the expected sleep amount. Expected sleep amounts were inferred by a linear regression between the reference baseline sleep and sleep during the rebound period in the relevant control population. Formally, the homoeostatic rebound *H*_*i*_ of an individual *i* was expressed as

$$H_{i}=R_{i}-{\hat{R}}_{i}$$ (1)

$${\hat{R}}_{i}=\alpha +\beta B_{i}$$ (2)

where $\hat{R}$ is the predicted sleep after treatment (ZT ∈ [0, 3]), *R* is the measured sleep after treatment (ZT ∈ [0, 3]), *B* is the measured sleep before treatment (ZT ∈ [0, 3]), and α and β are the coefficients of the linear regression *R*_C_ = α + β*B*_C_ on the control group C.

$$\alpha ={\bar{R}}_{C}-\beta {\bar{B}}_{C}$$ (3)

$$\beta =\frac{\text{Cov}(R_{C},B_{C})}{\text{Var}(B_{C})}$$ (4)

Behavioral state (“quiescence,” “micromovement,” and “walking”) was defined for each consecutive minute of behavior (*B*) according to the following rule

$$B=\{\begin{array}{c} \text{quiescence},\\ \text{micromovement},\\ \text{walking}, \end{array}\begin{array}{c} \text{if}\;V_{\text{max}}<T_{v}\forall_{i}\\ \text{if}\;\Sigma^{i}|X_{i}-X_{i-1}|\\ \text{otherwise} \end{array}<T_{d}$$ (5)

where *V*_max_ is the maximum velocity, *T*_v_ is the validated threshold under which immobility is scored, *X* is the position along the tube, and *T*_d_ is a threshold of 15 mm on the distance moved above which walking is scored. *T*_d_ was defined empirically on the basis of the observation of a bimodal distribution of the total distance moved in a minute.

Because of the different amounts of food and cotton wool in each tube, the space available inside each experimental tube may be slightly different between individual animals. To compare the position of flies with respect to the boundary of their respective experimental environments, the animal longitudinal position was expressed relatively to the food (position = 0) and the cotton wool (position = 1) edges

$$\text{Position}\;=\frac{X-Q_{0.01}(X)}{Q_{0.99}(X-Q_{0.01}(X))}$$ (6)

where *Q*_*n*_ is the quantile function.

First and last percentiles were used instead of minimum and maximum to avoid the possible effect of spurious artifactual detections beyond physical limits of the tube.

### Sleep versus life-span regression

The linear regression to predict life span from the amount of sleep (Fig. 4D) had the form

Life span = Sleep × Sex (7)

To remove the abnormal data that precede immediately the death of flies, we measured sleep amount (sleep) as the average proportion of time asleep over the first 10 days for untreated animals that lived at least 20 days. Right-censored animals (e.g., that were accidentally lost) were excluded from this analysis.

### Dendrograms and hierarchical clustering

The dendrograms in Fig. 3D and fig. S2A are the result of a hierarchical clustering using the UPGMA (unweighted pair group method with arithmetic mean) method (*61*). During an interval of time, the proportion of time spent by an animal in a behavioral state can be formulated as an empirical discrete probability density function. In this context, the distance between each pair of animal was computed using the average of Bhattacharyya distances (*62*) over the entire day

$$D(p,q)=\frac{\sum_{t\in T}{\text{BD}}_{t}(p_{t},q_{t})}{\left|T\right|}$$ (8)

$${\text{BD}}_{t}(p_{t},q_{t})=-\text{ln}(\text{BC}(p_{t},q_{t}))$$ (9)

$$\text{BC}(p_{t},q_{t})=\sum_{x\in X}\sqrt{pt(x)qt(x)}$$ (10)

where BD_*t*_ is the Bhattacharyya distance at a time interval *t*; *T* is the set of all tested time intervals: *T* = {[0, 0.25), [0.25, 0.5), …, [23.75, 24)} hours; BC_*t*_ is the Bhattacharyya coefficient at a time interval *t*; *p* and *q* are the observed distributions of behavior for two different individuals; and *X* is the set of discrete behaviors: *X* = {quiescent, micromovements, walking}.

### Statistics

Unless otherwise stated, the shaded areas around the mean (e.g., Fig. 3, A and B) and the error bars (e.g., Fig. 5, B to D and F to H) are 95% CI computed using basic bootstrap resampling (*63*) with *n* = 1000. Median life-span CIs were estimated accounting for censored data (*64*).

#### Regressions

The lines in Figs. 1C and 2B are linear regression, and the shaded areas are 95% parametric CIs.

#### Survival curves

Figure 4 (B and C) shows Kaplan-Meier curves, and the shaded areas represent 95% CIs.

#### Software

All data analyses were performed in R using the rethomics framework (*65*). Figures were drawn using ggplot2 (*66*), and ternary representations in Figs. 2E and 3C were generated with ggtern (*67*).

## Supplementary Material

http://advances.sciencemag.org/cgi/content/full/5/2/eaau9253/DC1

## SUPPLEMENTARY MATERIALS

Supplementary material for this article is available at http://advances.sciencemag.org/cgi/content/full/5/2/eaau9253/DC1 Fig. S1. Representative tracings of the behavioral activity over the course of 48 hours as recorded in real time by ethoscopes for all 881 female flies shown in Fig. 1A. Fig. S2. Sorted hierarchical cluster analysis based on pairwise distance, as supplement to Fig. 3. Fig. S3. Decrease in locomotion activity in sleep-deprived flies over time, a possible sign of physical fatigue. Fig. S4. Circadian rhythm, and not homeostatic drive, is the major contributor to sleep pressure during long-term sleep deprivation. Movie S1. Visual representation of the distribution of behavioral features across 24 hours in the dataset shown in Figs. 1 (A and B) and 2.
