# Supplementary material for: Most sleep does not serve a vital function: Evidence from Drosophila melanogaster
Source: Sci Adv. 2019 Feb 20;5(2):eaau9253. doi: 10.1126/sciadv.aau9253 (PMC6382397; doi:10.1126/sciadv.aau9253)
Supplement: http://advances.sciencemag.org/cgi/content/full/5/2/eaau9253/DC1 [file supp_5_2_eaau9253__index.html]

Science Advances | Science Advances

## Supplementary Materials

**The PDF file includes:**

- Legend for fig. S1
- Fig. S2. Sorted hierarchical cluster analysis based on pairwise distance, as supplement to Fig. 3.
- Fig. S3. Decrease in locomotion activity in sleep-deprived flies over time, a possible sign of physical fatigue.
- Fig. S4. Circadian rhythm, and not homeostatic drive, is the major contributor to sleep pressure during long-term sleep deprivation.
- Legend for movie S1

Download PDF

**Other Supplementary Material for this manuscript includes the following:**

- Fig. S1 (.pdf format). Representative tracings of the behavioral activity over the course of 48 hours as recorded in real time by ethoscopes for all 881 female flies shown in Fig. 1A.
- Movie S1 (.mov format). Visual representation of the distribution of behavioral features across 24 hours in the dataset shown in Figs. 1 (A and B) and 2.

**Files in this Data Supplement:**

- Adobe PDF - aau9253\_SM.pdf
- Adobe PDF - aau9253\_Figure\_S1.pdf
