# Supplementary material for: Most sleep does not serve a vital function: Evidence from Drosophila melanogaster
Source: Sci Adv. 2019 Feb 20;5(2):eaau9253. doi: 10.1126/sciadv.aau9253 (PMC6382397; doi:10.1126/sciadv.aau9253)
Supplement: http://advances.sciencemag.org/cgi/content/full/5/2/eaau9253/DC1 [file aau9253_SM.pdf]

Science Advances
AAAS

advances.sciencemag.org/cgi/content/full/5/2/eaau9253/DC1

Supplementary Materials for

Most sleep does not serve a vital function: Evidence from *Drosophila melanogaster*

Quentin Geissmann, Esteban J. Beckwith, Giorgio F. Gilestro*

*Corresponding author. Email: giorgio@gilest.ro

Published 20 February 2019, *Sci. Adv.* **5**, eaau9253 (2019)
DOI: 10.1126/sciadv.aau9253

The PDF file includes:

Legend for fig. S1
Fig. S2. Sorted hierarchical cluster analysis based on pairwise distance, as supplement to Fig. 3.
Fig. S3. Decrease in locomotion activity in sleep-deprived flies over time, a possible sign of physical fatigue.
Fig. S4. Circadian rhythm, and not homeostatic drive, is the major contributor to sleep pressure during long-term sleep deprivation.
Legend for movie S1

Other Supplementary Material for this manuscript includes the following:

(available at advances.sciencemag.org/cgi/content/full/5/2/eaau9253/DC1)

Fig. S1 (.pdf format). Representative tracings of the behavioral activity over the course of 48 hours as recorded in real time by ethoscopes for all 881 female flies shown in Fig. 1A.
Movie S1 (.mov format). Visual representation of the distribution of behavioral features across 24 hours in the dataset shown in Figs. 1 (A and B) and 2.

Fig. S1. Representative tracings of the behavioural activity over the course of 48 hours as recorded in real time by the ethoscopes for all 881 female flies shown in Fig. 1A. (A) Each panel shows the activity data for a different female individual fly, sorted in descending order from long sleepers to short sleepers. The continuous black line plots the position of the flies along the tube (y axis) over time (x axis). The transversal dashed line represents the position of a virtual infrared beam (*31*). The colour on the background highlights the simultaneous behavioural classification with a resolution of 1 minute (grey: quiescent, green: micro-moving; blue: walking).

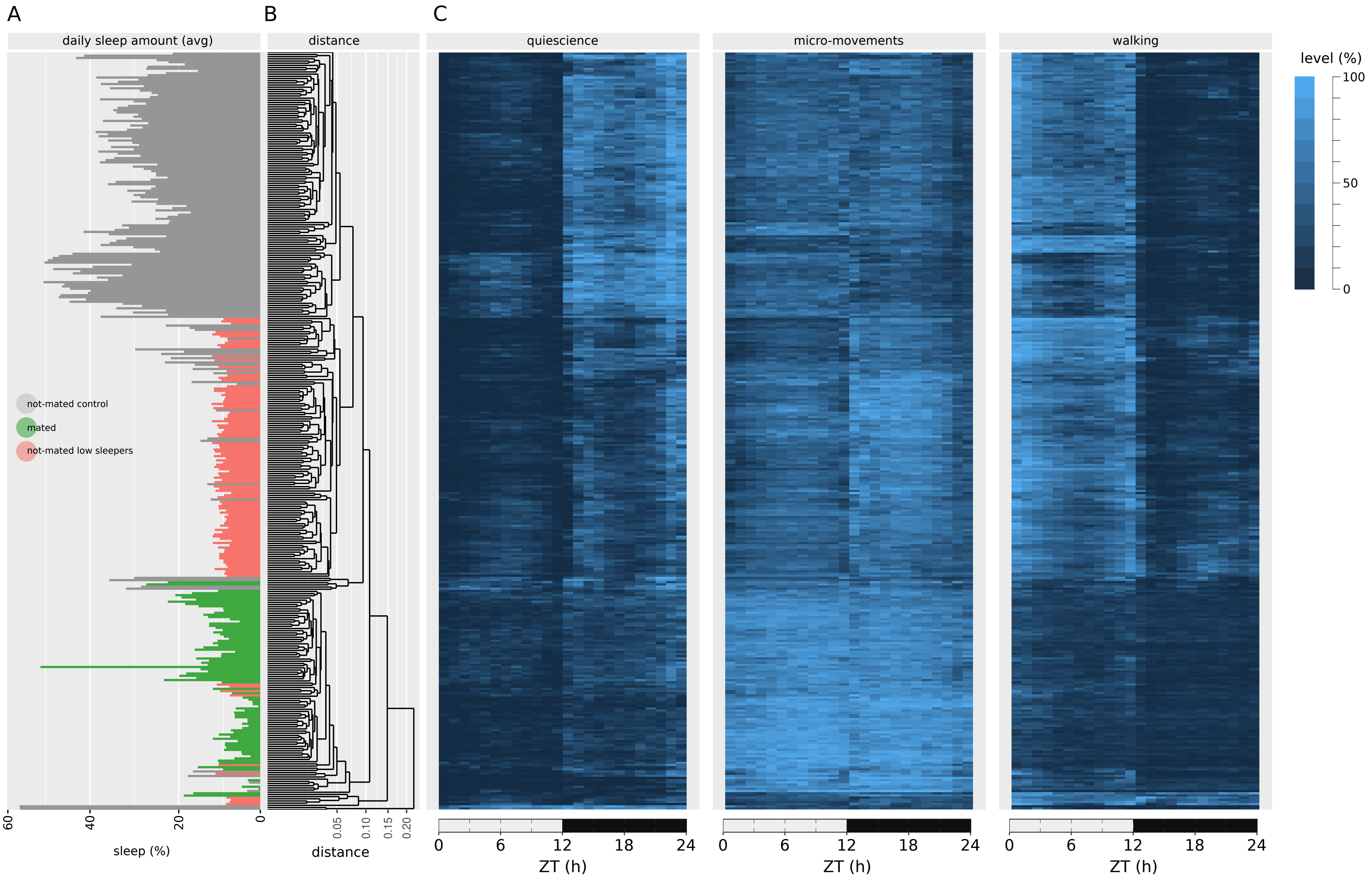

A
B
C
daily sleep amount (avg)
distance
quiescience
micro-movements
walking
level (%)
100
50
0
not-mated control
mated
not-mated low sleepers
60
40
20
0
sleep (%)
0.05
0.10
0.15
0.20
distance
0
6
12
18
24
ZT (h)

Fig. S2. Sorted hierarchical cluster analysis based on pairwise distance, as supplement to Fig. 3. (**A**) Average daily sleep amount as % of the day for each female fly in the dataset. Same colour code as Figure 3. (**B**) Hierarchical clustering dendrogram based on pairwise distance. (**C**) Visual representation of the average occurrence of the three behavioural features in each single animal across the 24 hour period.

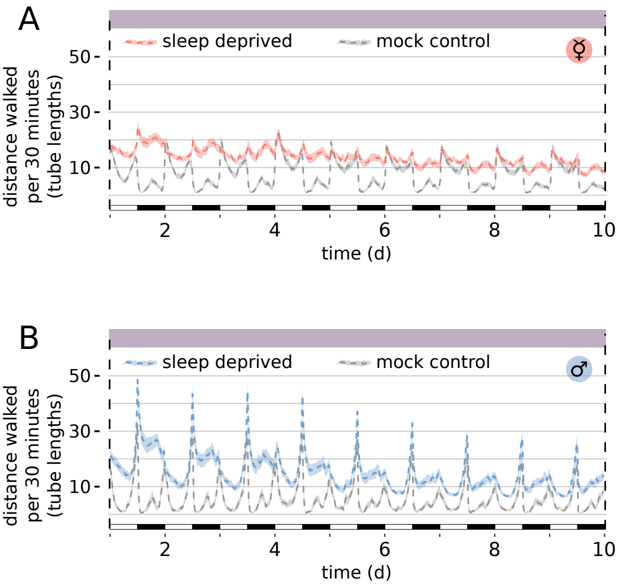

Fig. S3. Decrease in locomotion activity in sleep-deprived flies over time, a possible sign of physical fatigue. Walking profile of female (**A**) and male (**B**) CantonS flies during the 9.5 d sleep deprivation experiment shown in Fig. 6. Grey indicates the sex-matching control animals. The dashed line indicates the distance walked in 30 minutes. Same dataset as in Figure 6A, B.

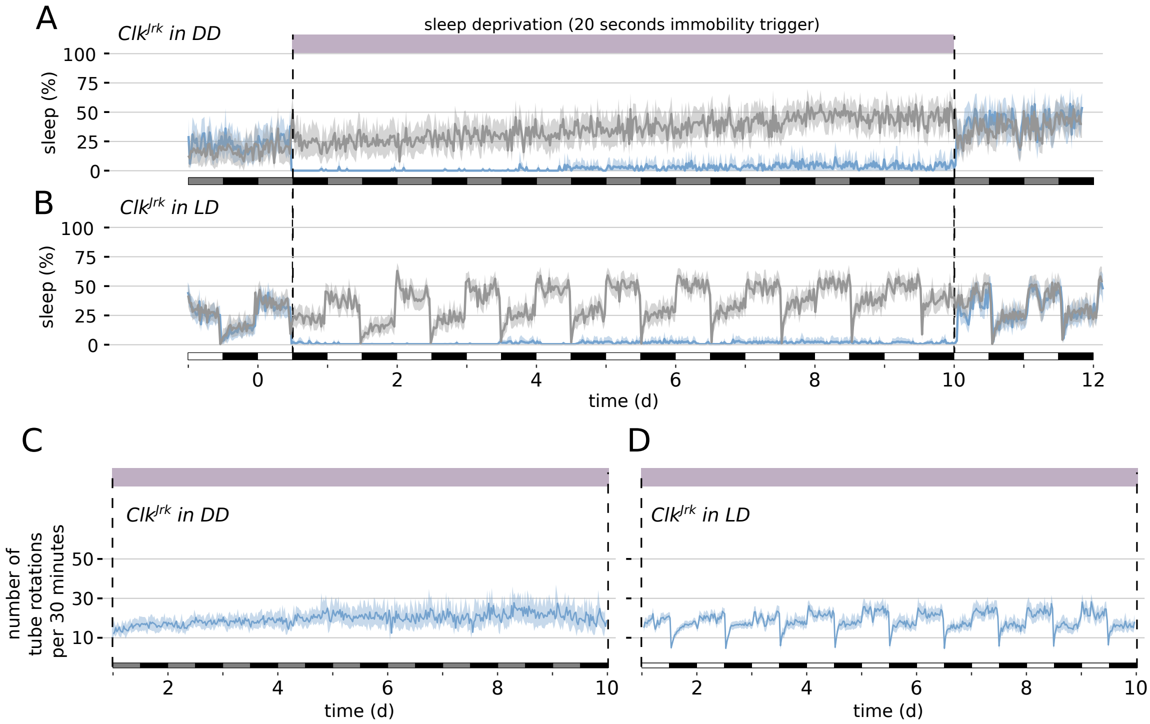

Fig. S4. Circadian rhythm, and not homeostatic drive, is the major contributor to sleep pressure during long-term sleep deprivation. Sleep profile for *Clk*^{Jrk} male flies under constant dark conditions (DD, A) or under 12 h light: 12 h dark conditions (LD, B) during the length of the experiment compared with their undisturbed mock controls (grey in both). Day 0 signs the beginning of the chronic sleep deprivation procedure, lasting 228 hours (indicated by a purple shade on top). (**C**, **D**) Average number of tubes rotations over the length of the sleep deprivation experiment (dashed lines) under DD (C) or LD (D) conditions. Ns = [18,20] for the DD experiments and [39,40] for LD.

Movie S1. Visual representation of the distribution of behavioral features across the 24 hours in the dataset shown in Figs. 1 (A and B) and 2. Each frame in the movie bins data with a 15 minutes resolution. Red: female flies; Blue: male flies. In the leftmost panels, each dot is an individual animal plotted in their behavioural space at that time point. The right most panel shows the 24 h distribution of the following four features: fraction of quiescent, micro-moving, walking animals (top three) and average position along the tube longitudinal length (bottom).
