# Supplementary figures and images for: Most sleep does not serve a vital function: Evidence from Drosophila melanogaster
Source: Sci Adv. 2019 Feb 20;5(2):eaau9253. doi: 10.1126/sciadv.aau9253 (PMC6382397; doi:10.1126/sciadv.aau9253)

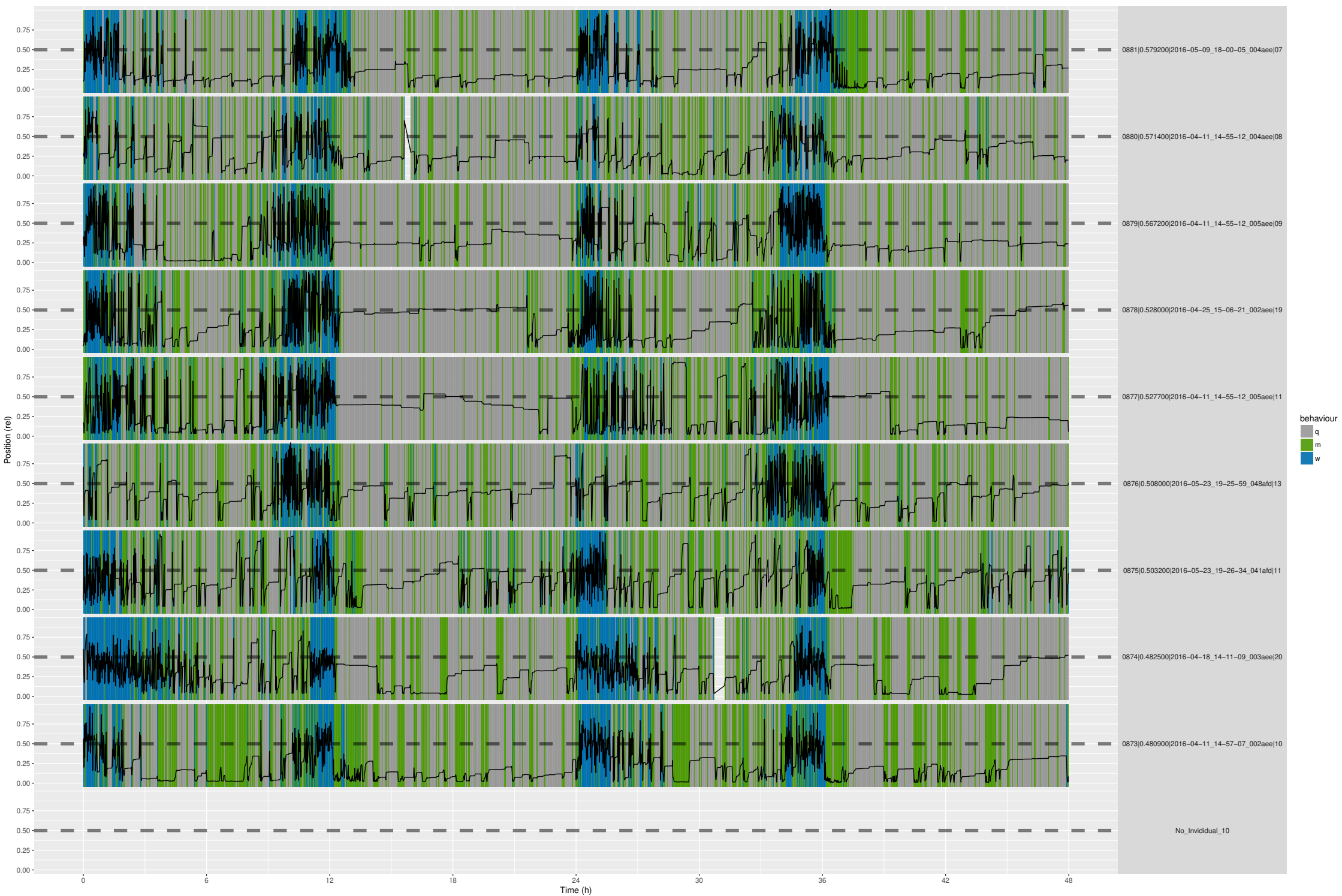

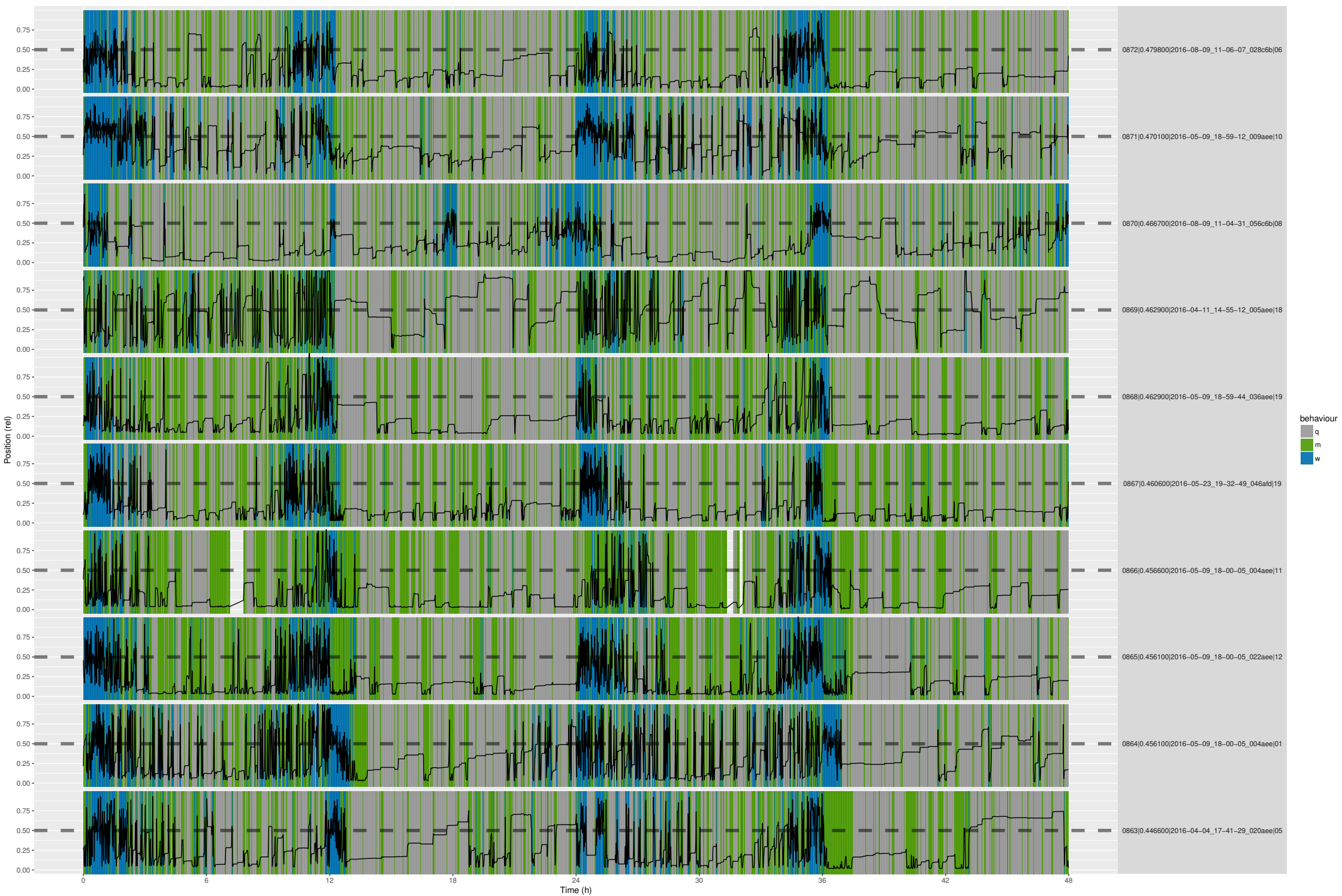

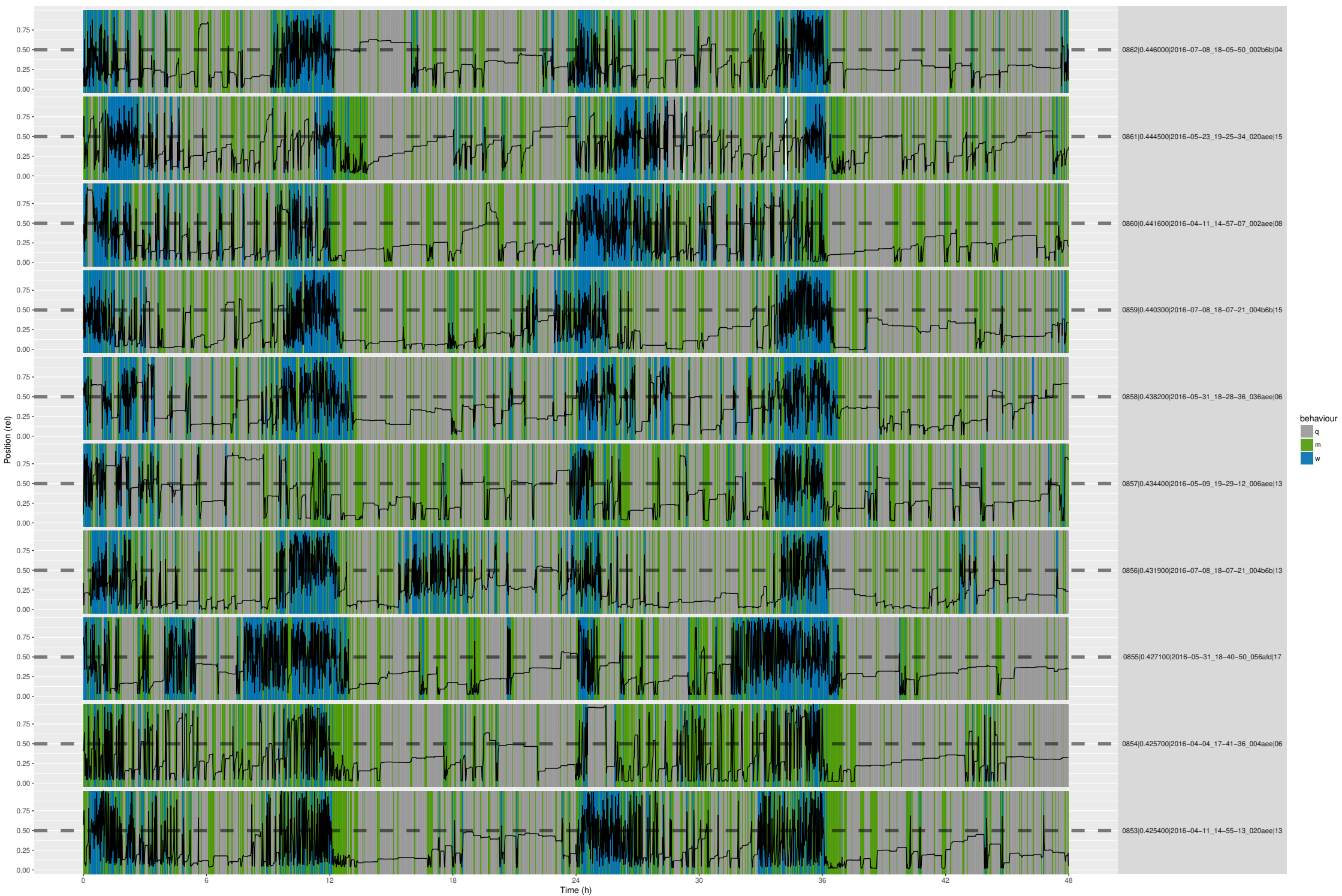

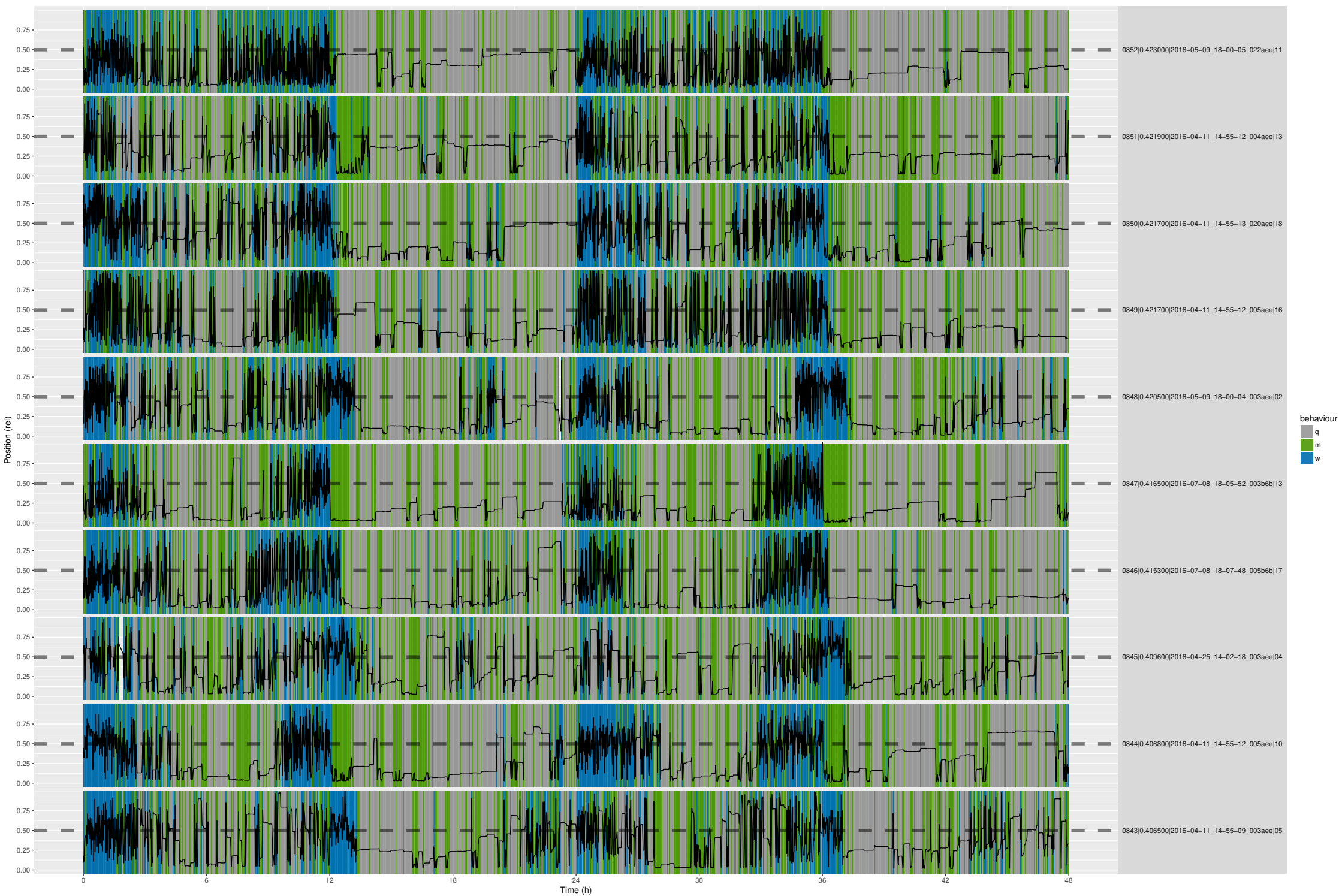

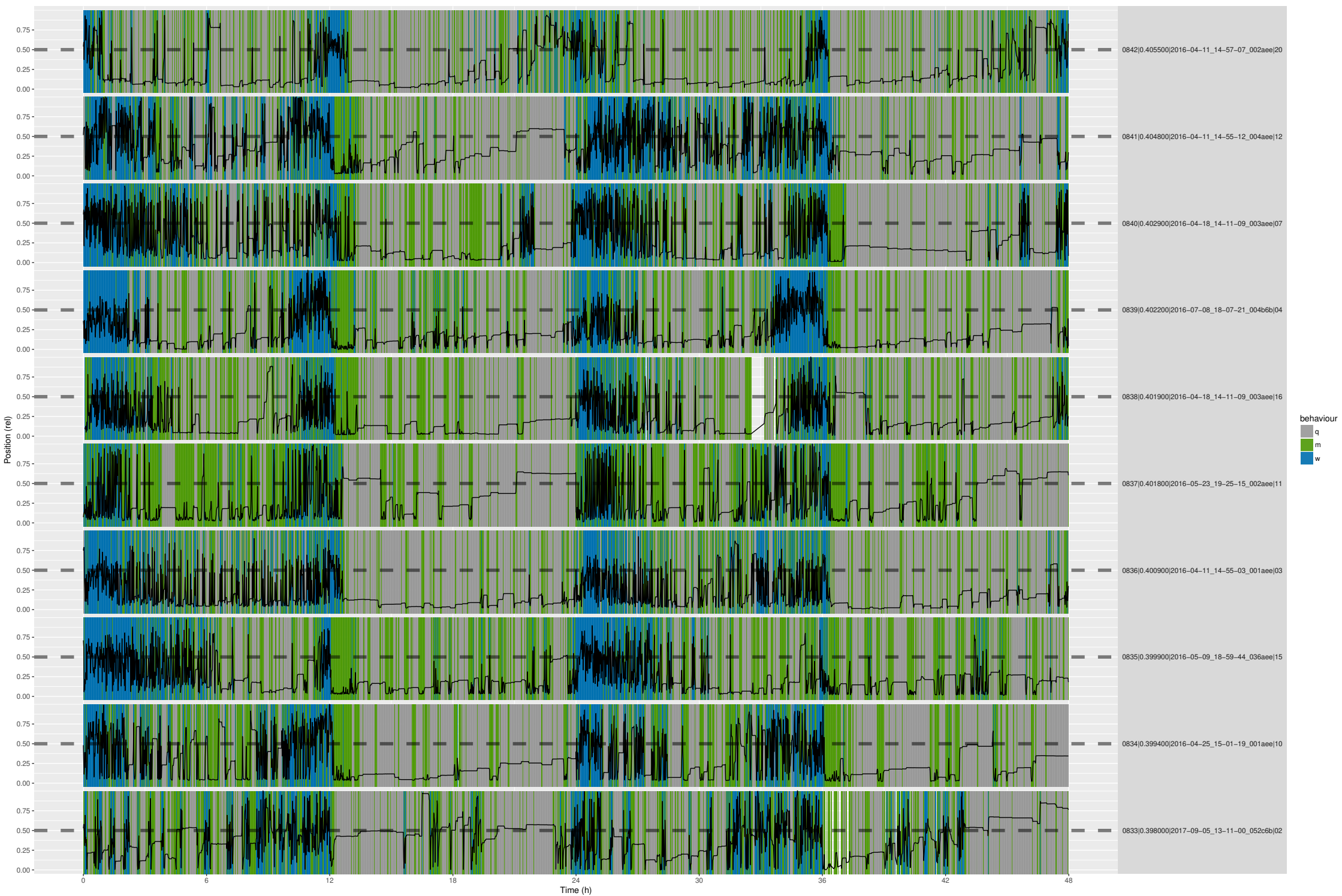

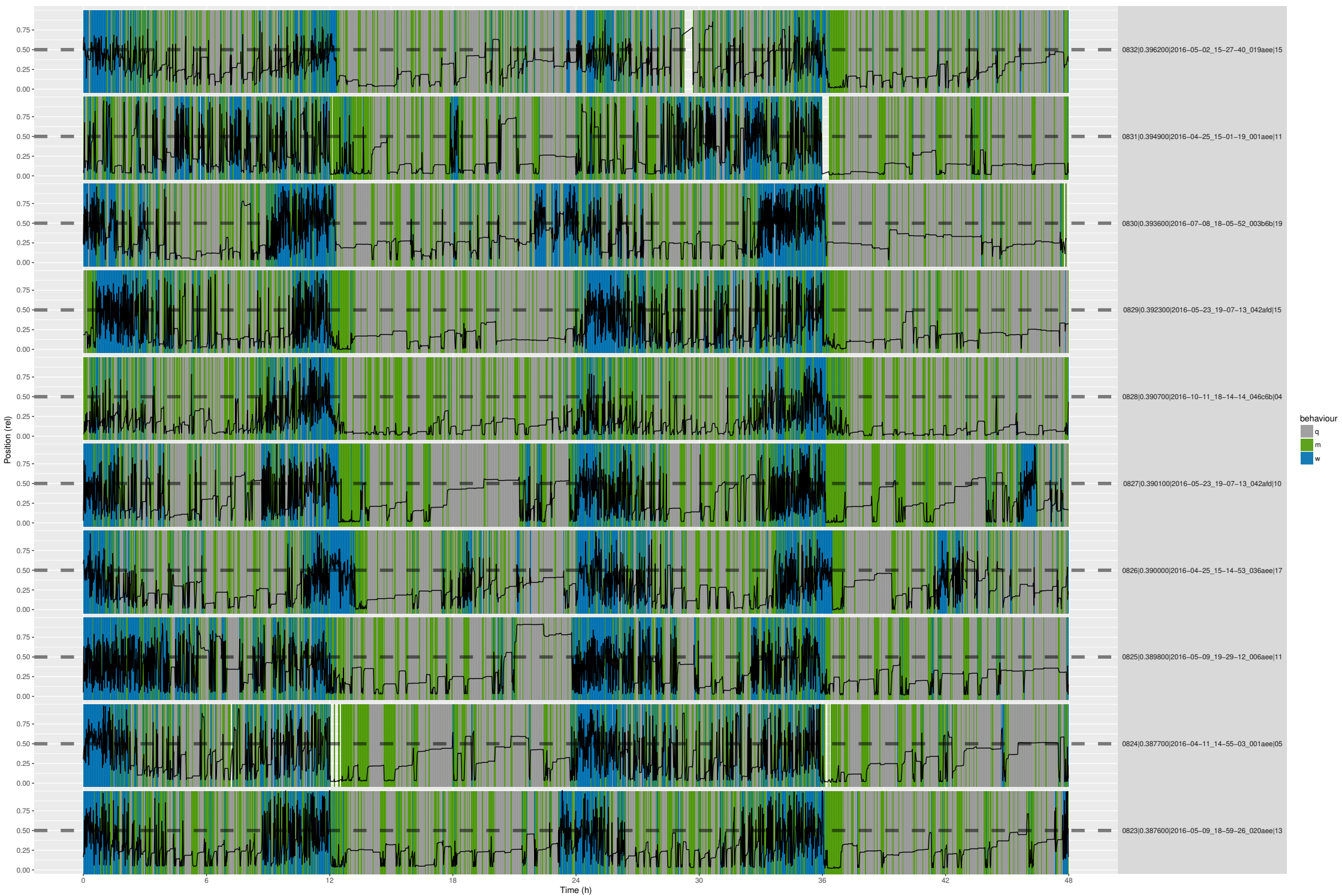

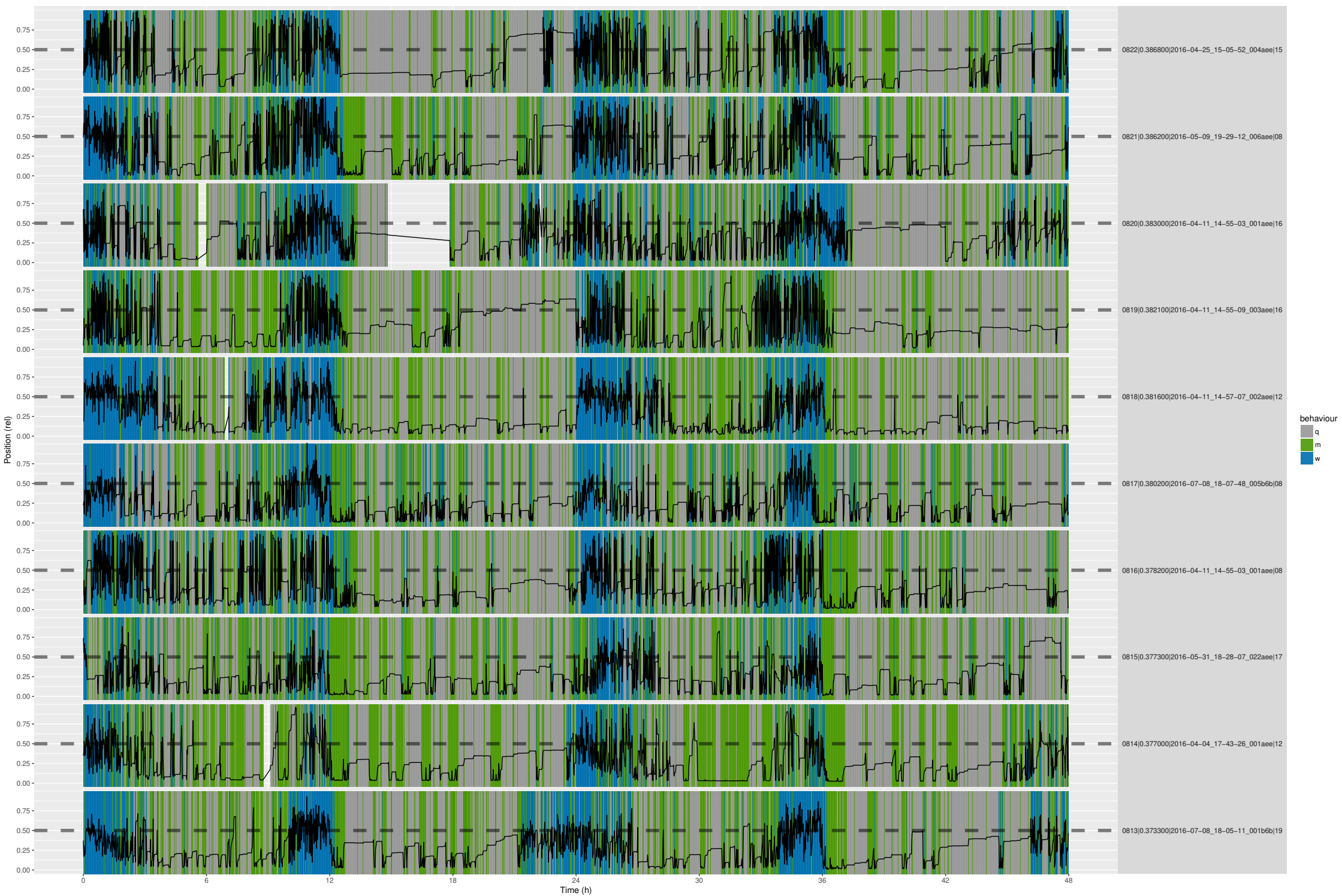

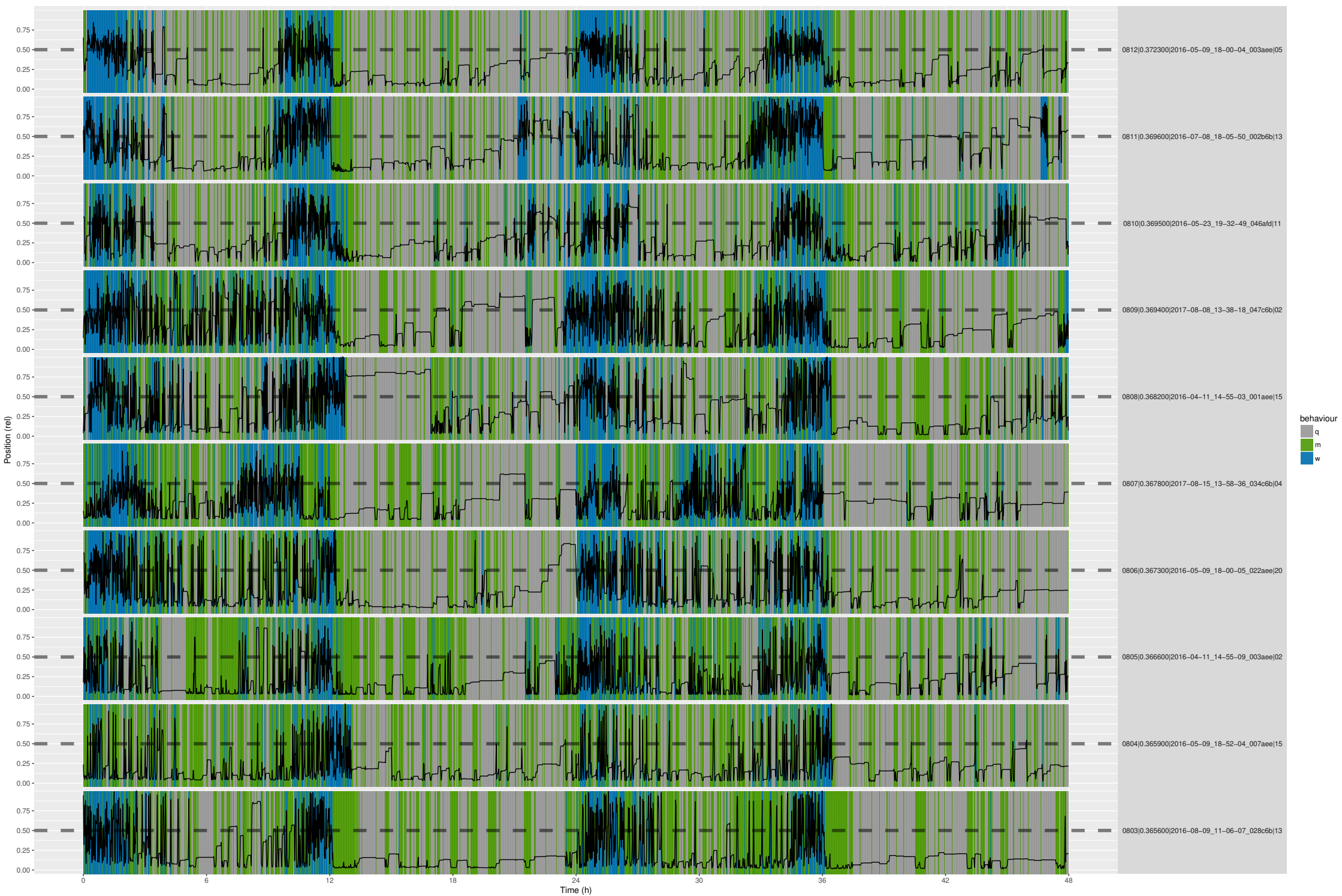

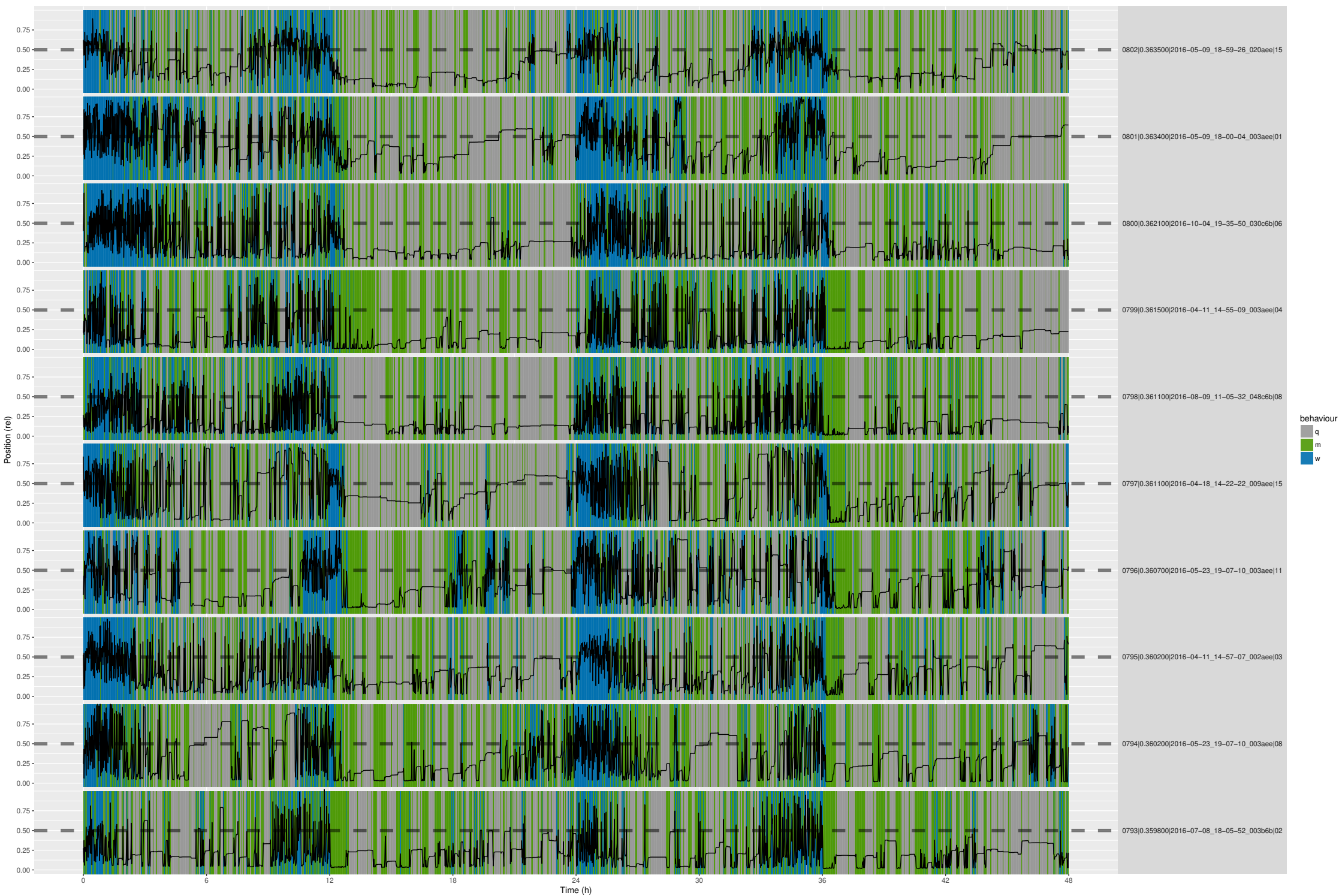

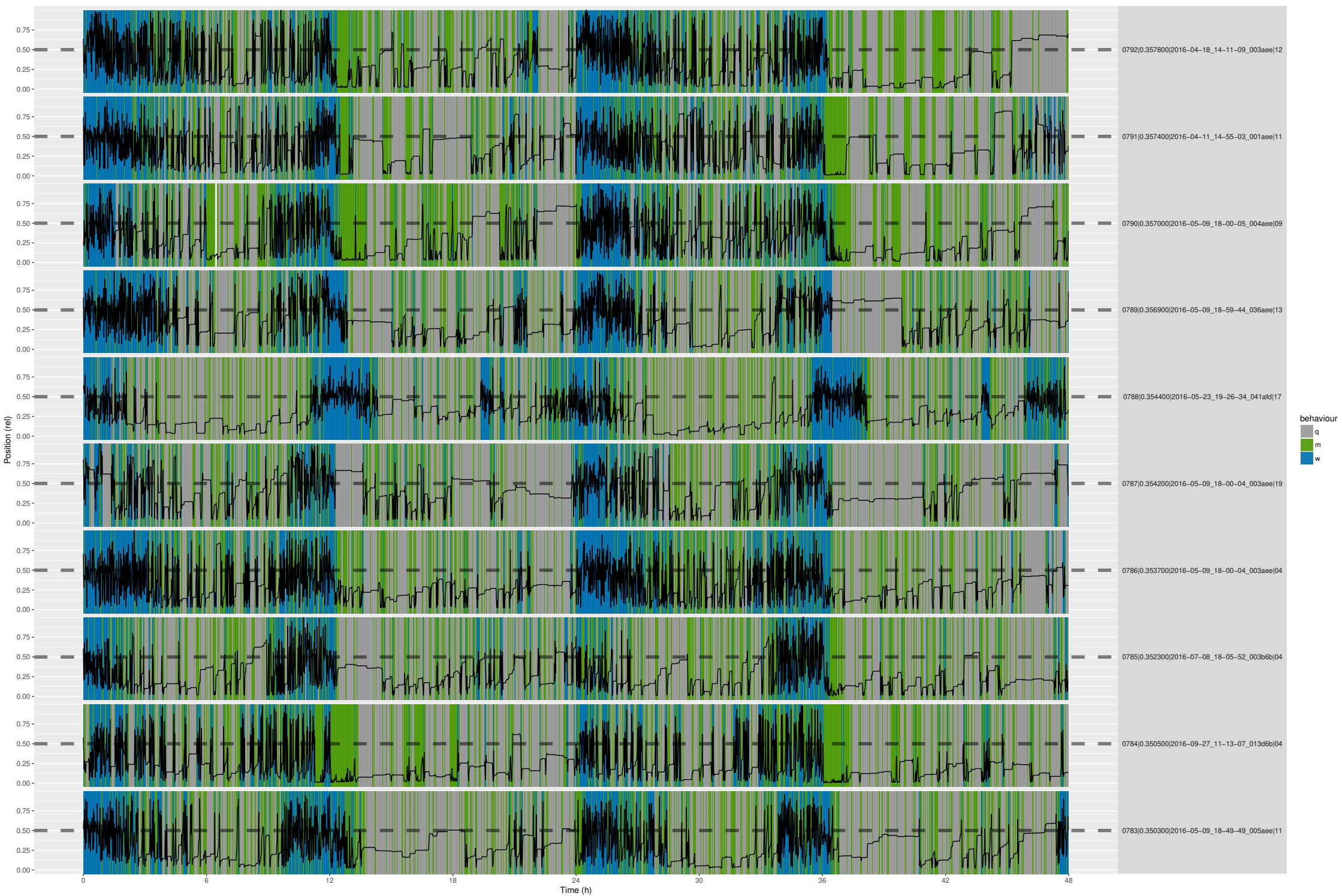

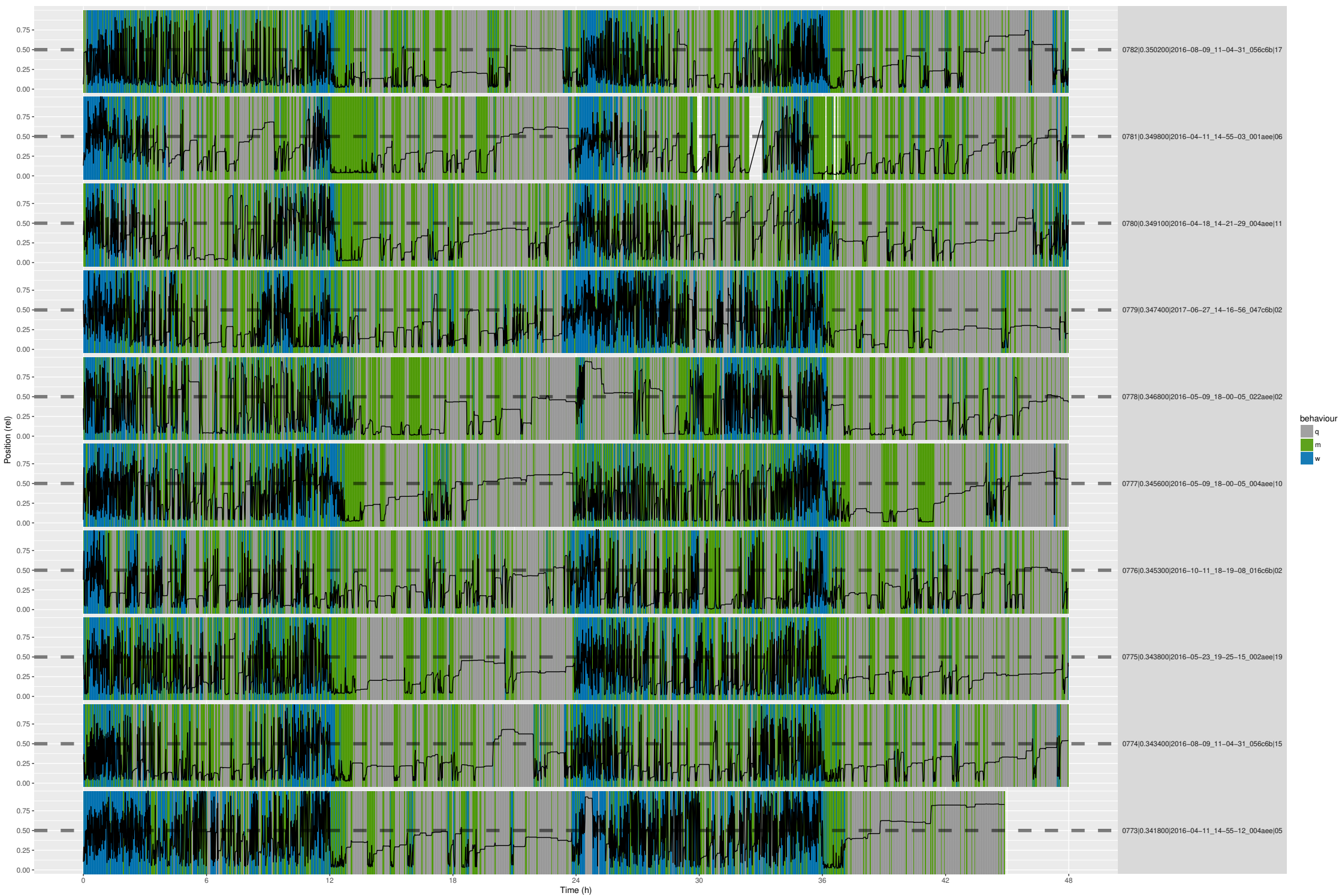

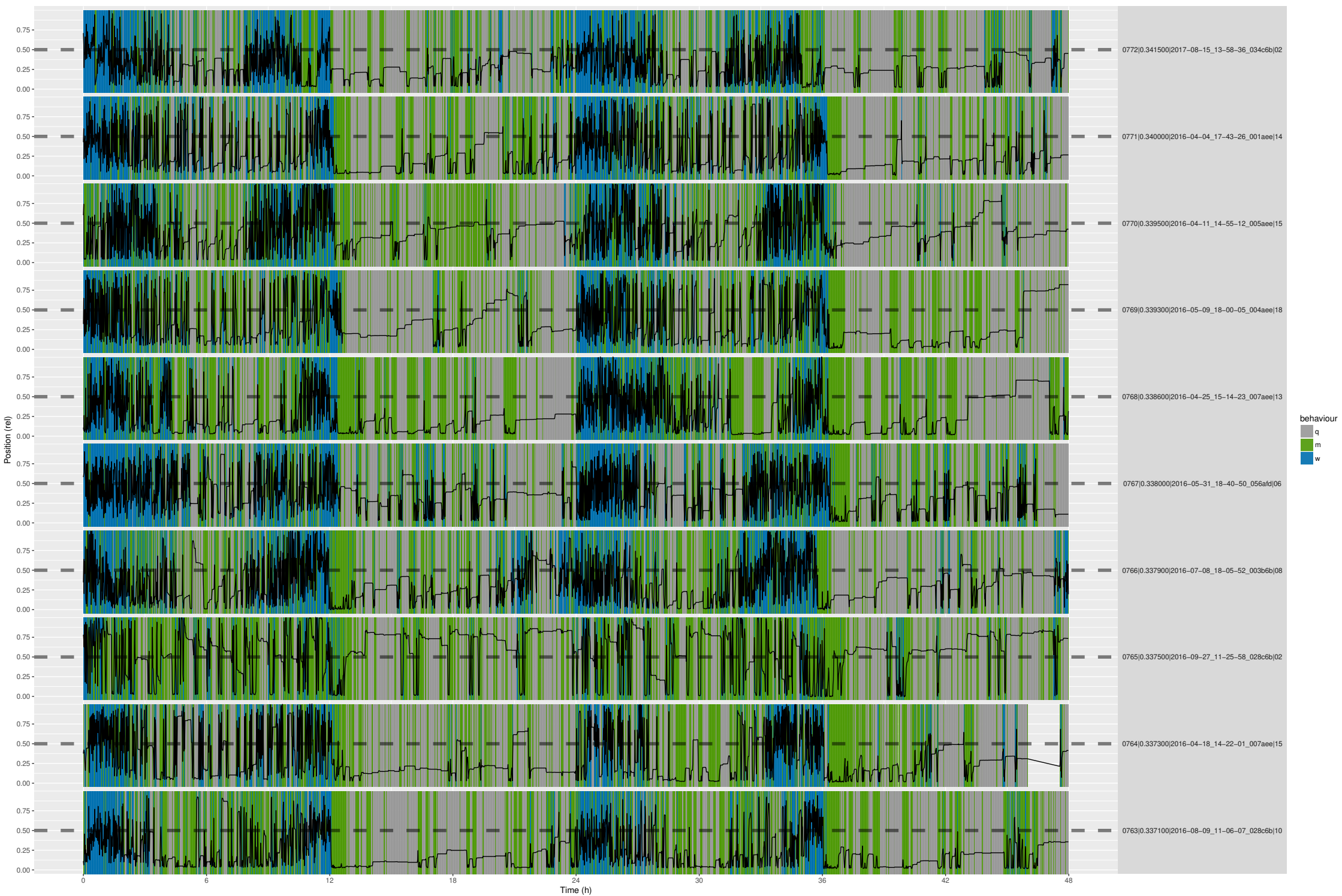

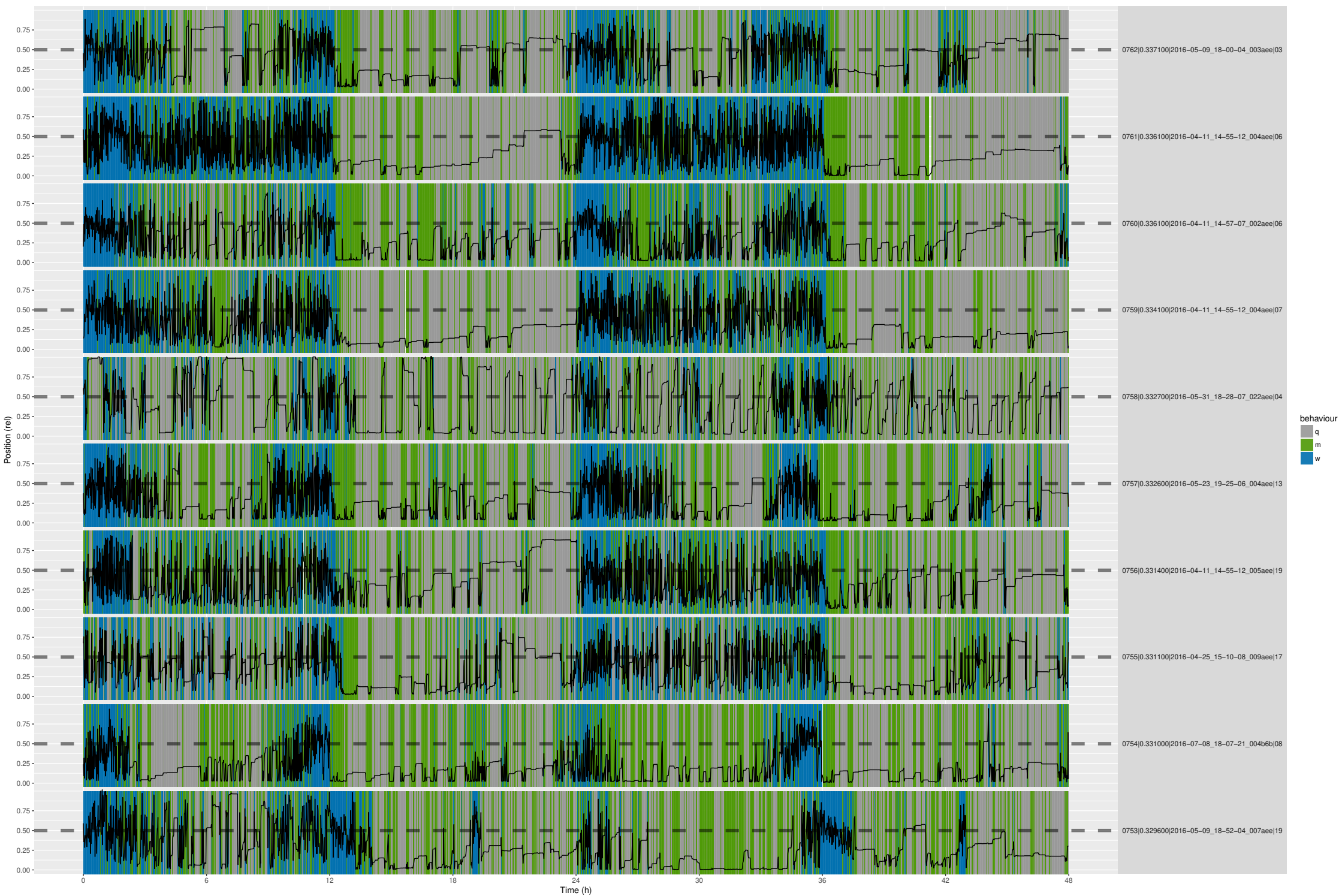

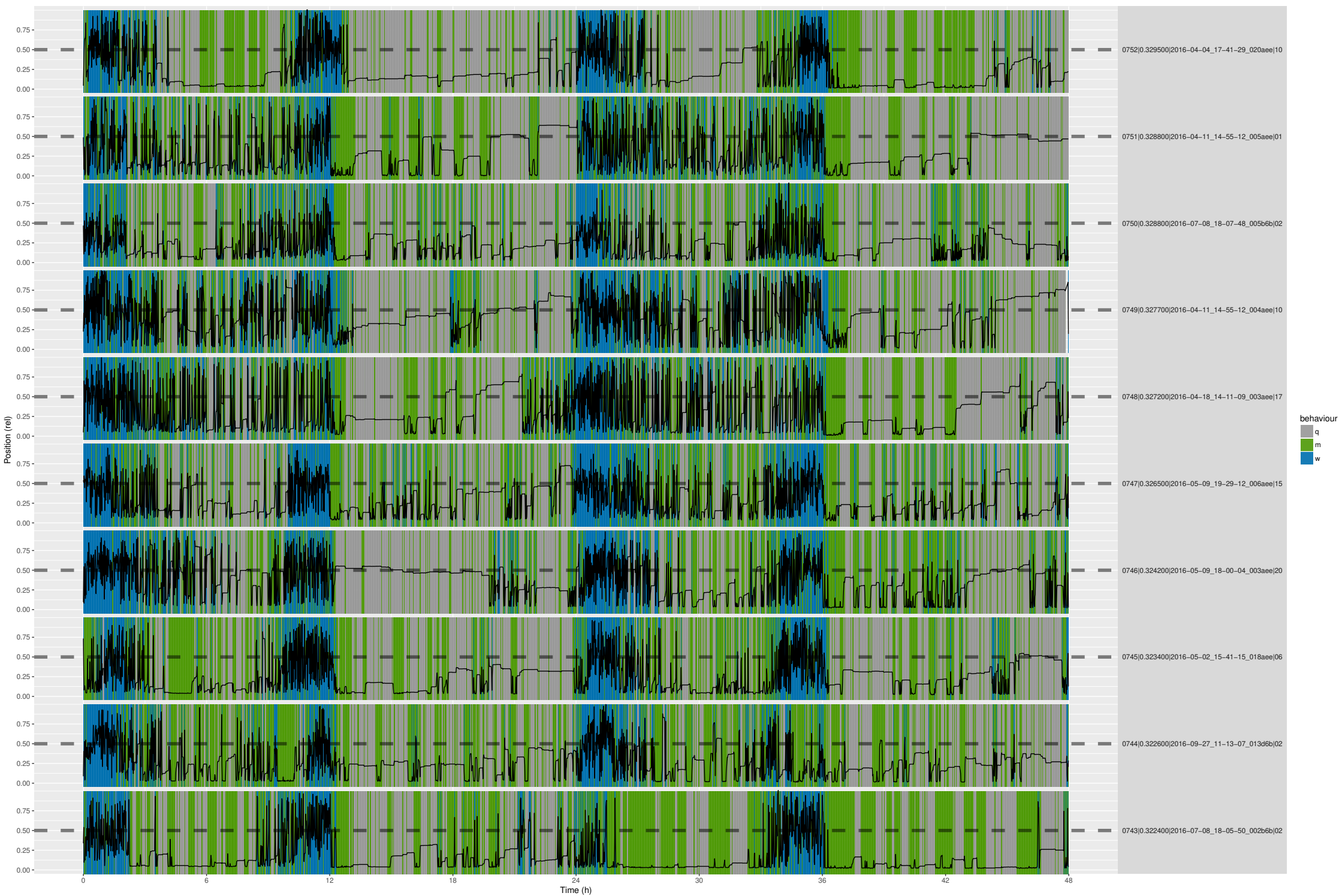

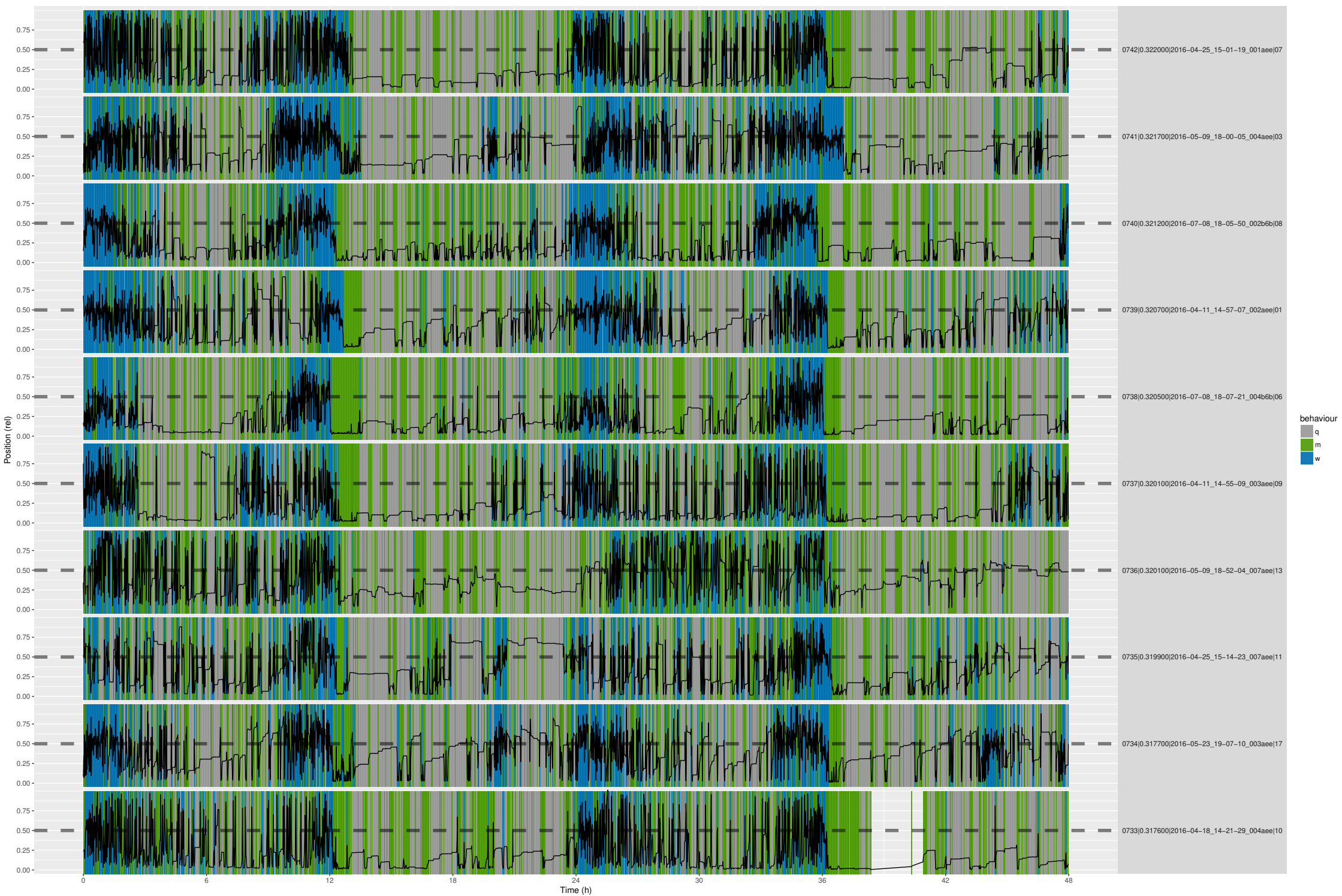

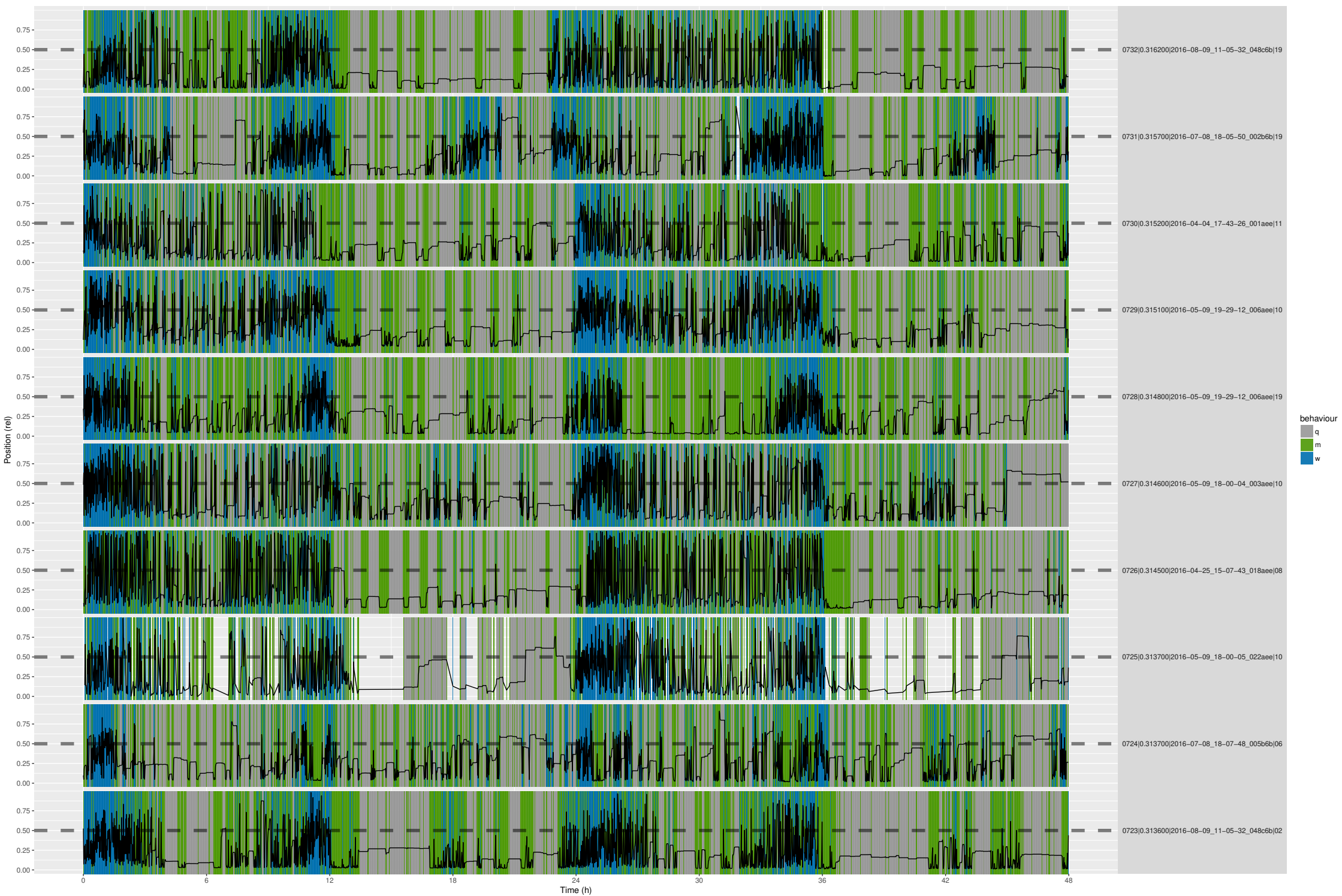

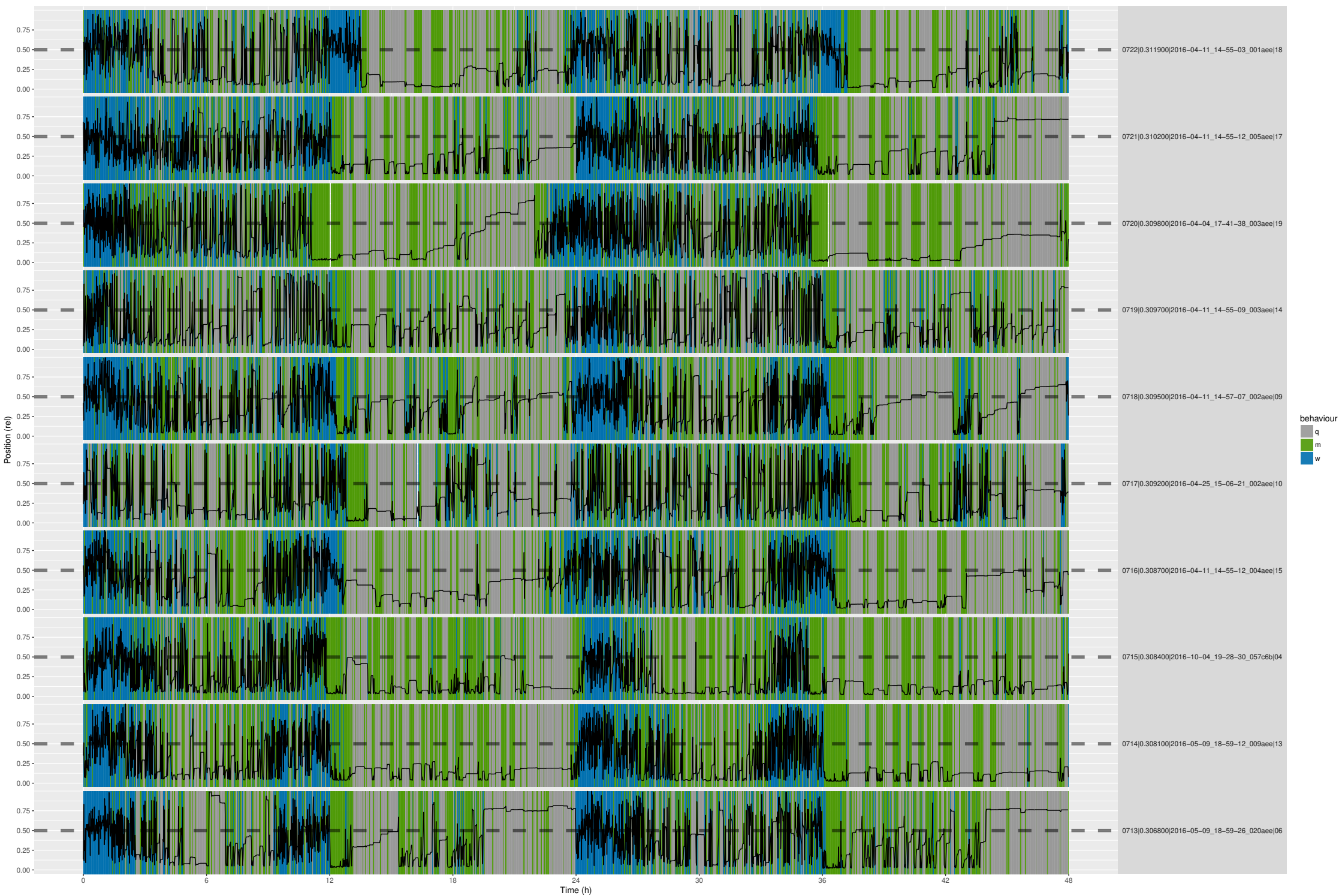

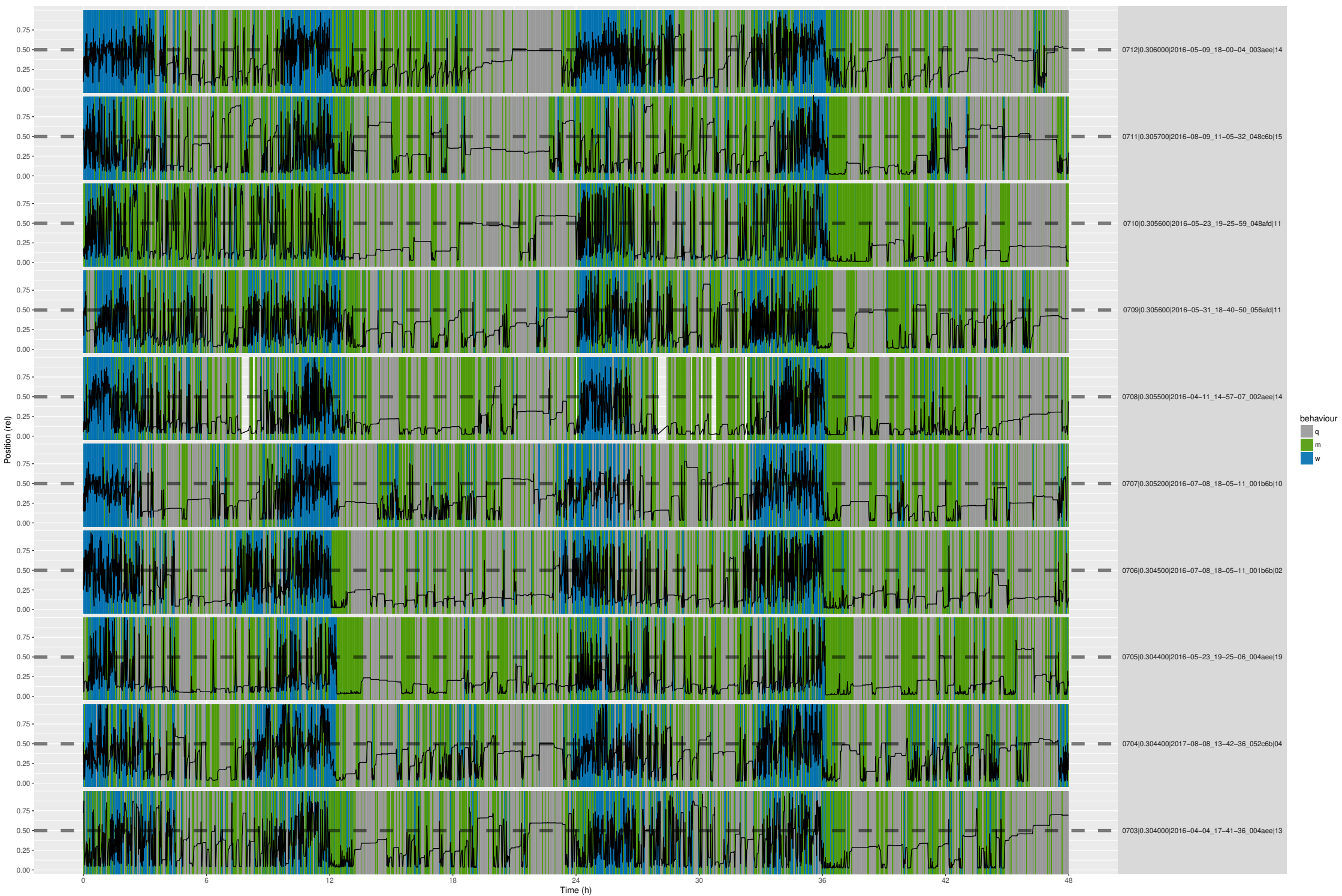

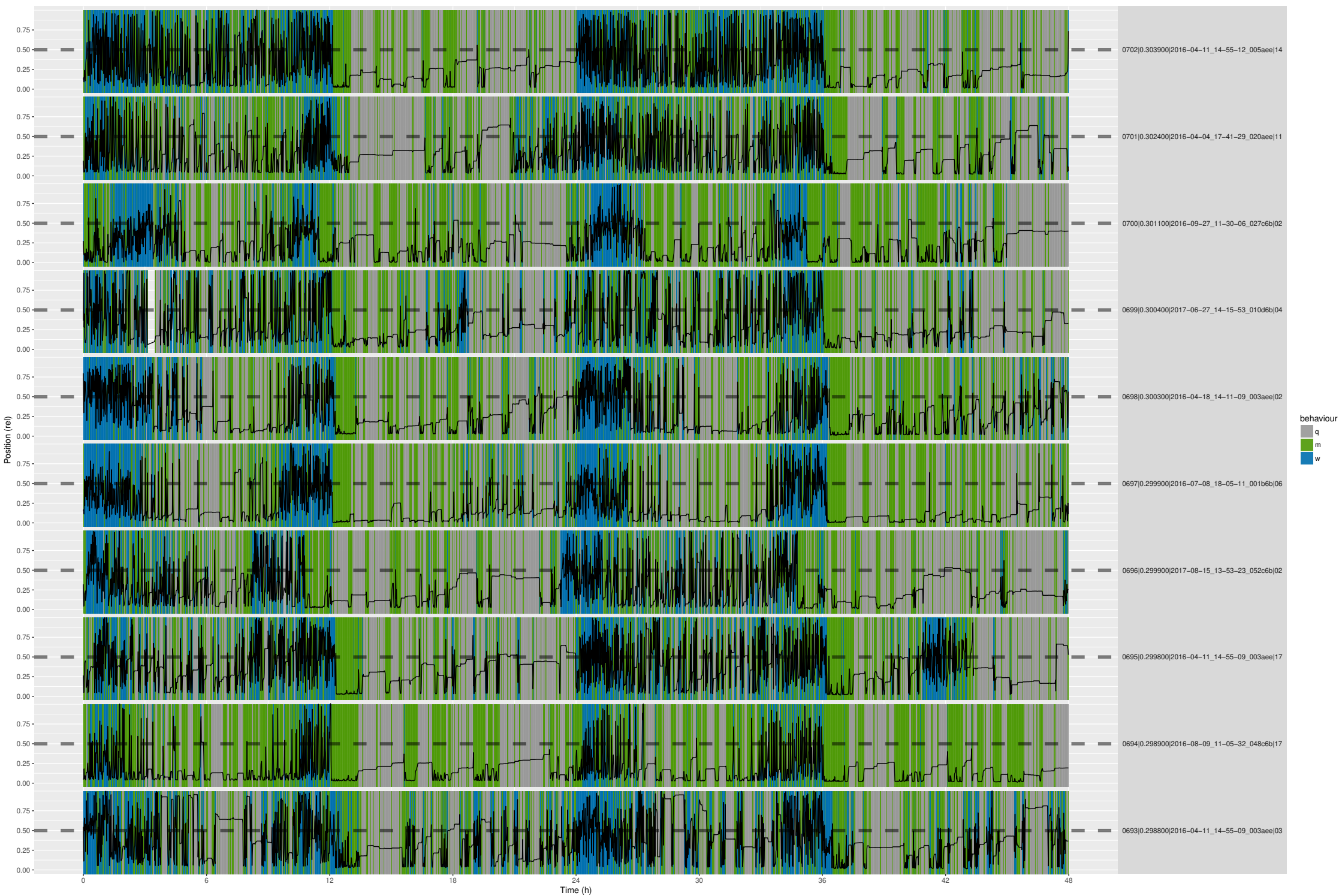

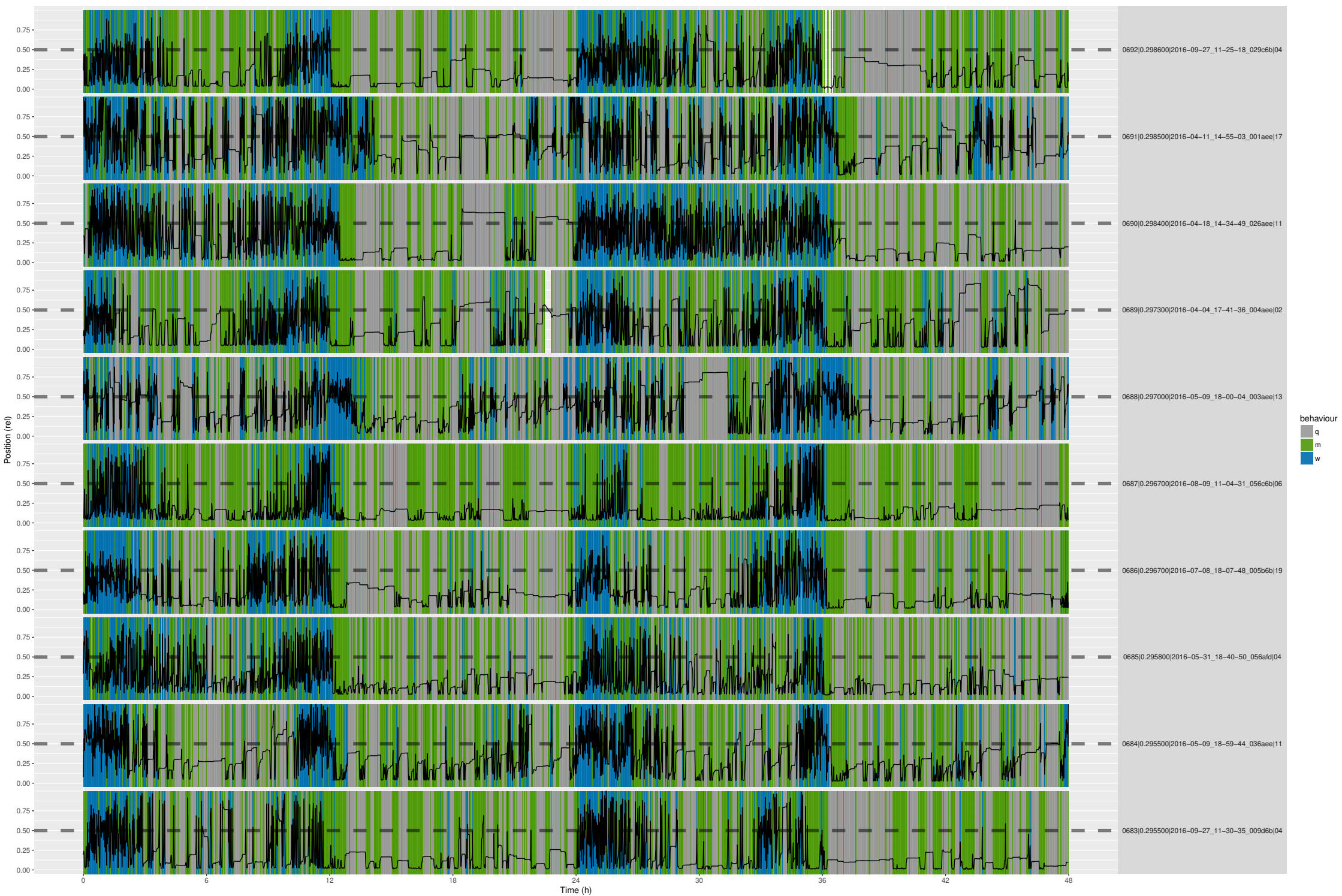

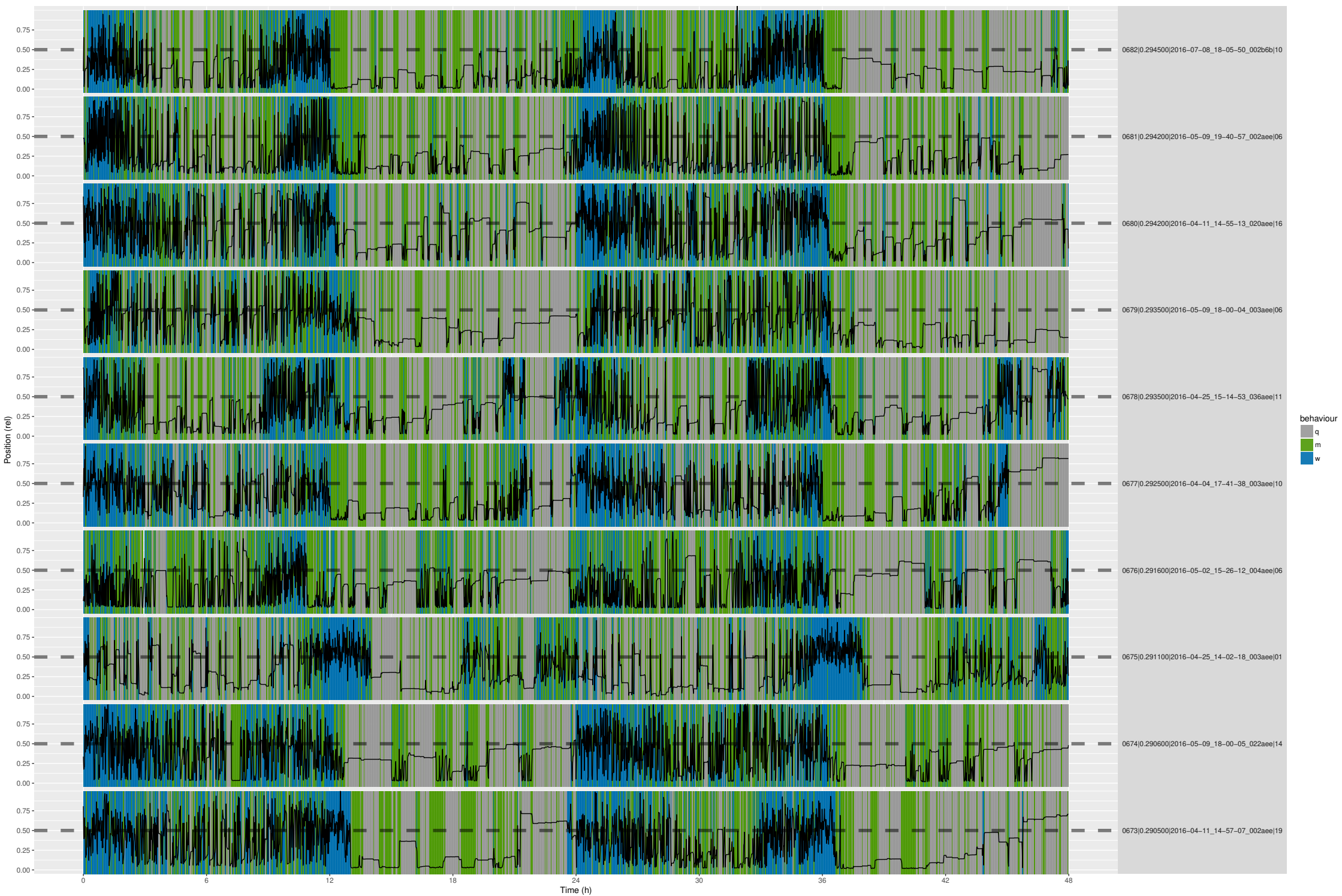

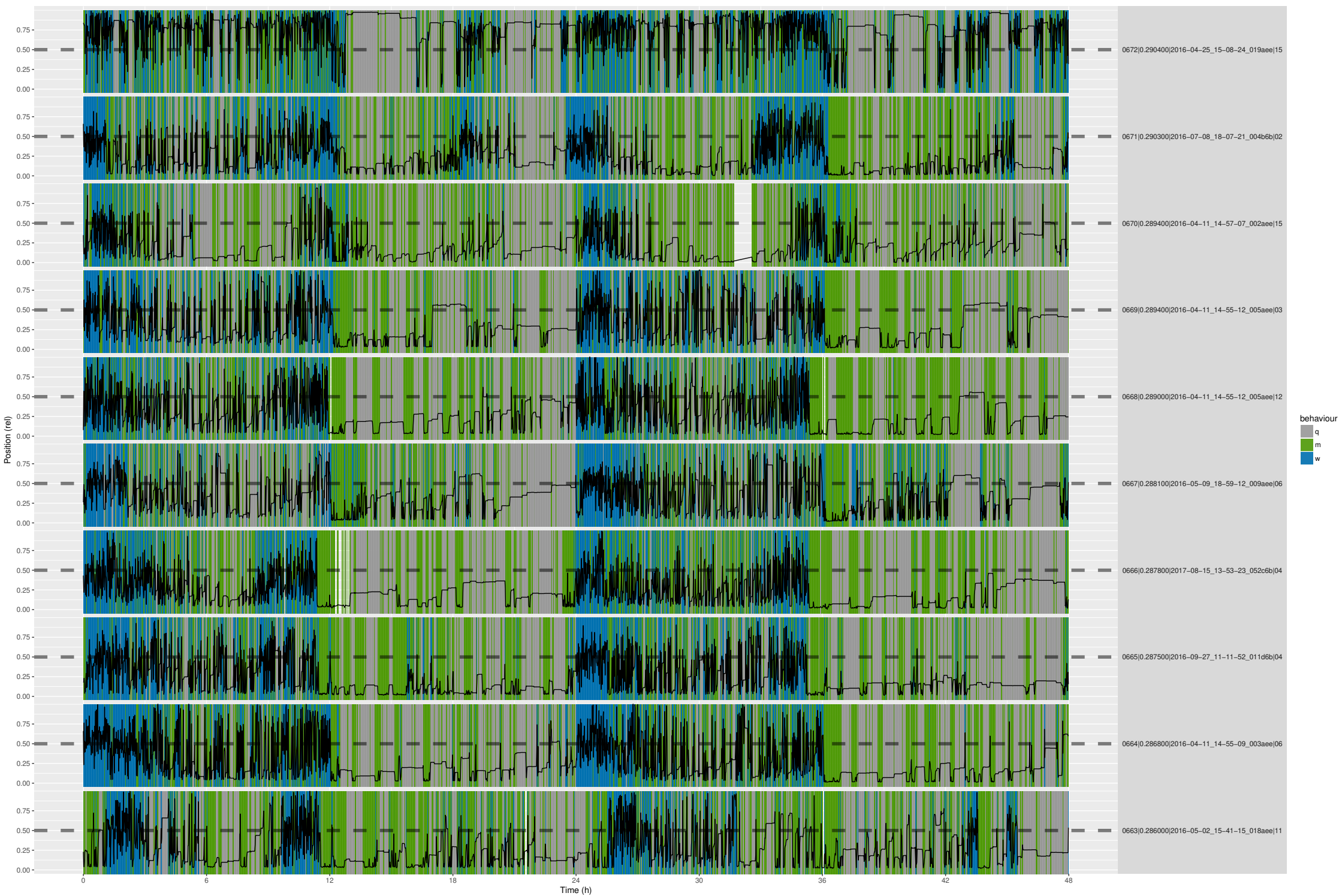

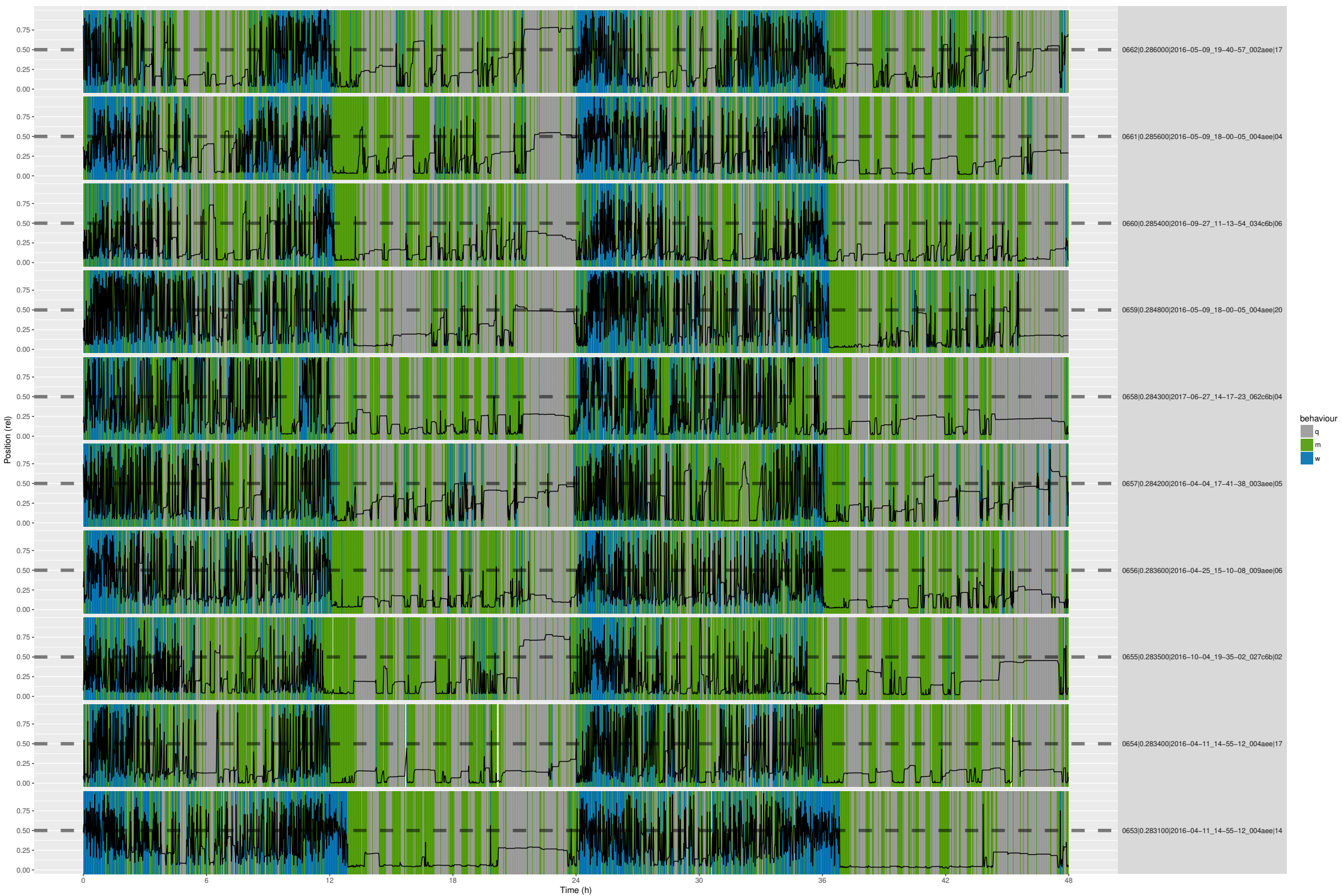

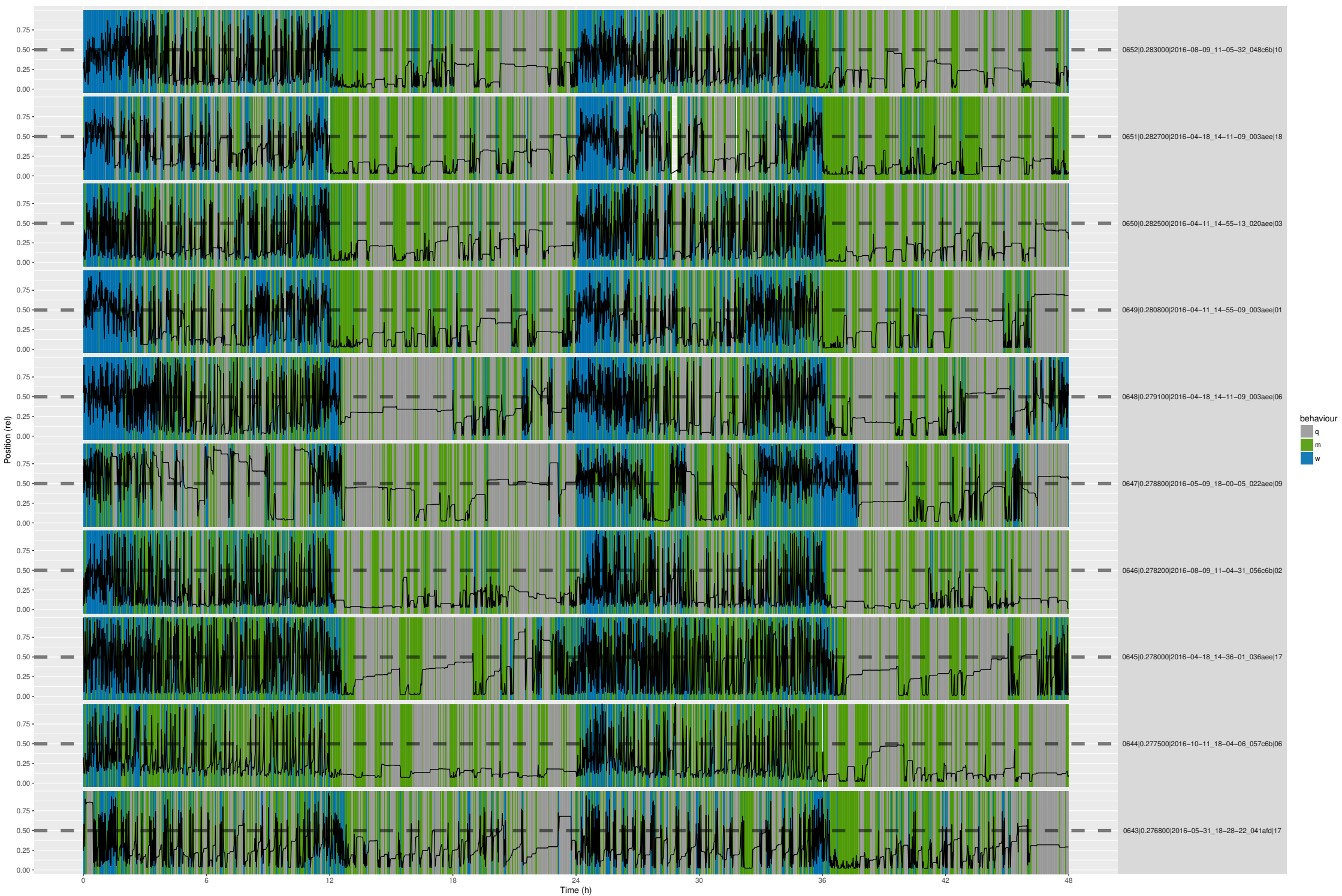

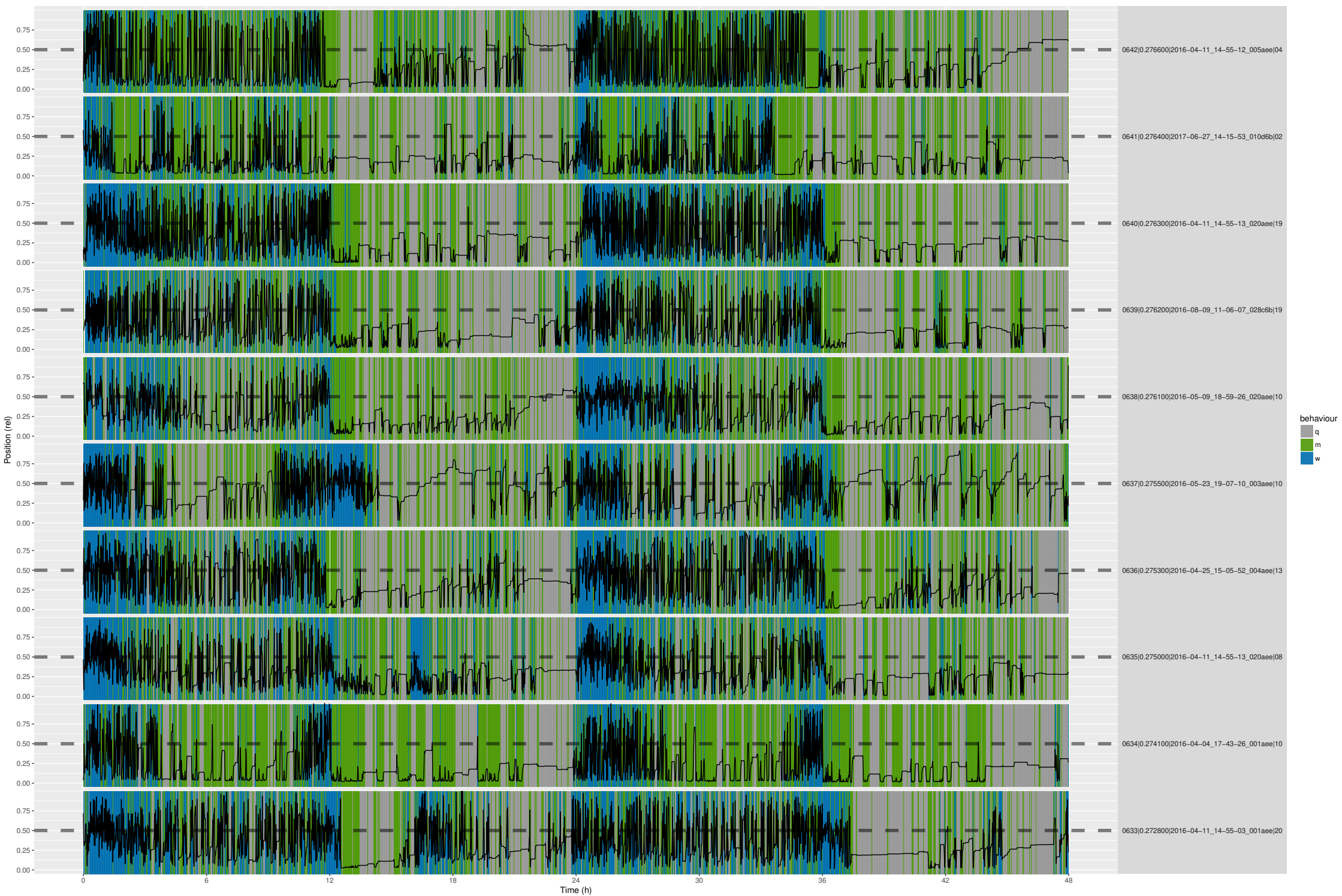

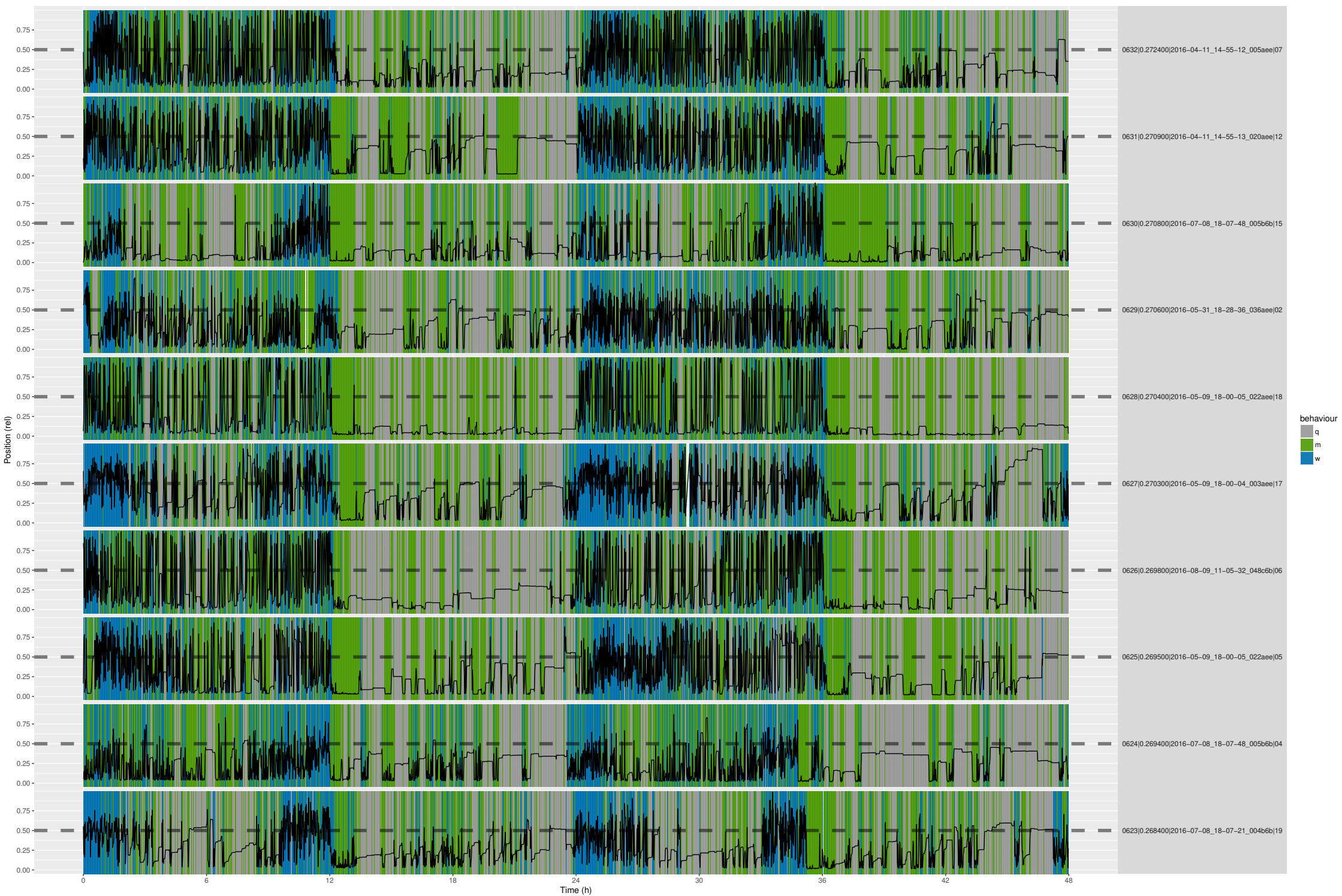

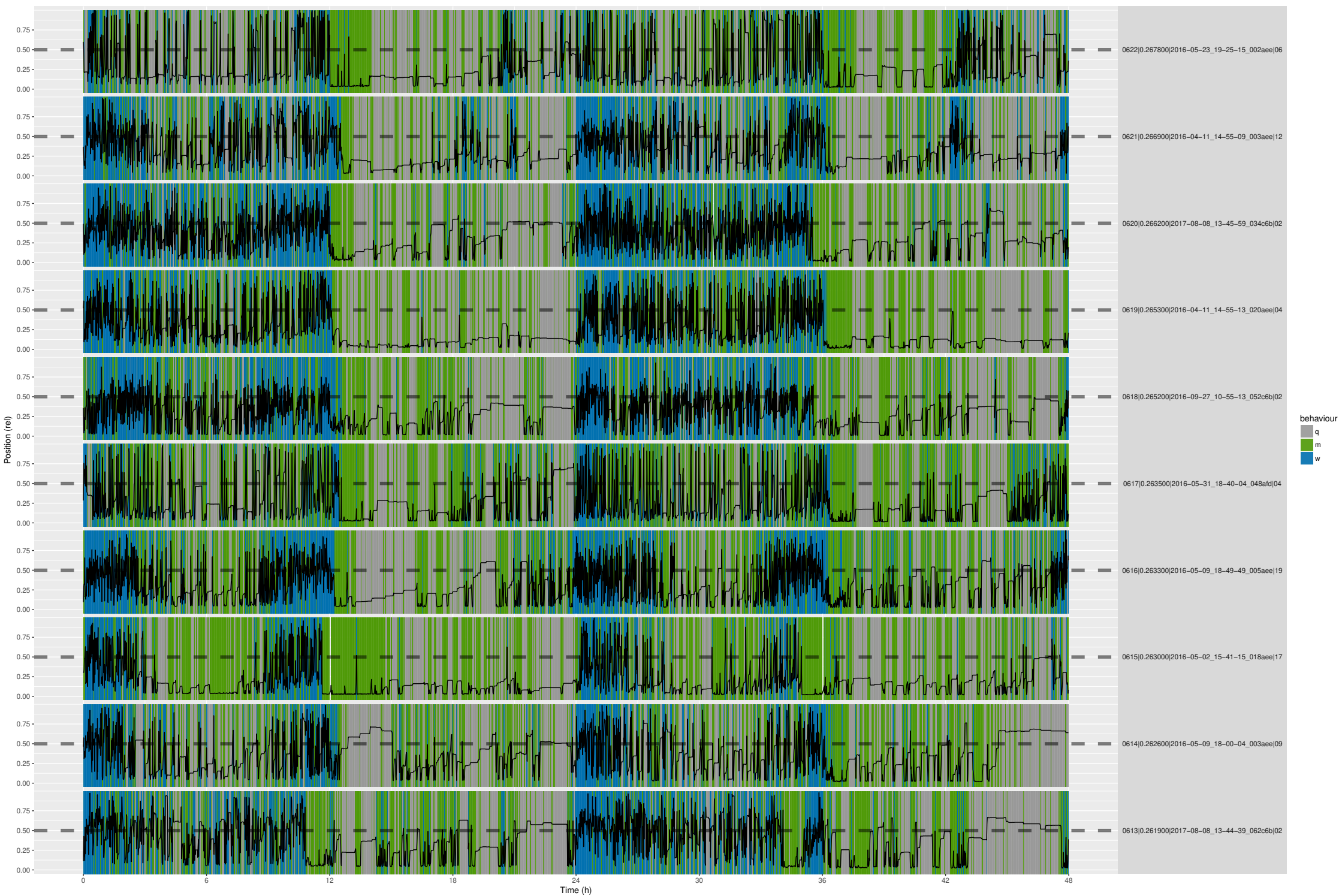

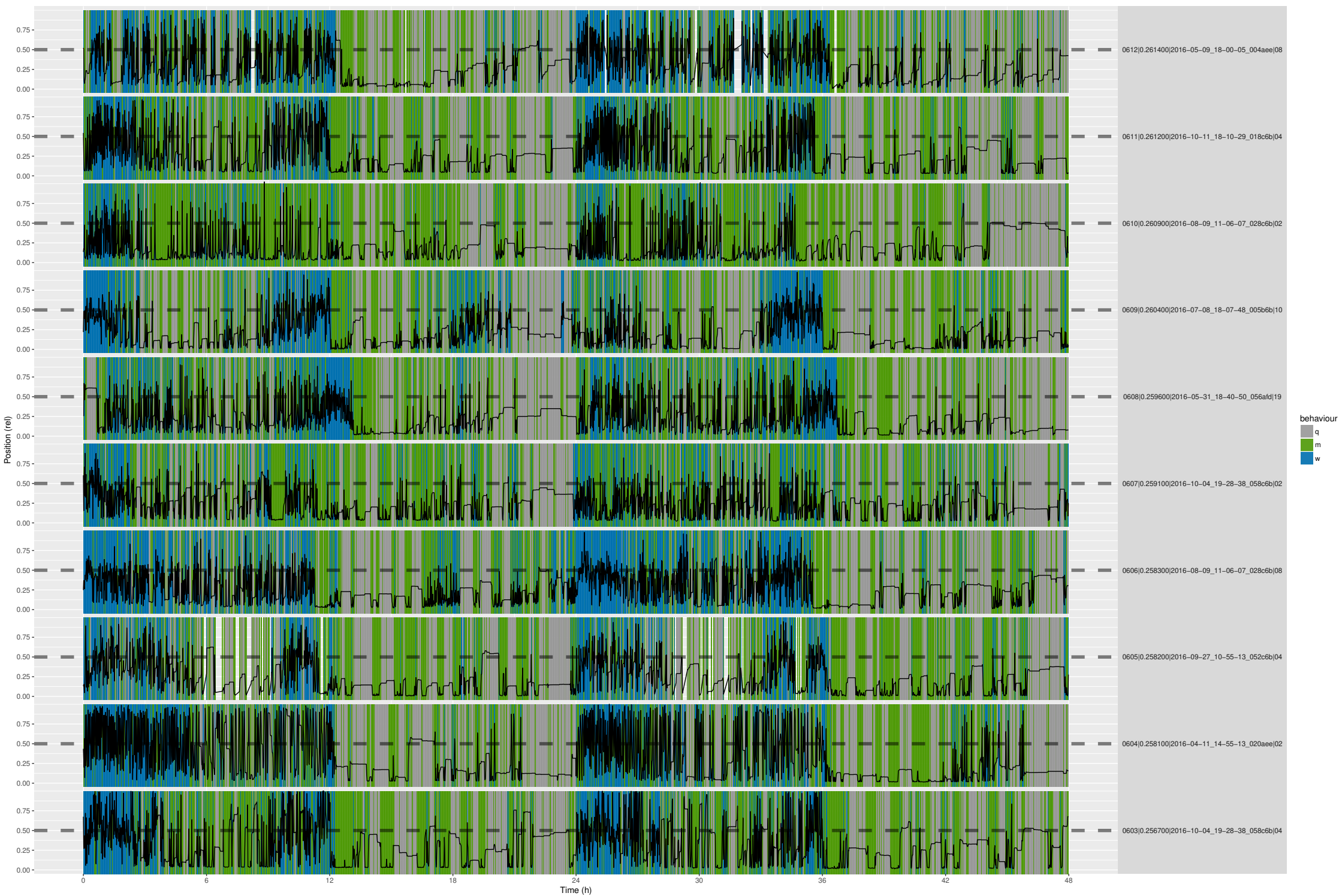

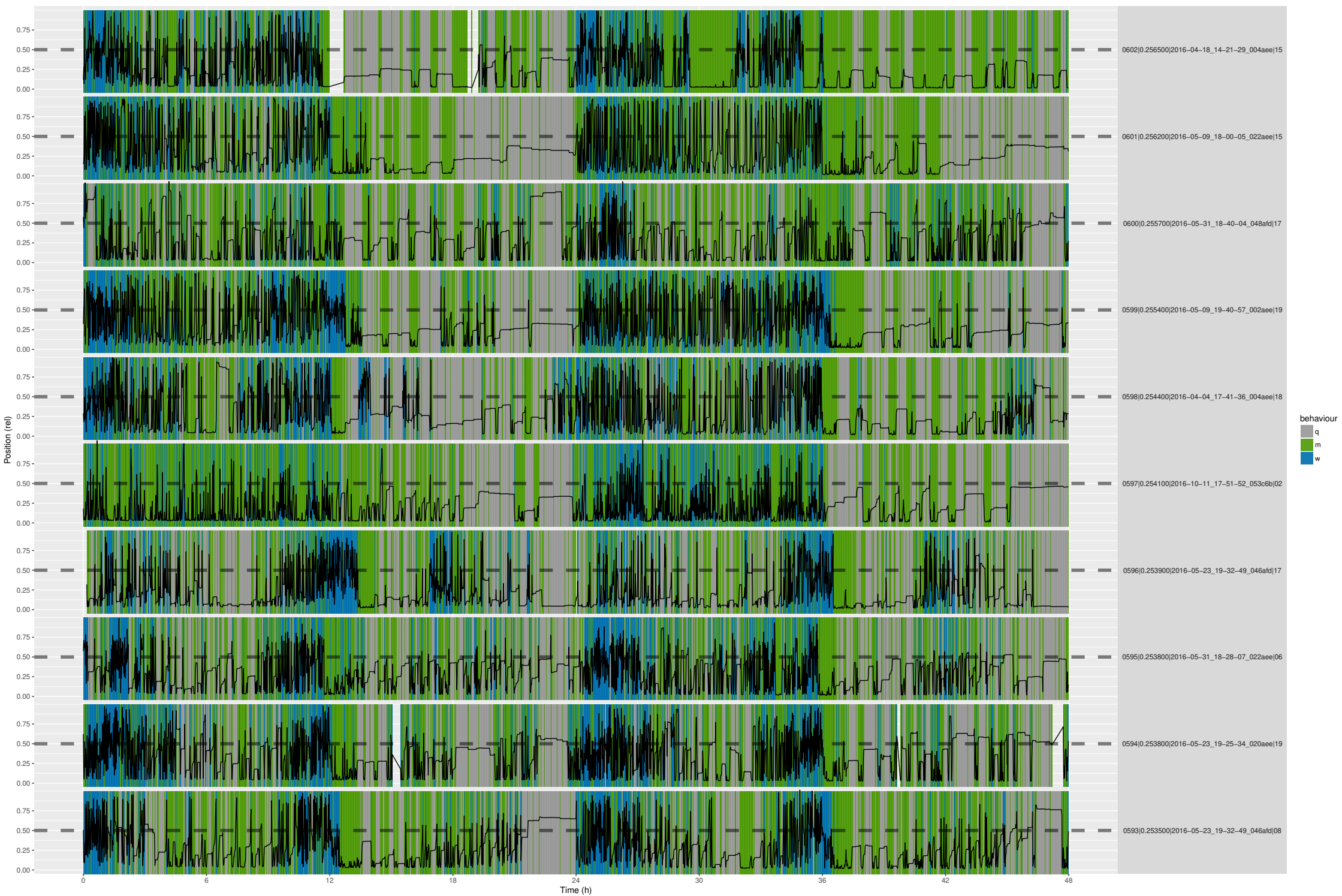

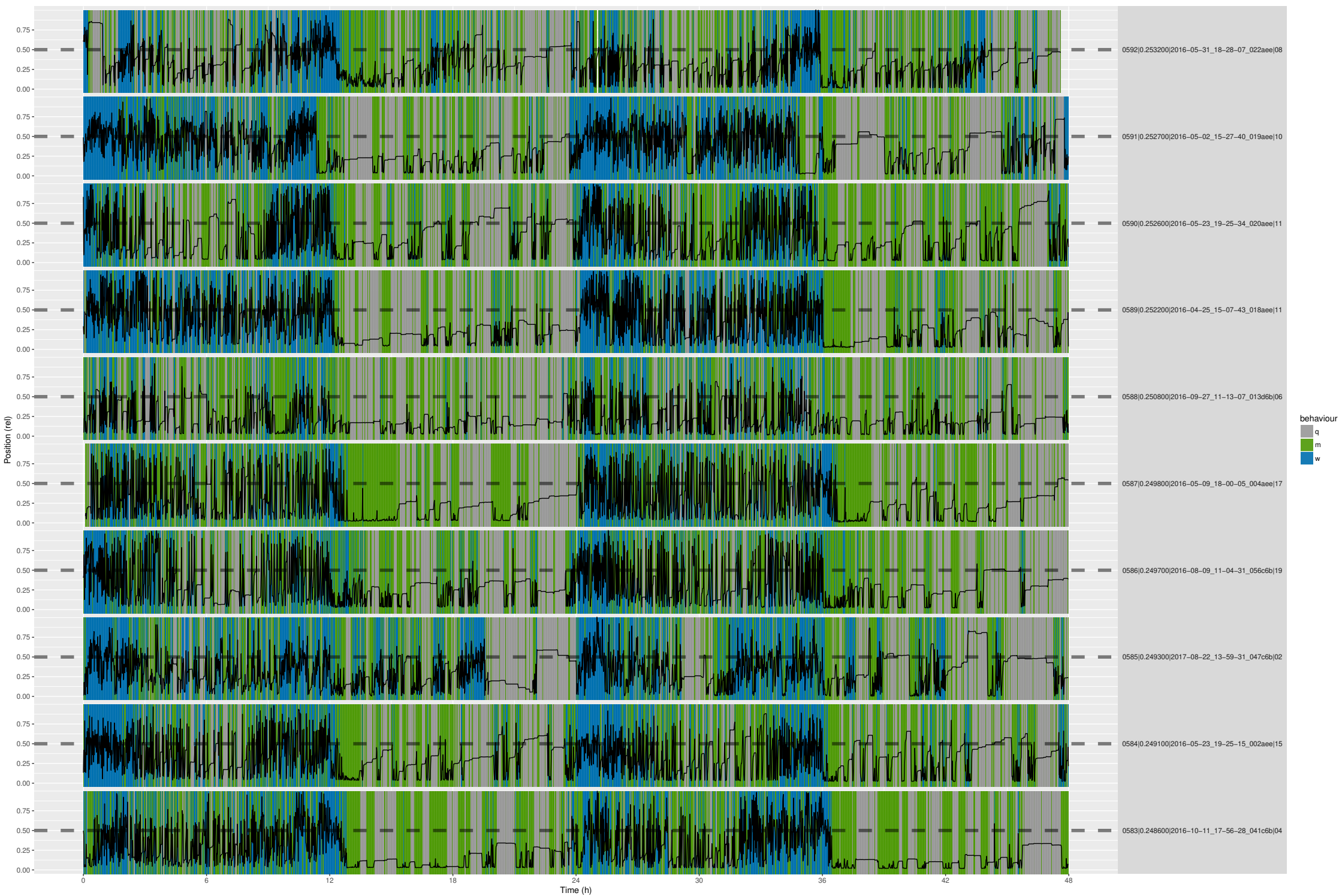

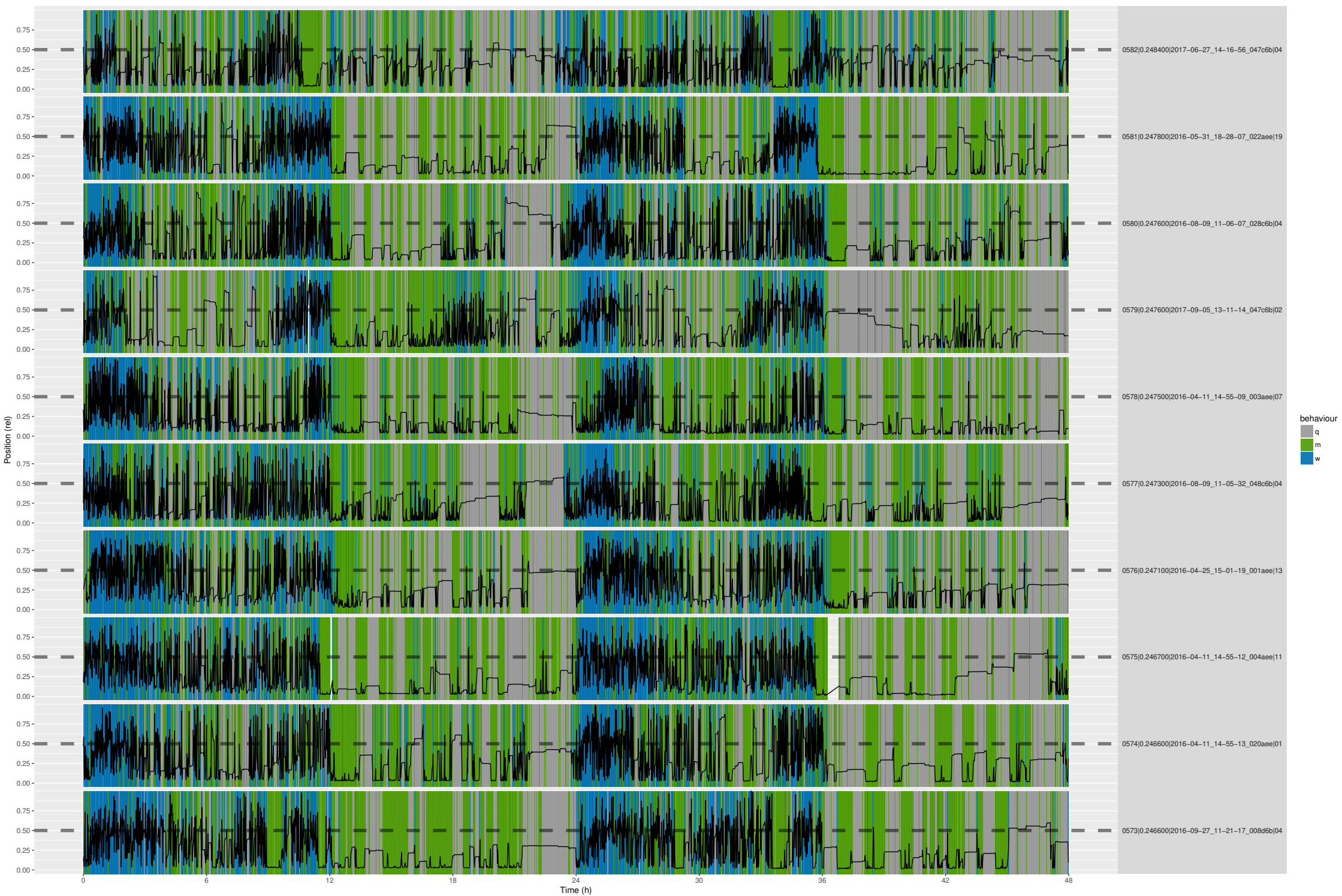

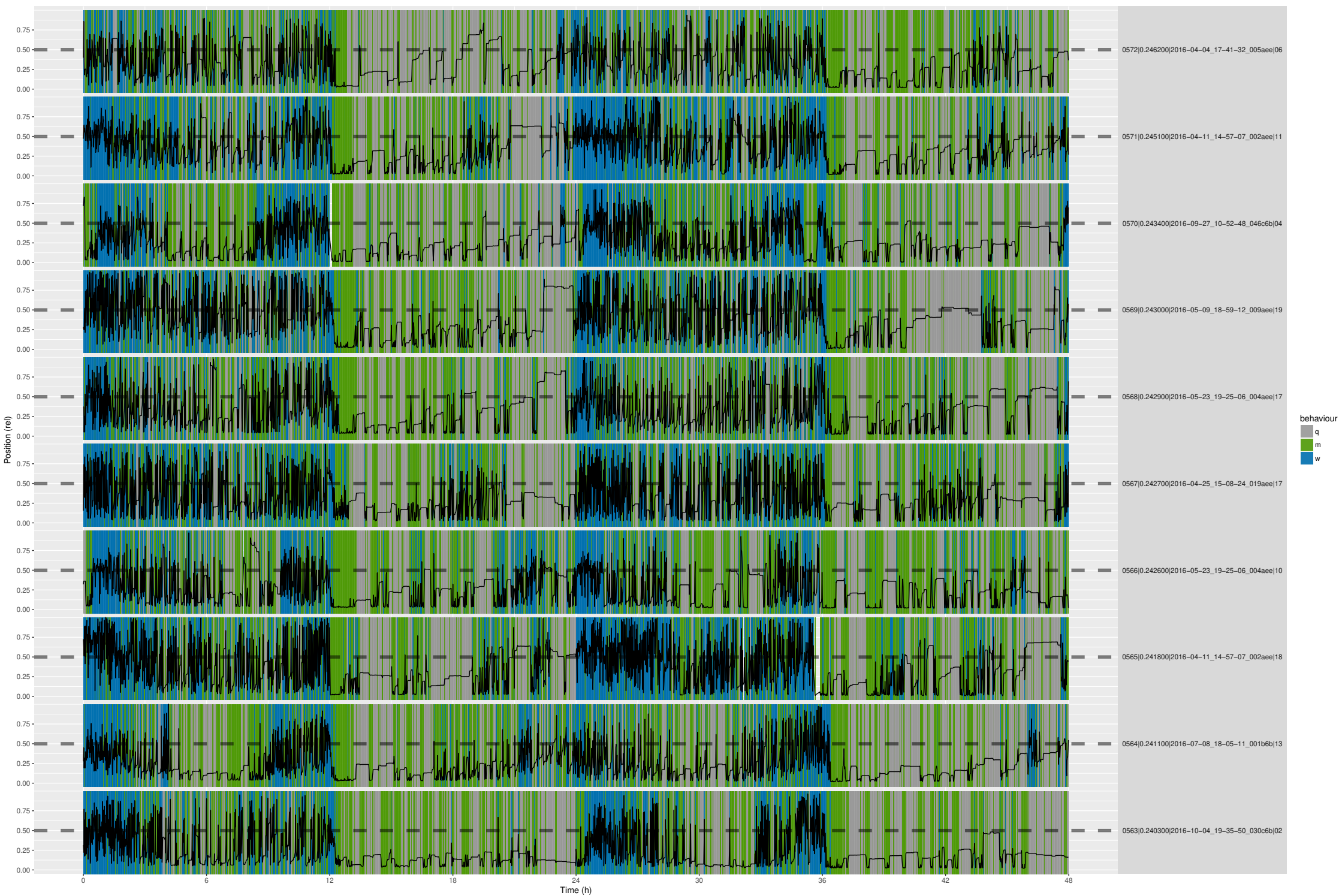

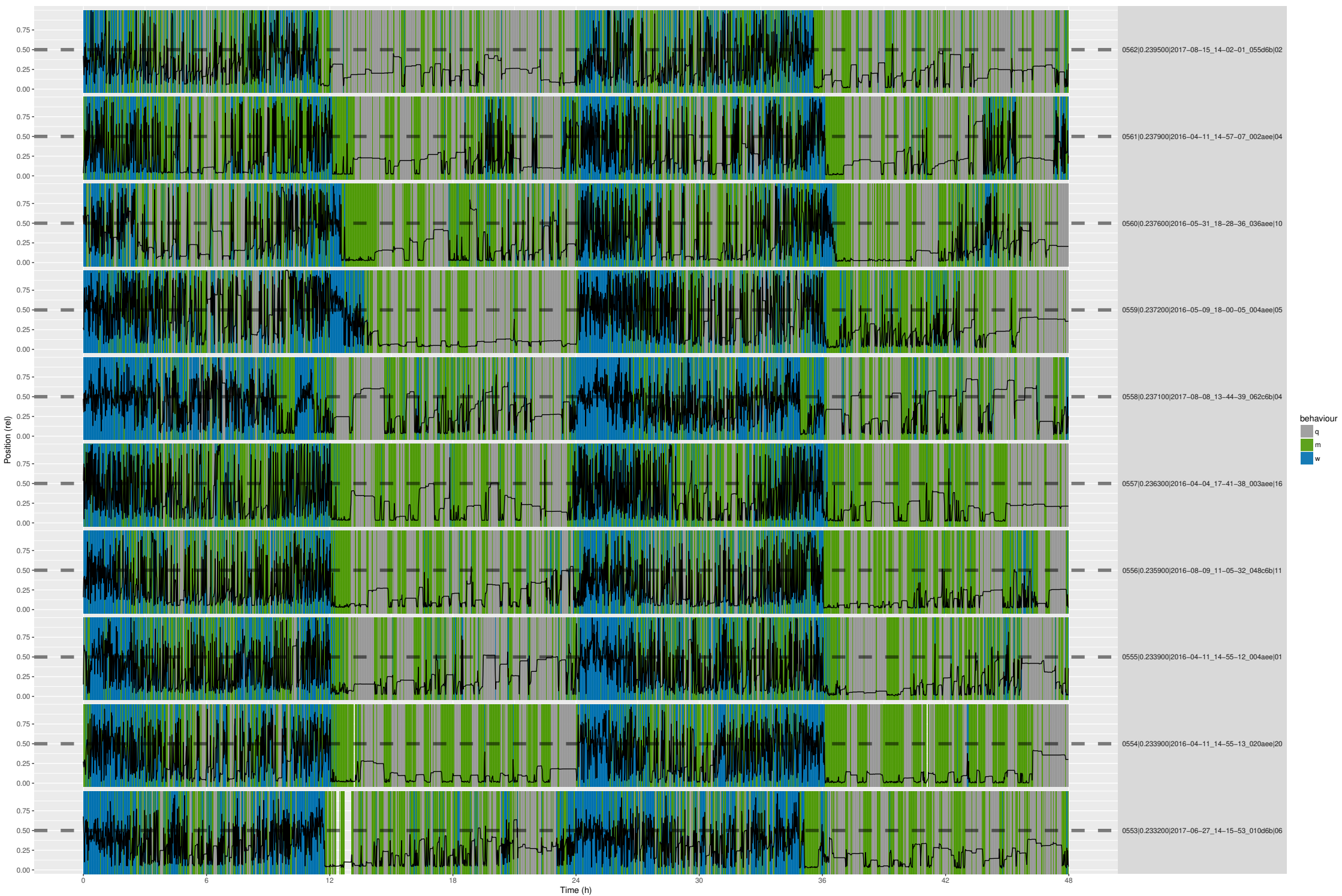

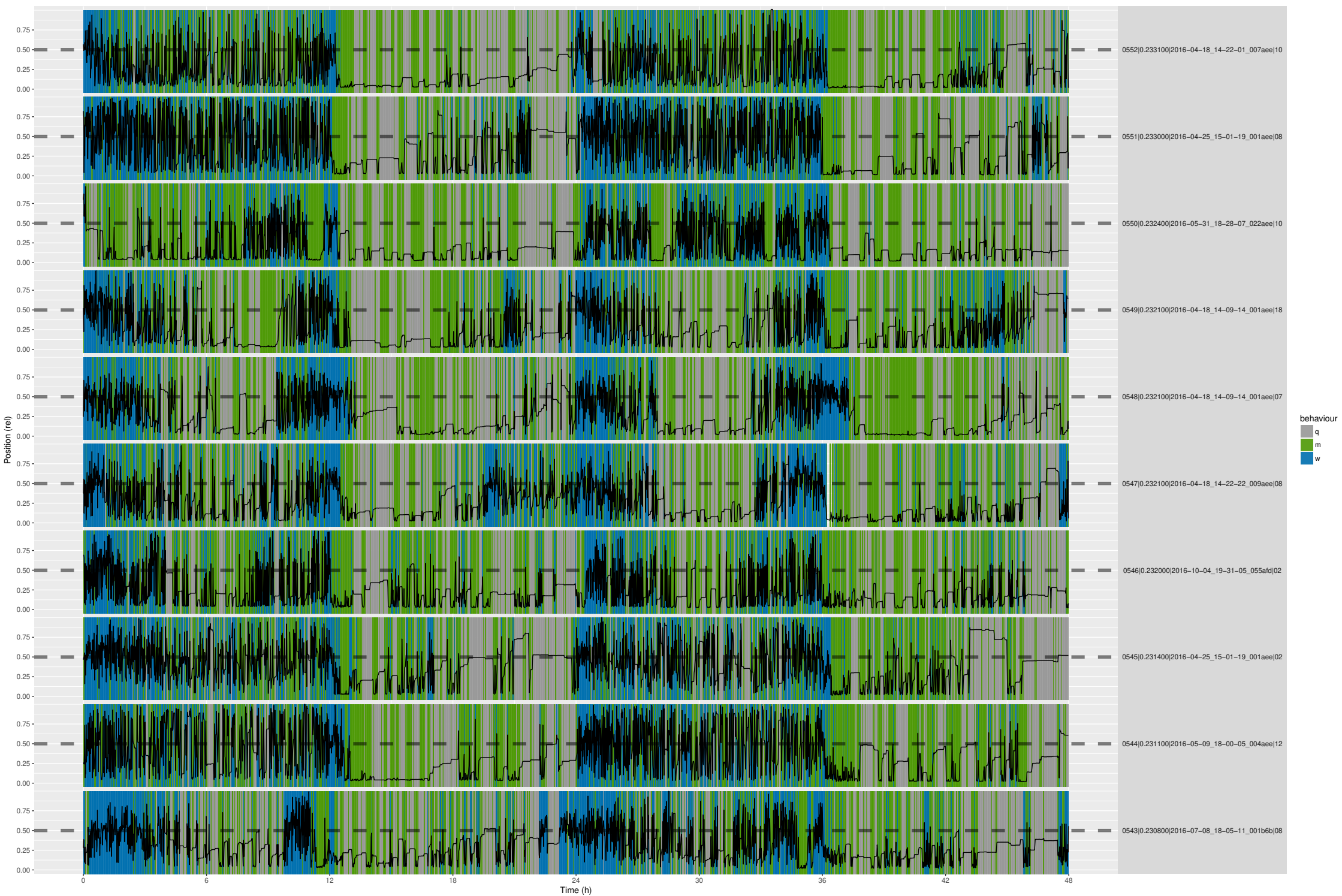

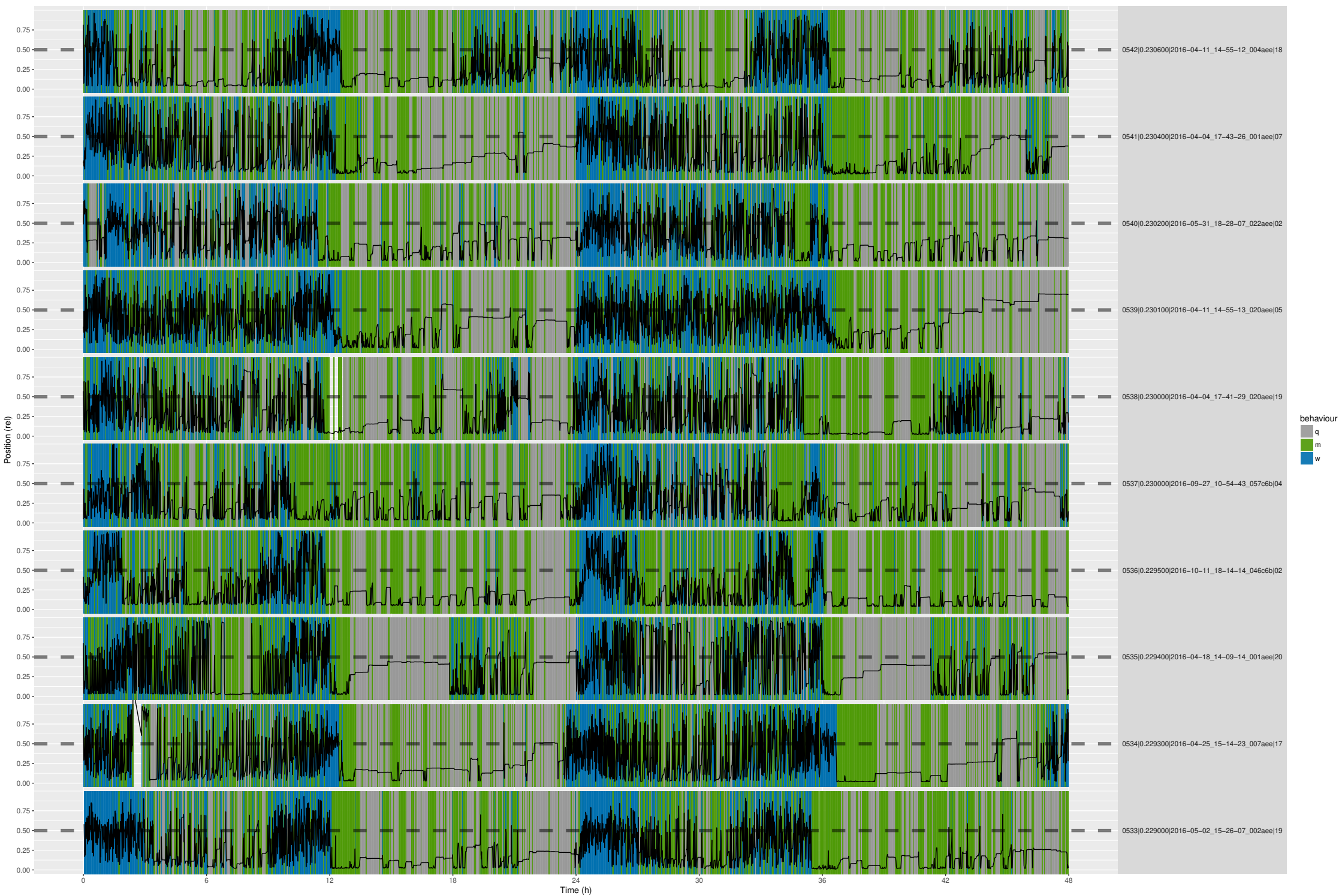

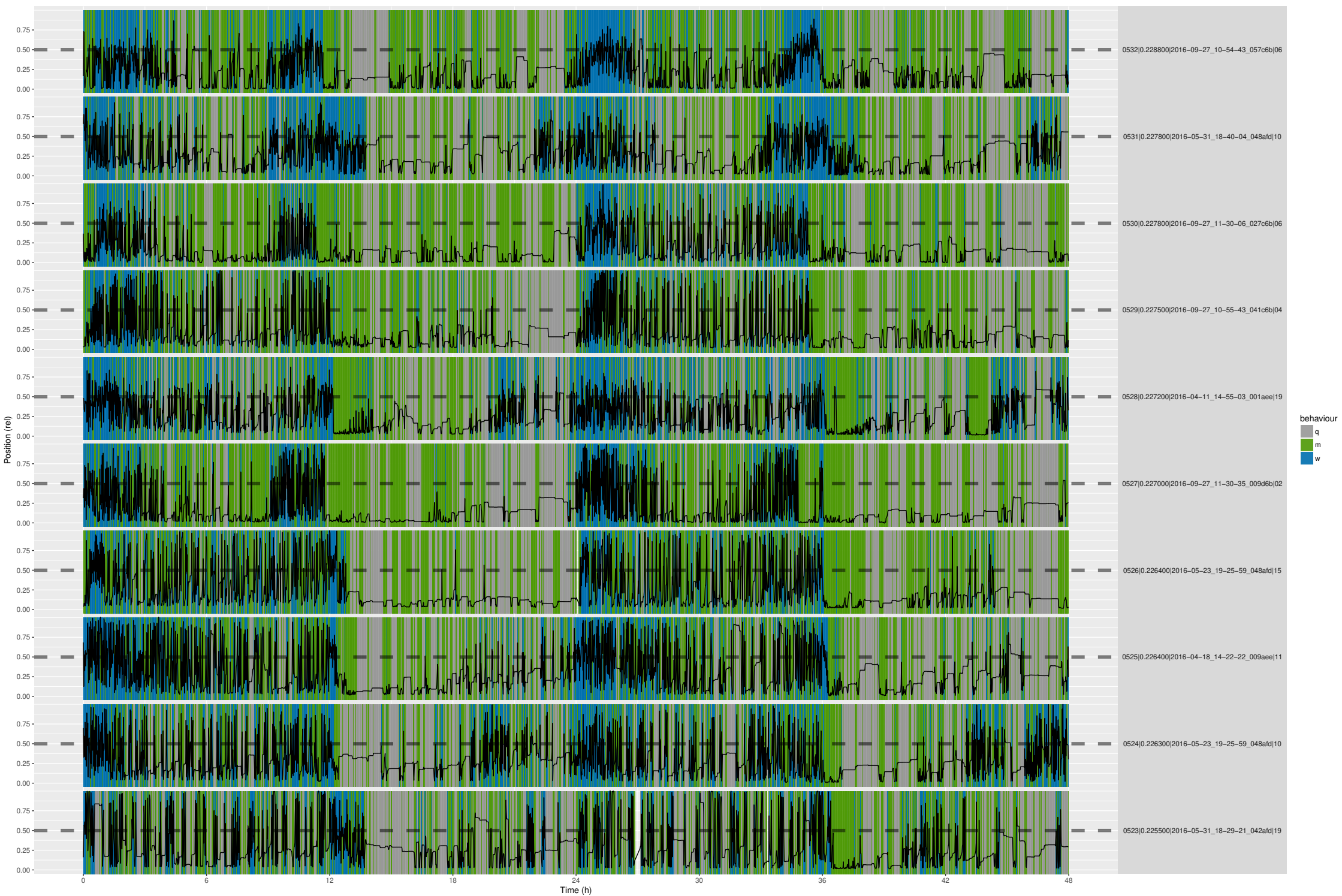

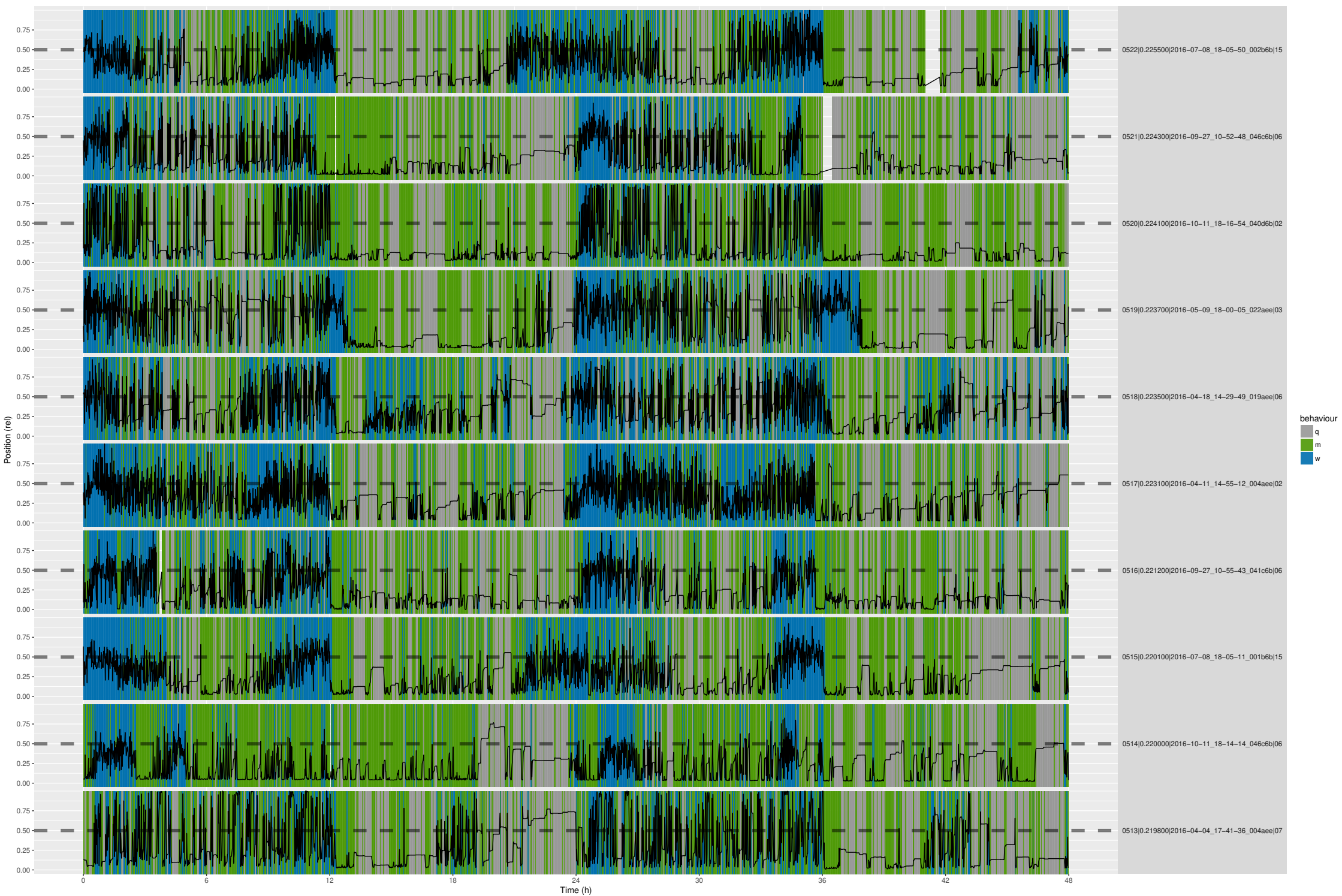

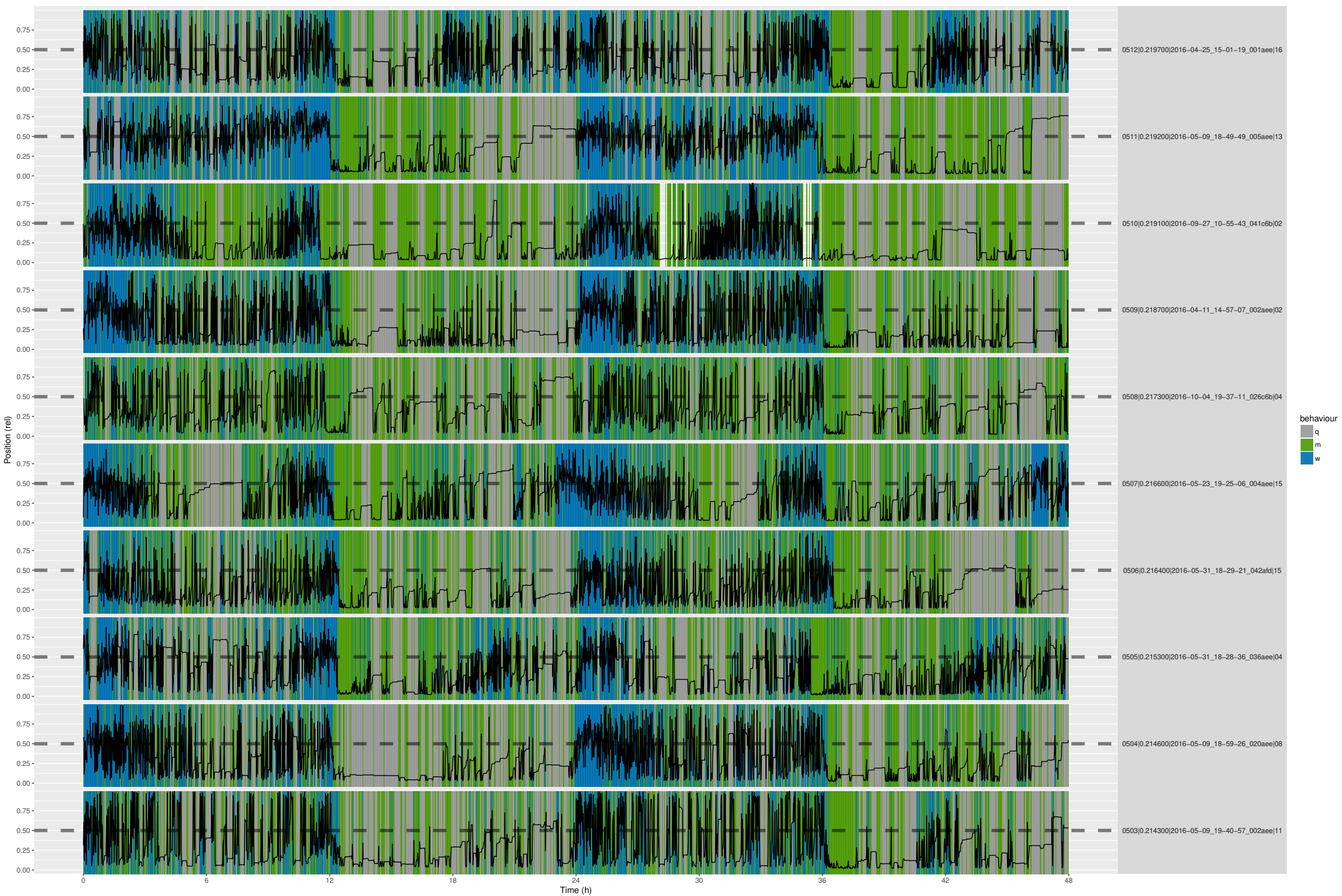

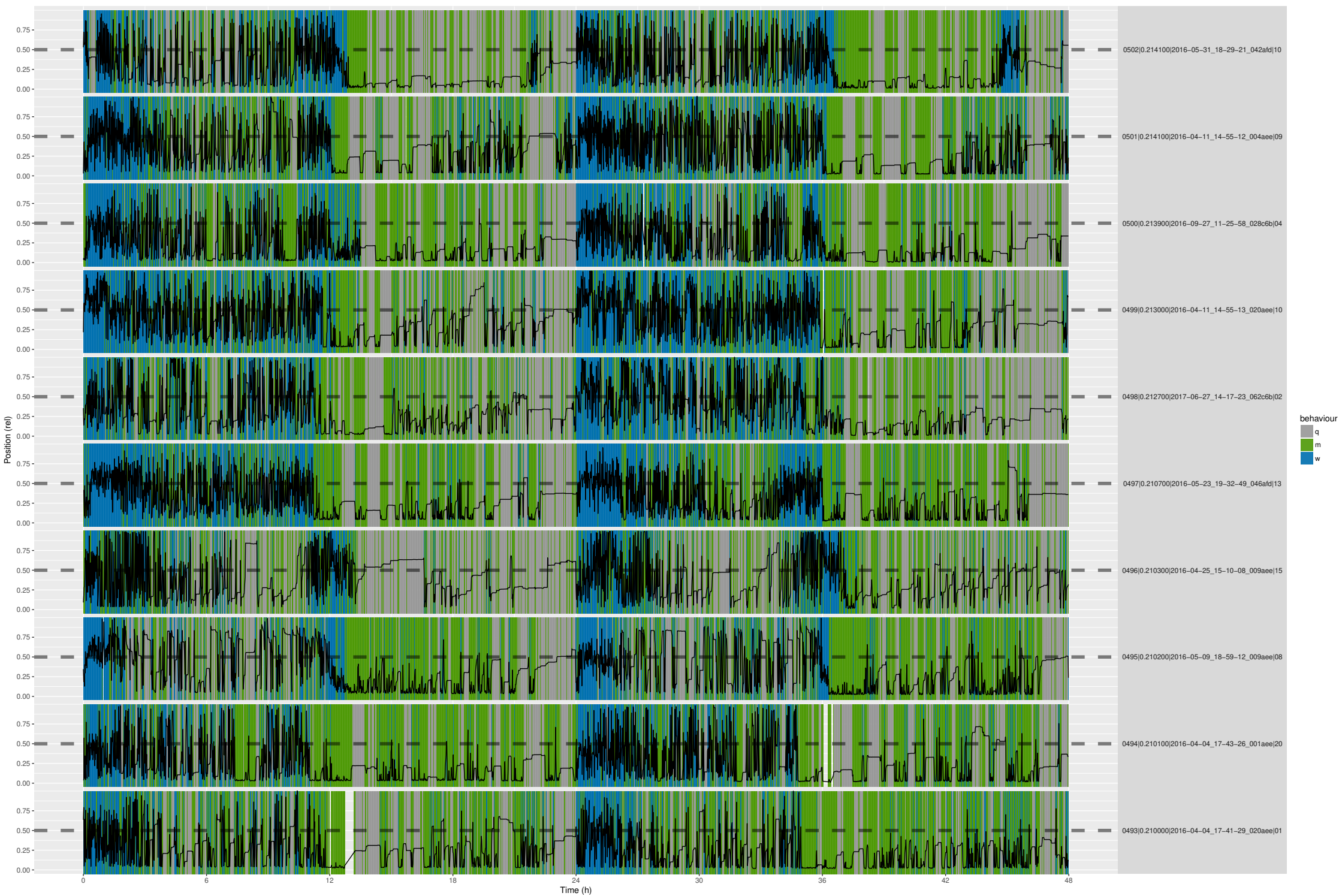

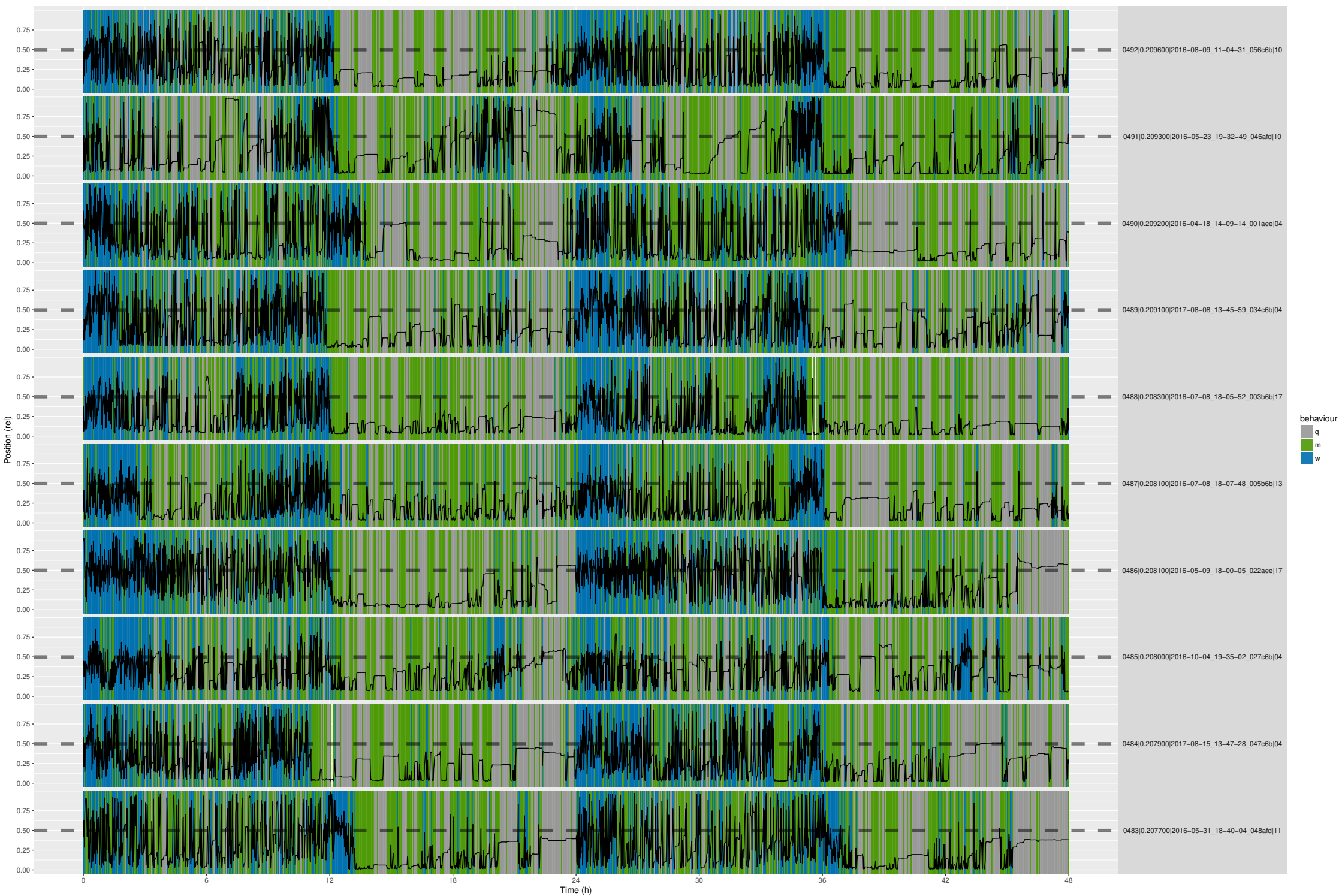

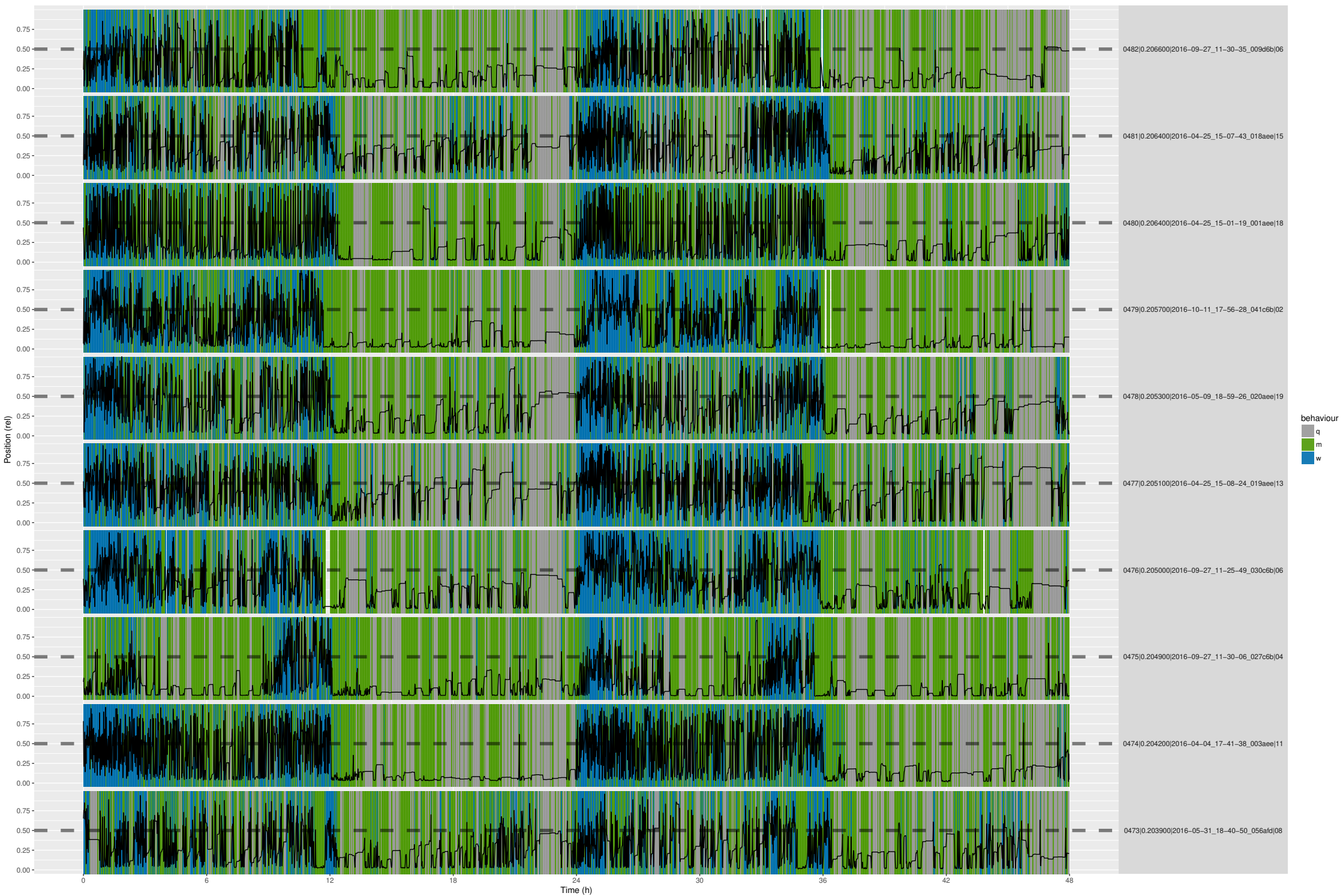

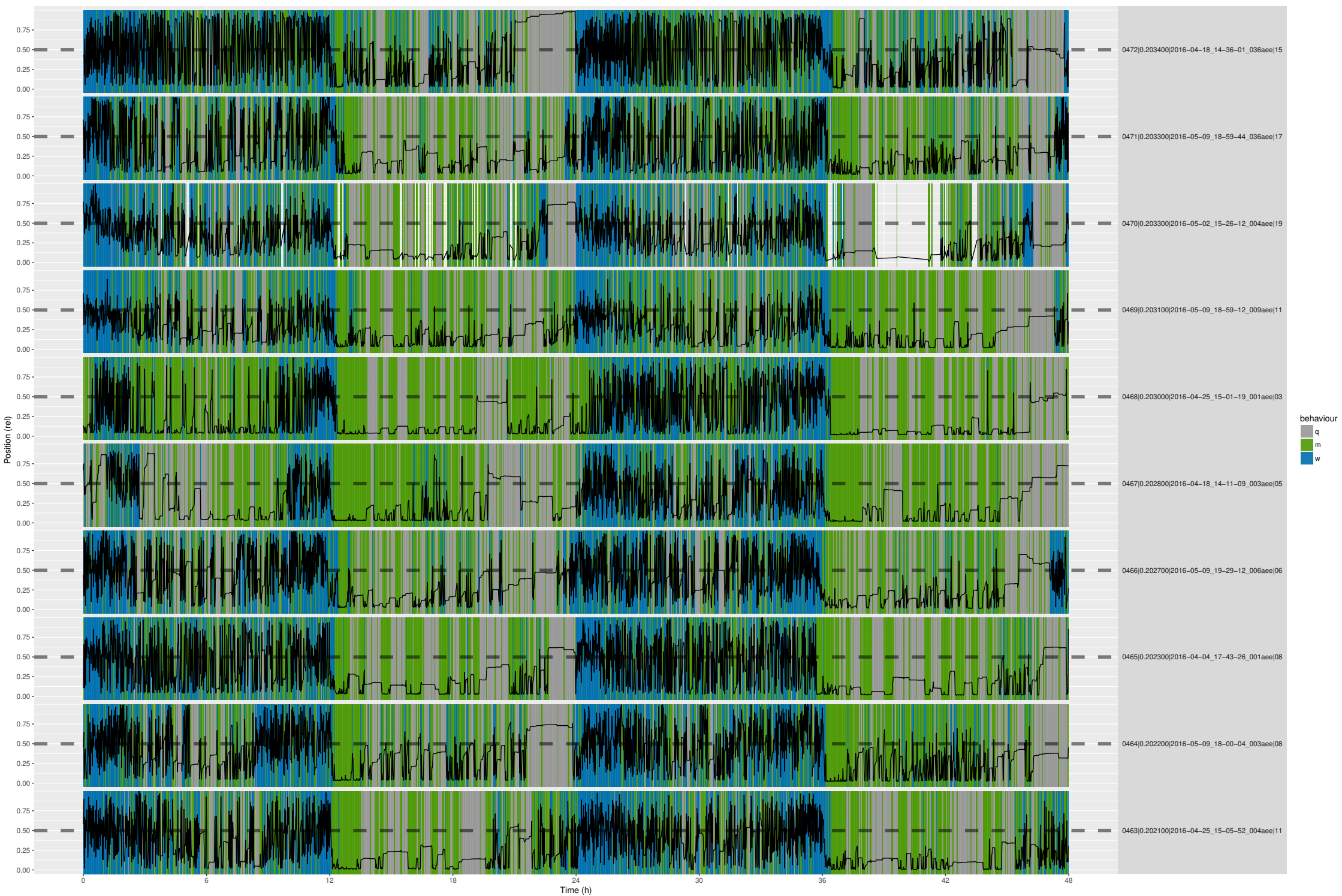

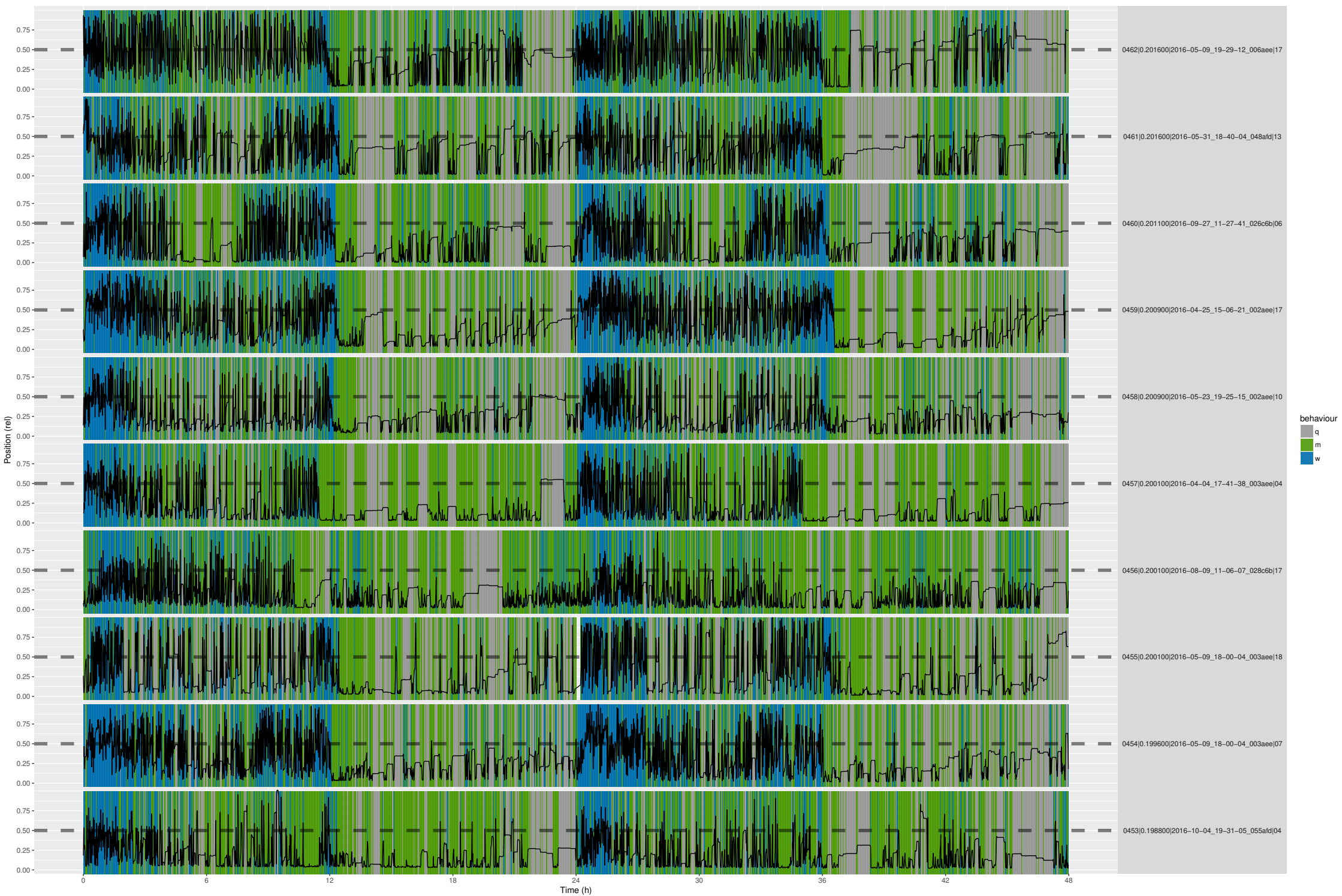

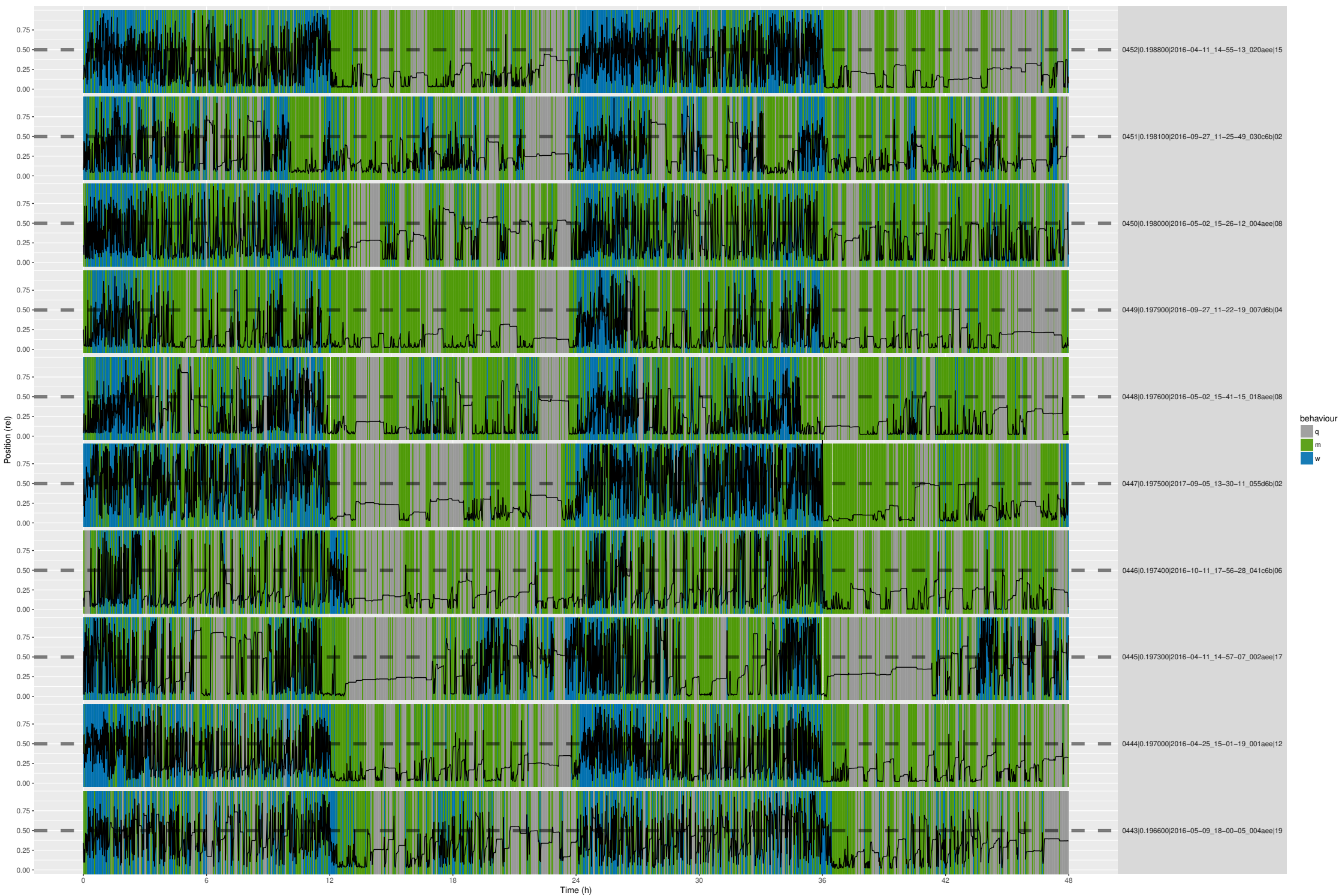

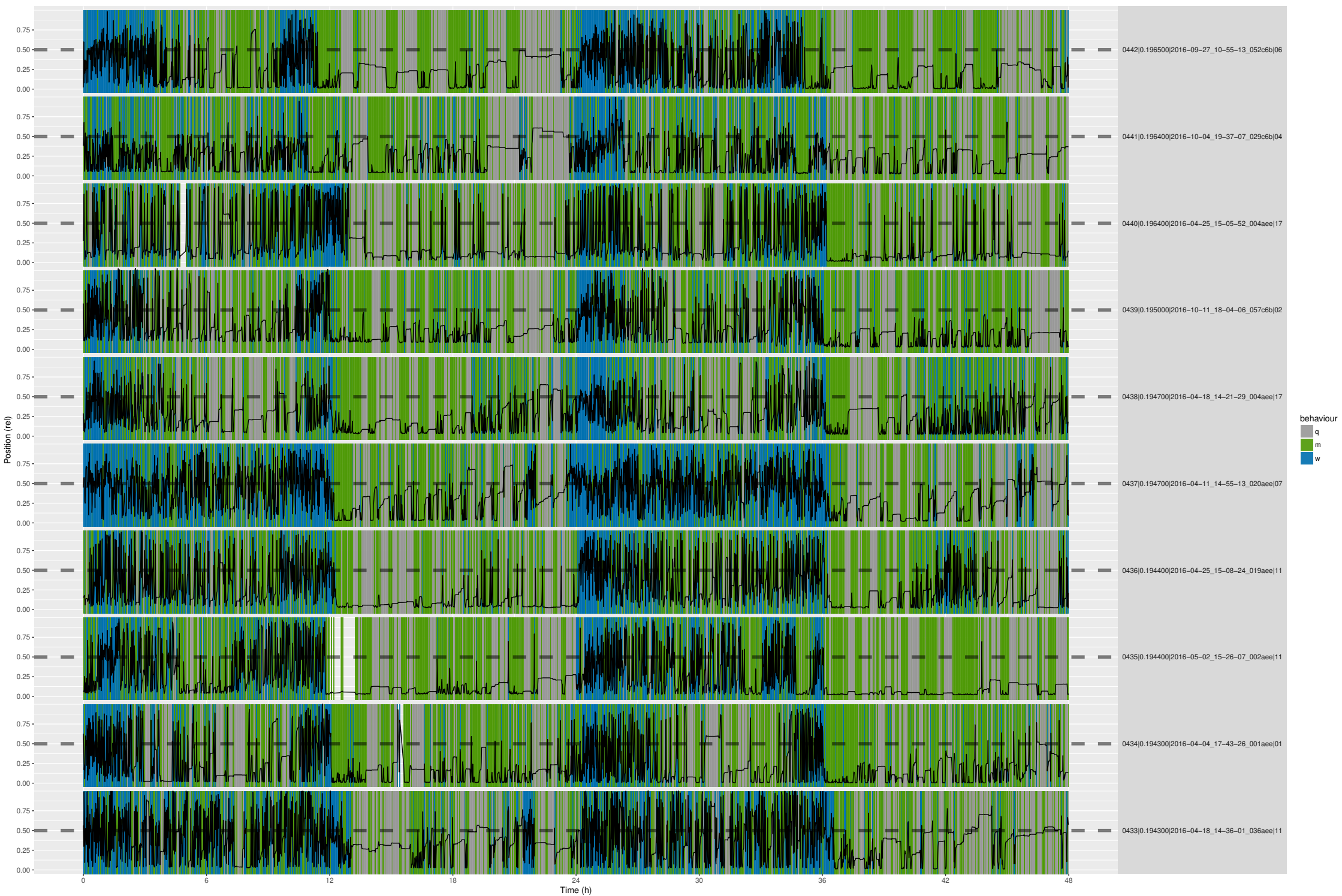

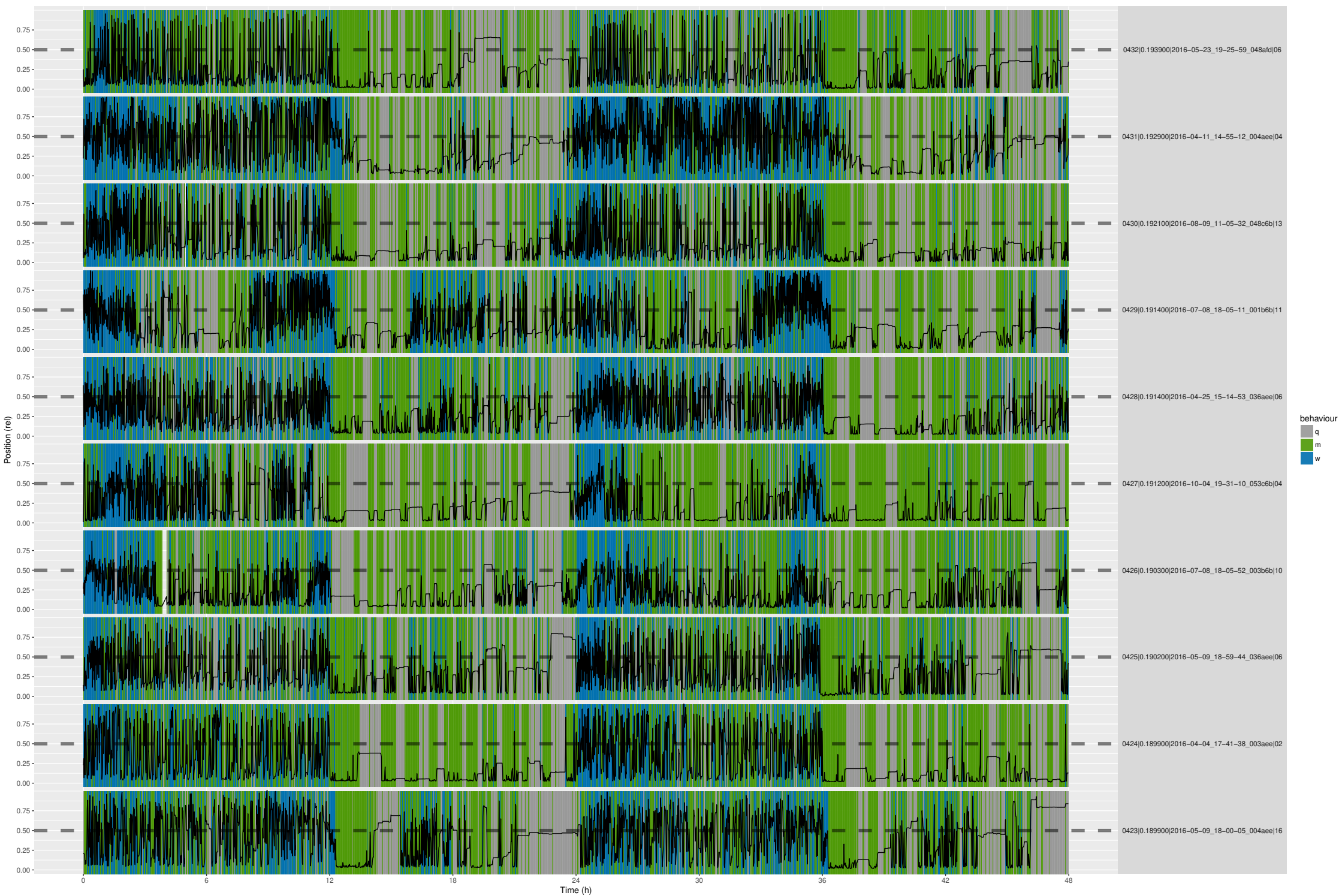

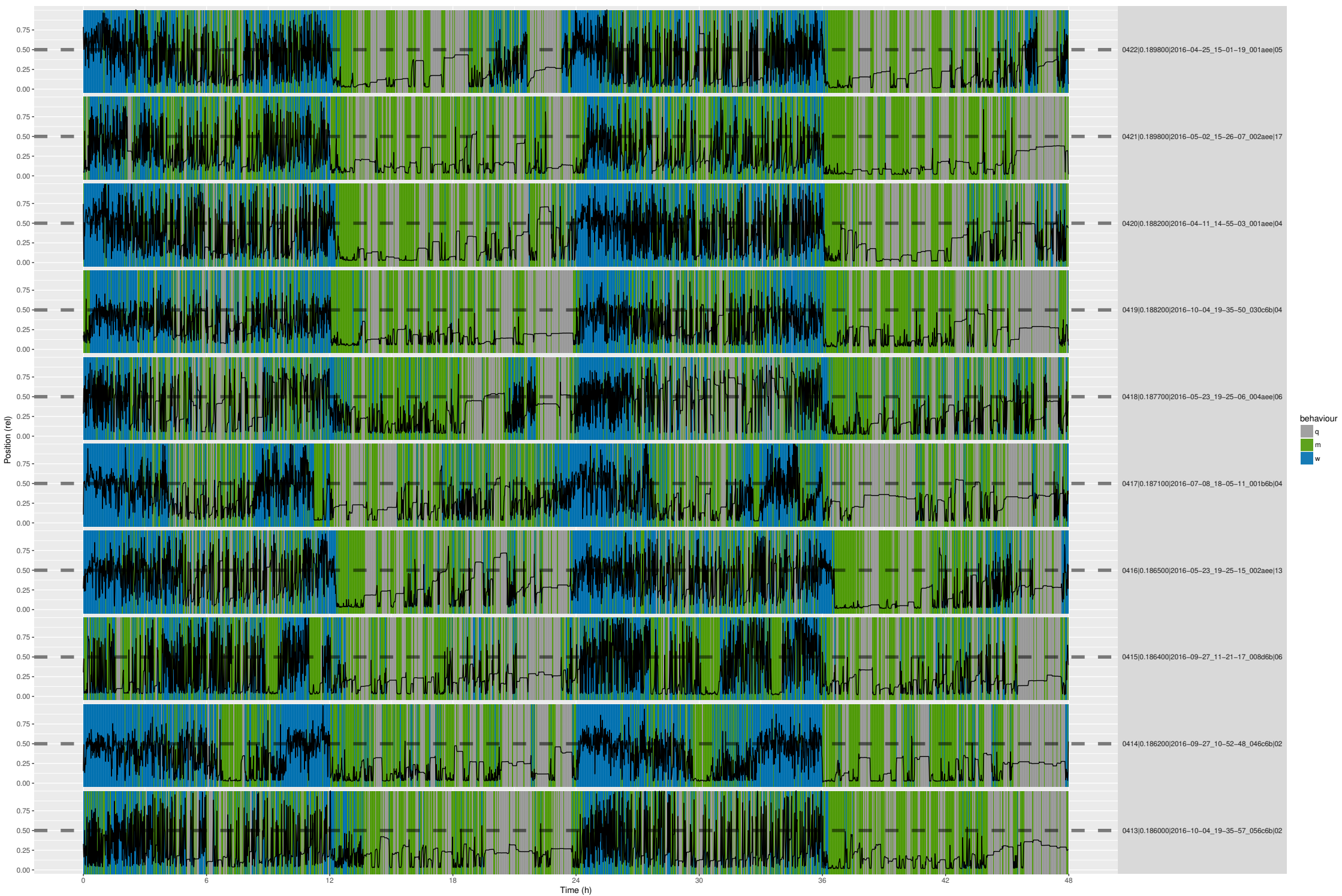

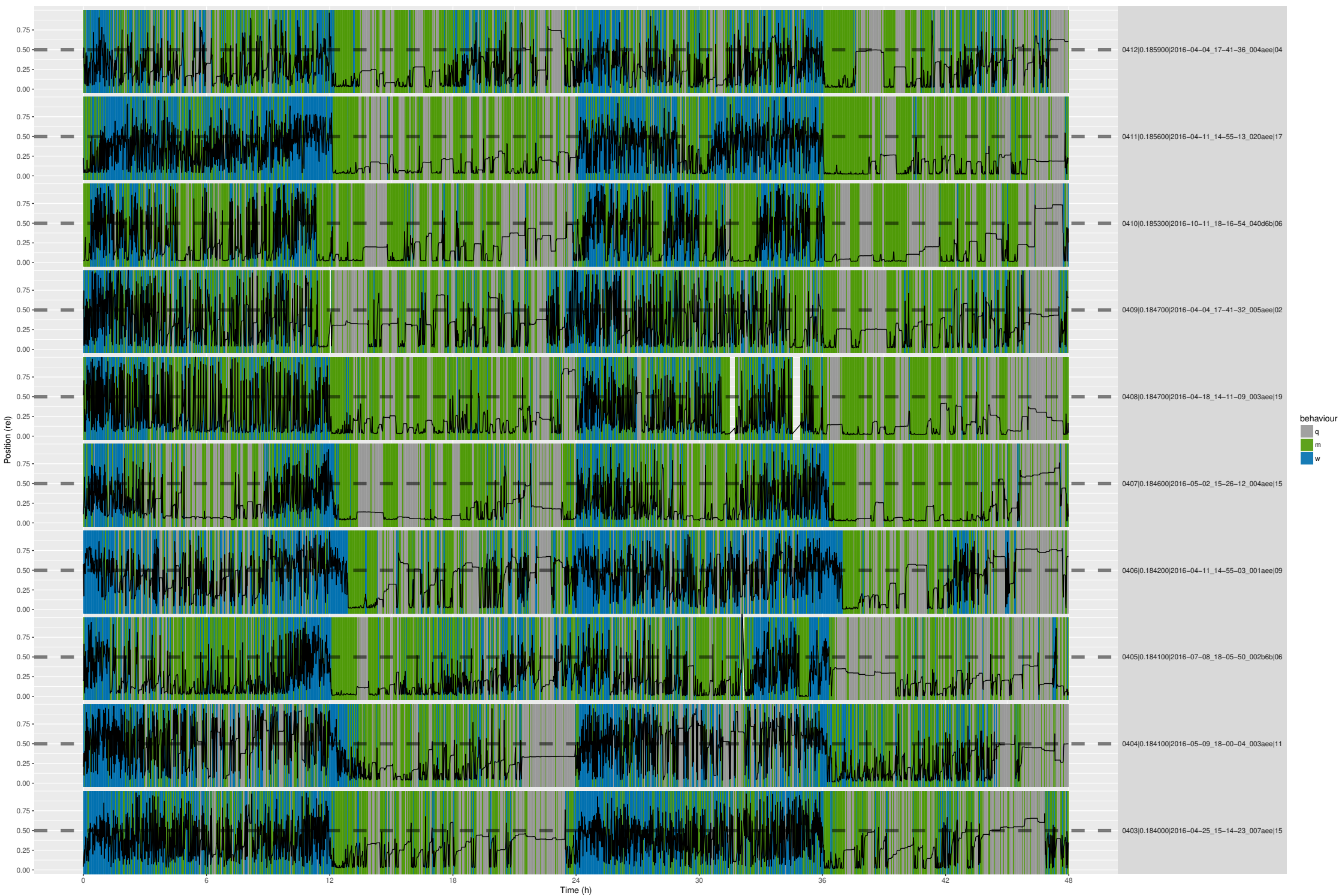

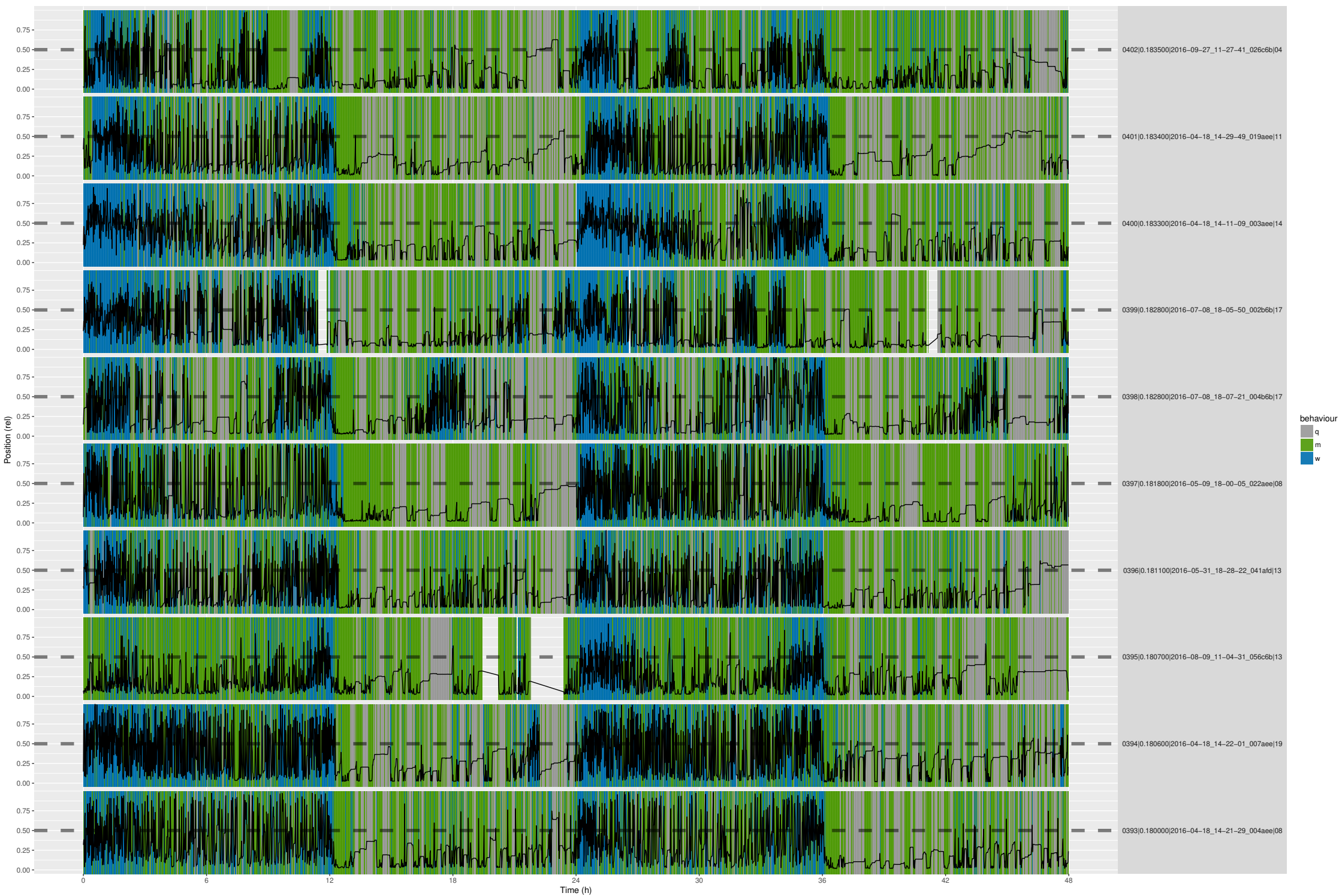

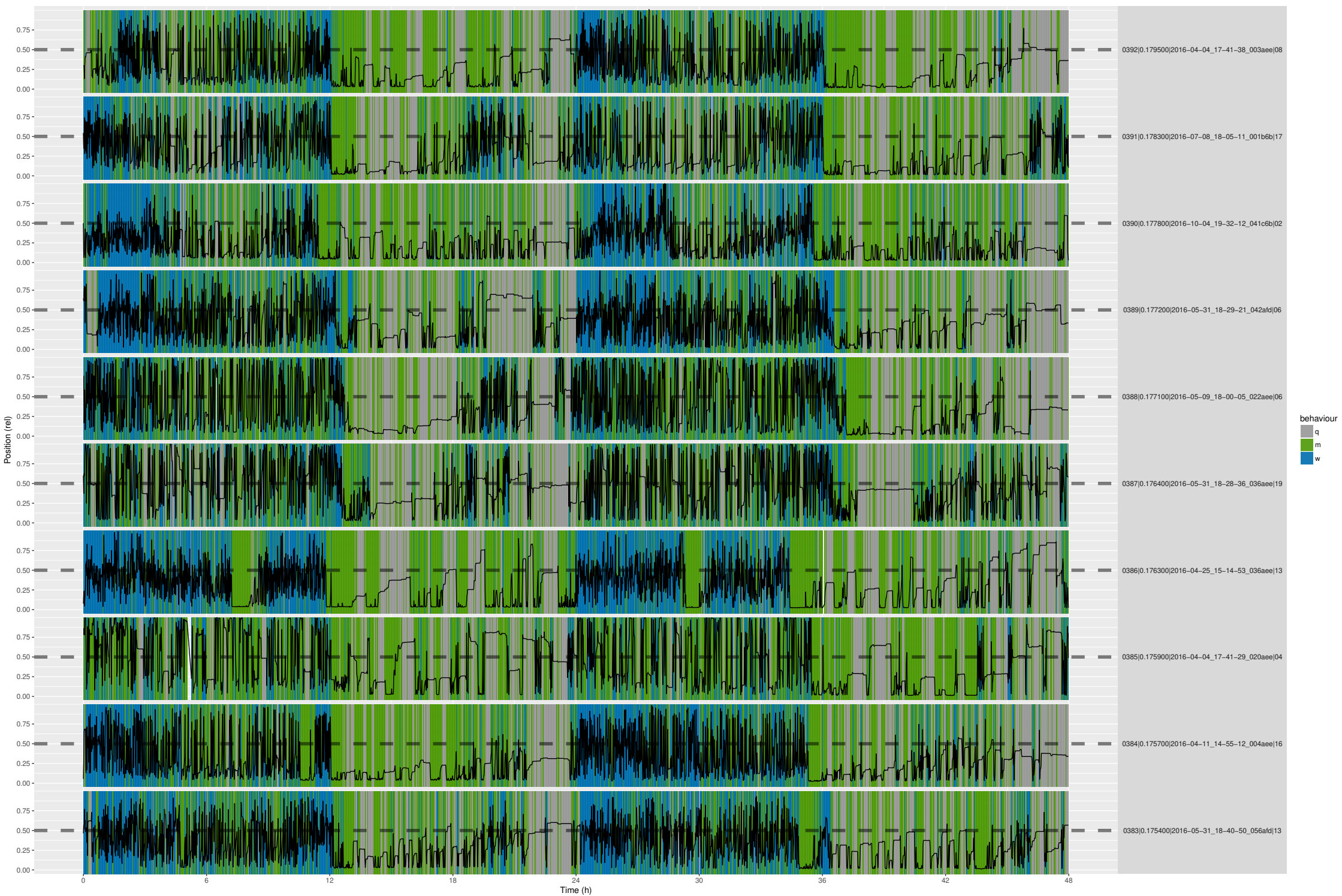

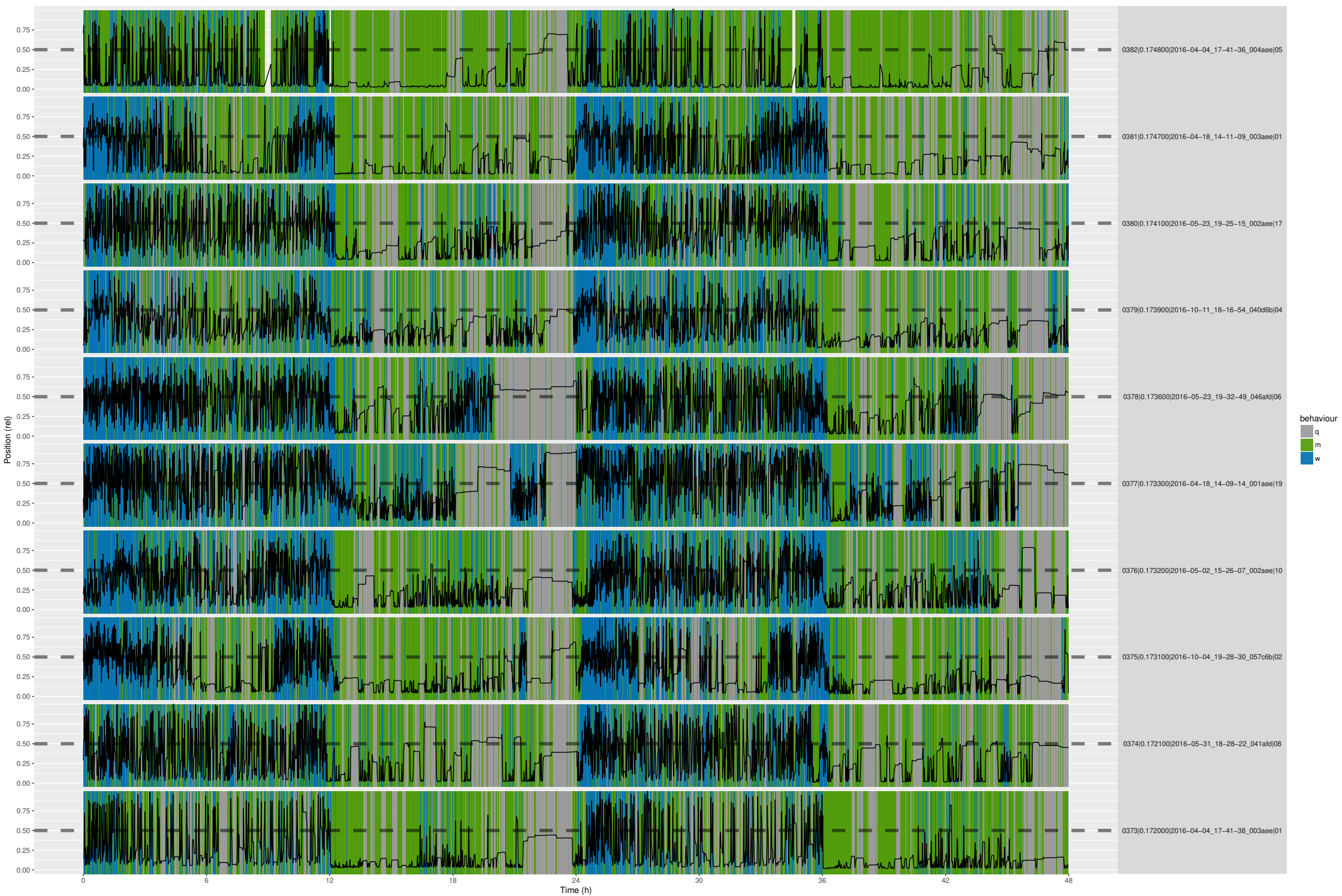

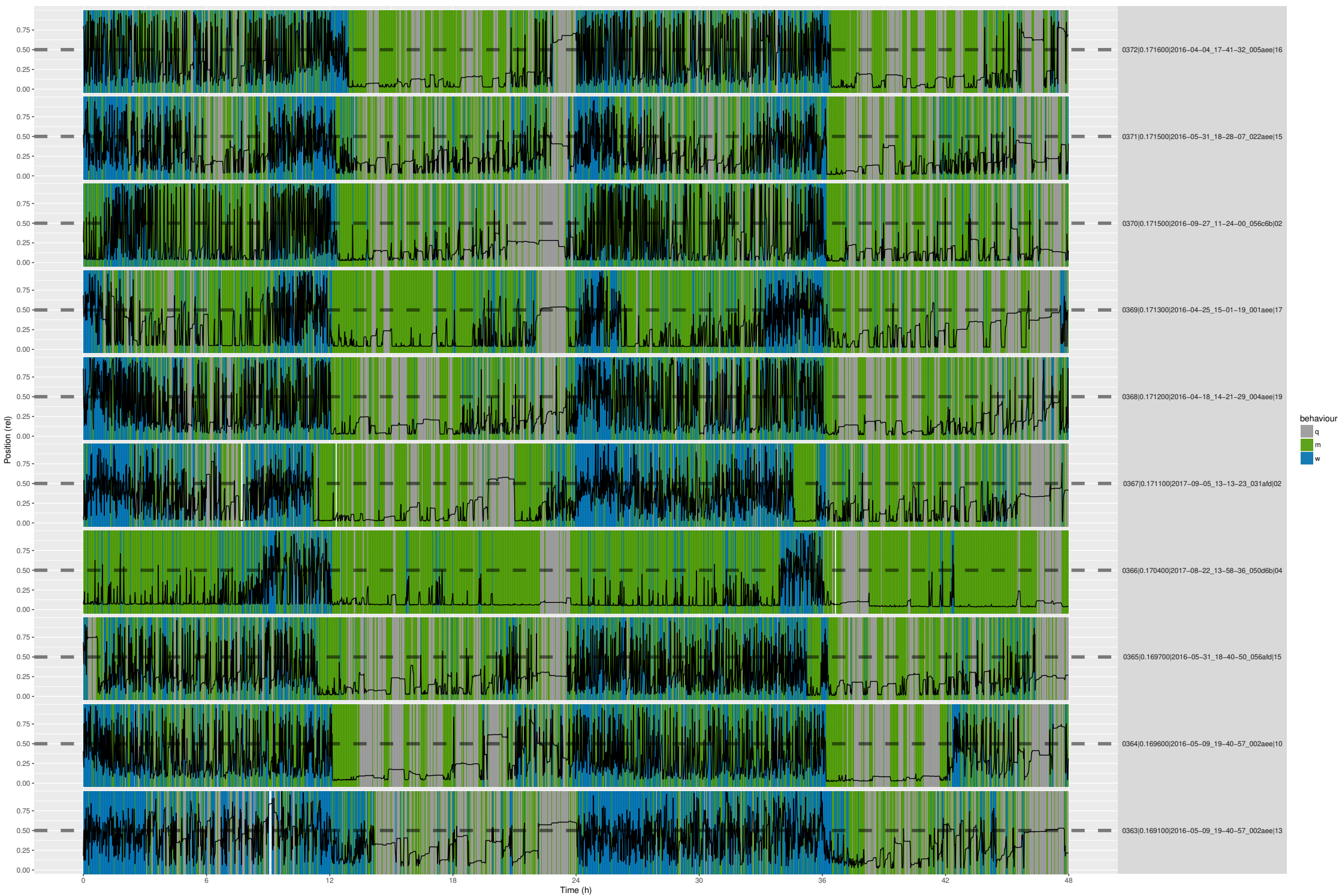

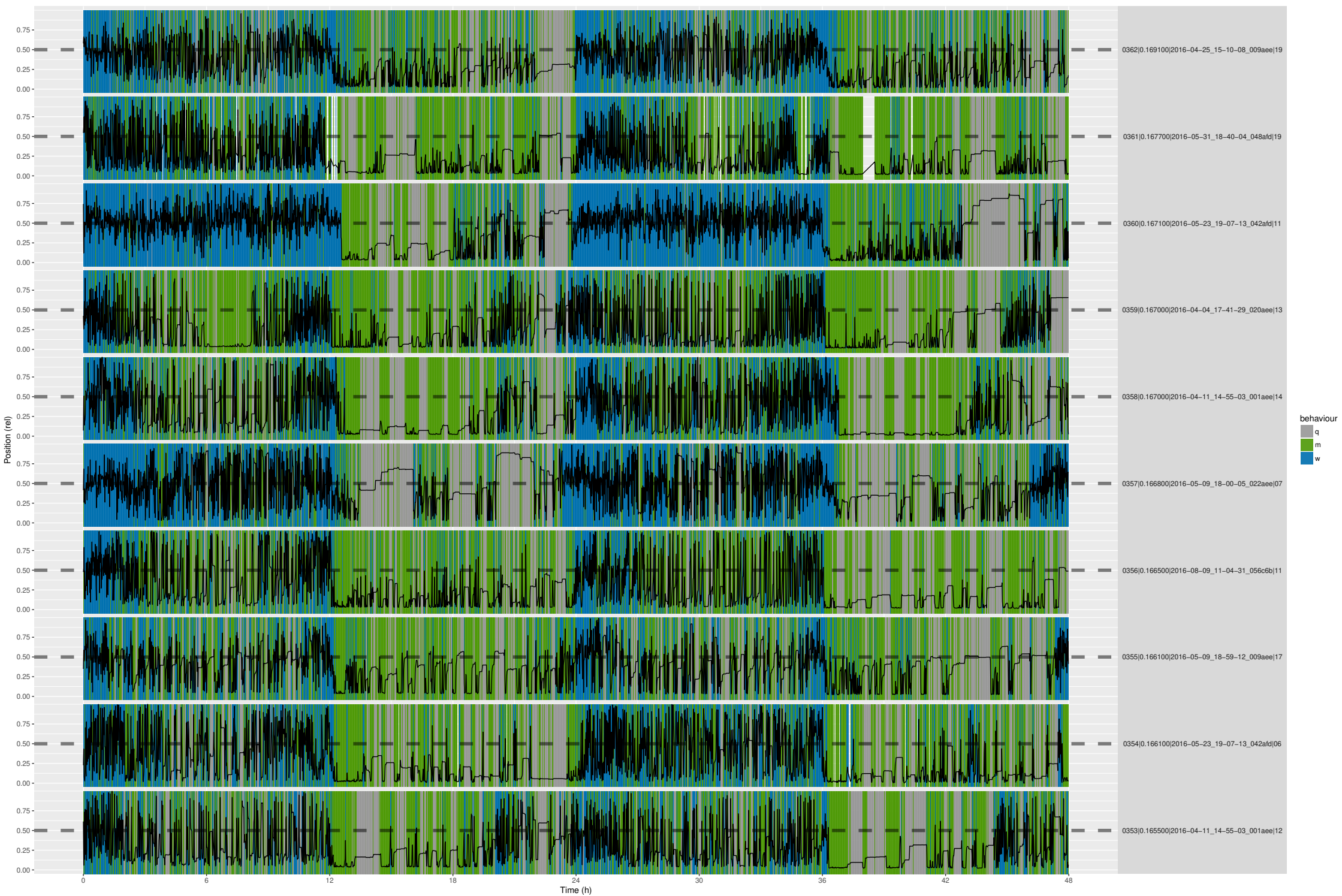

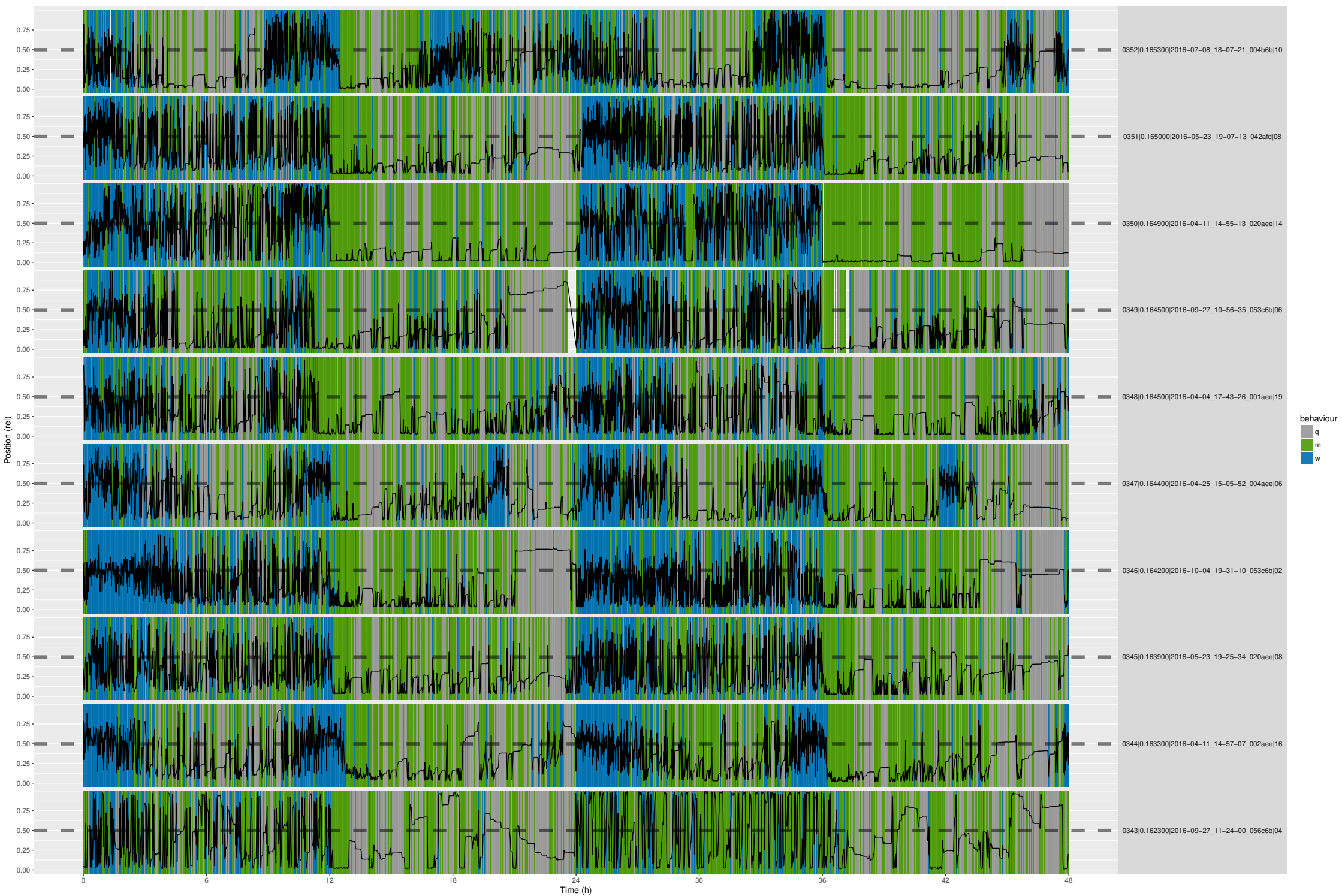

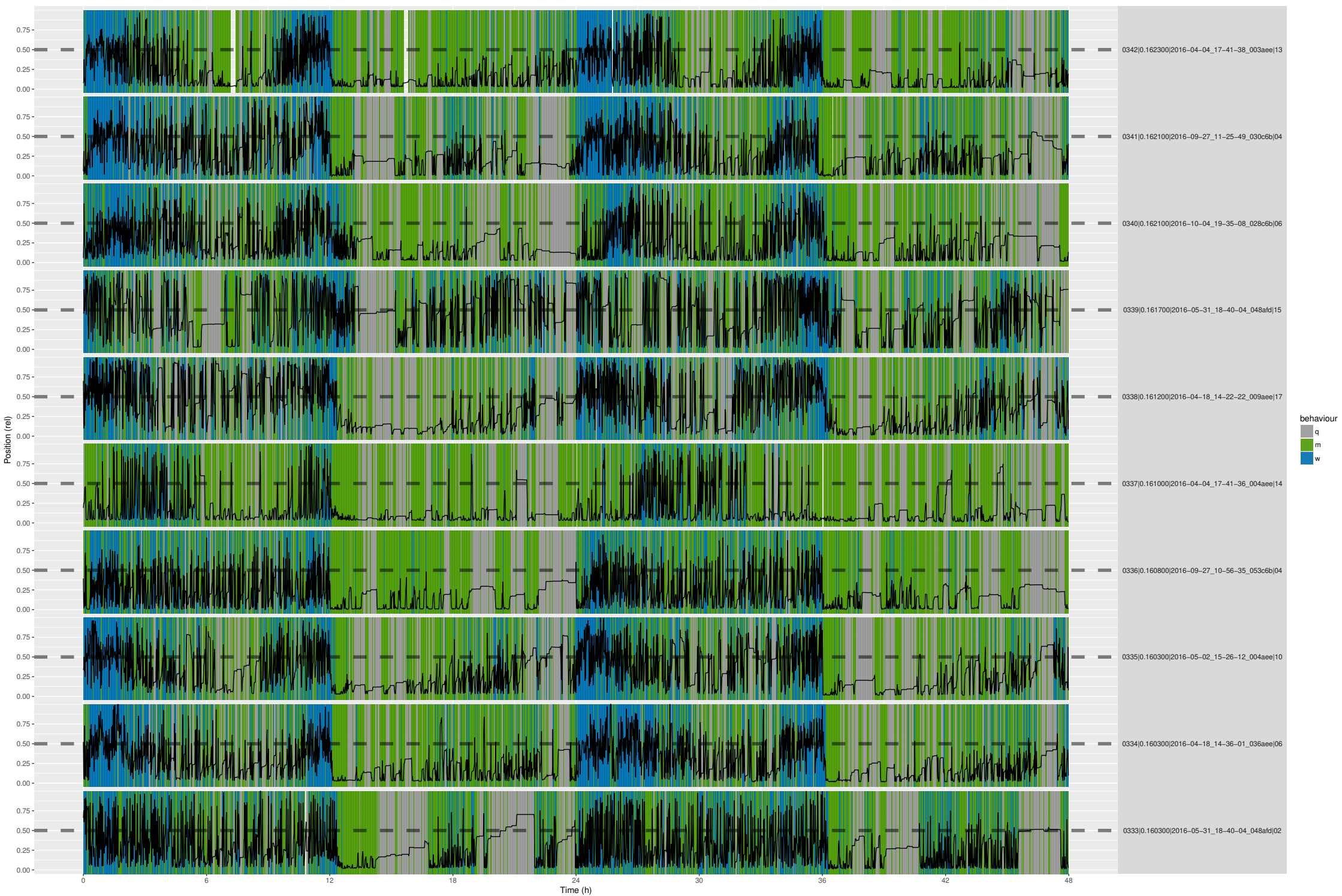

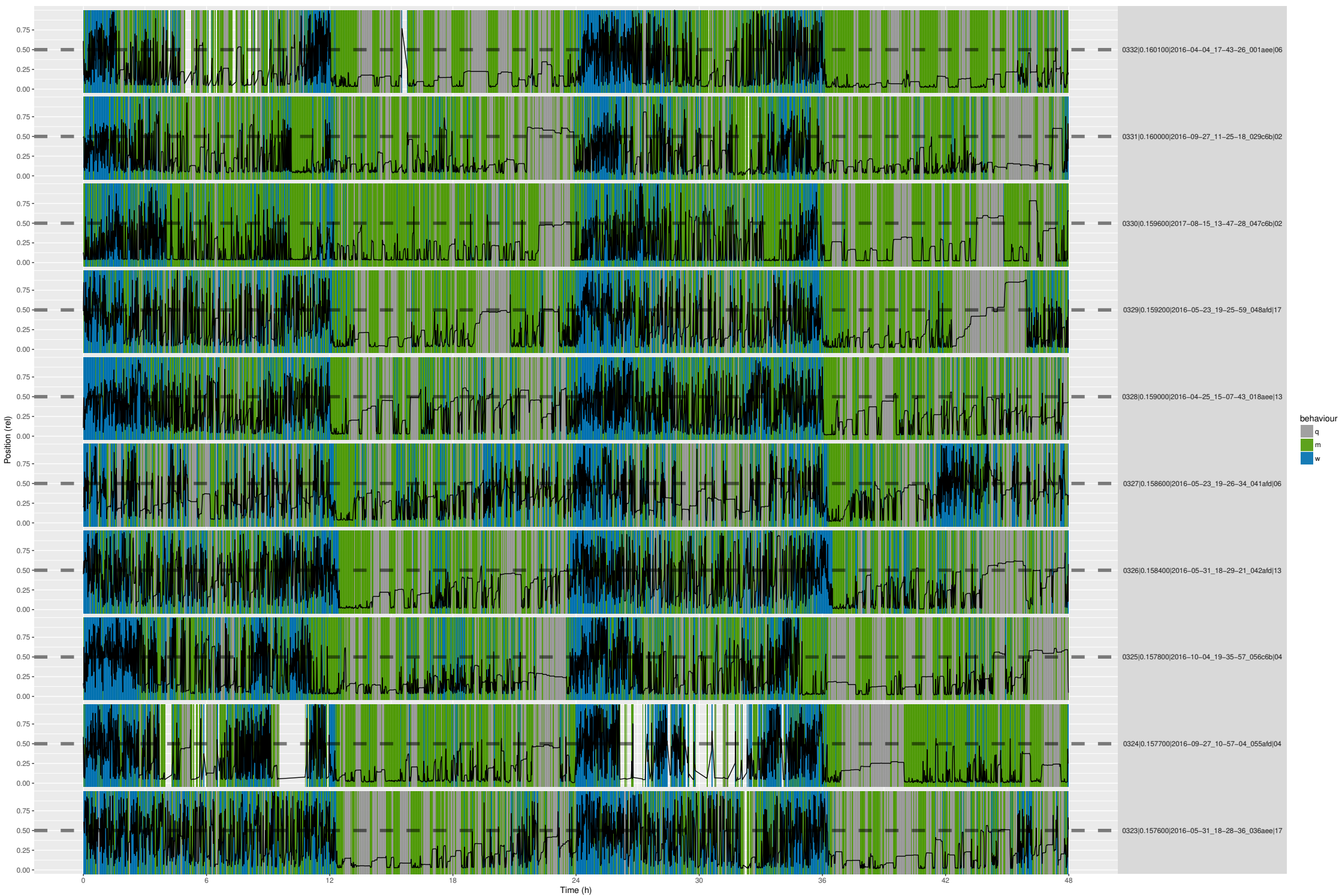

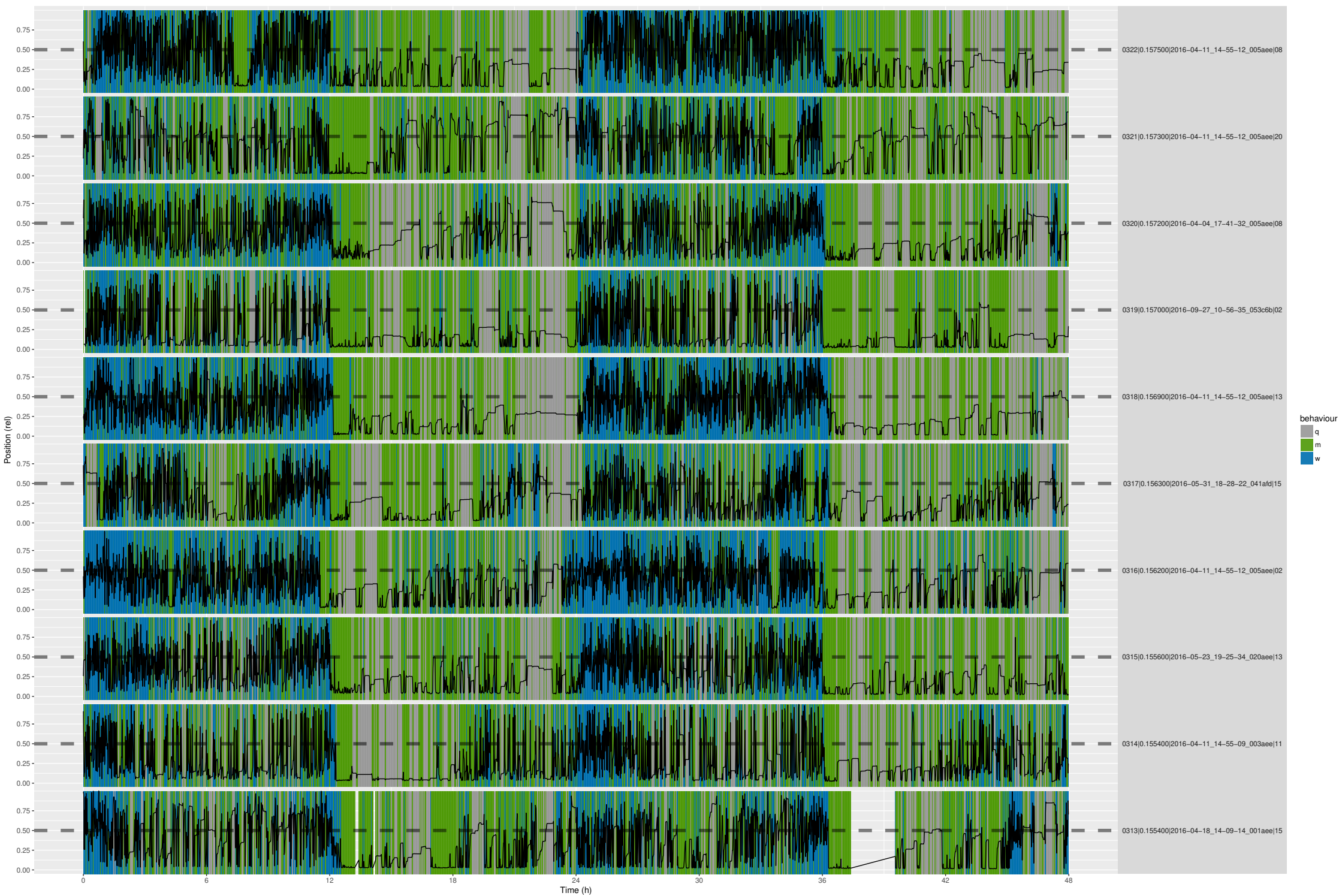

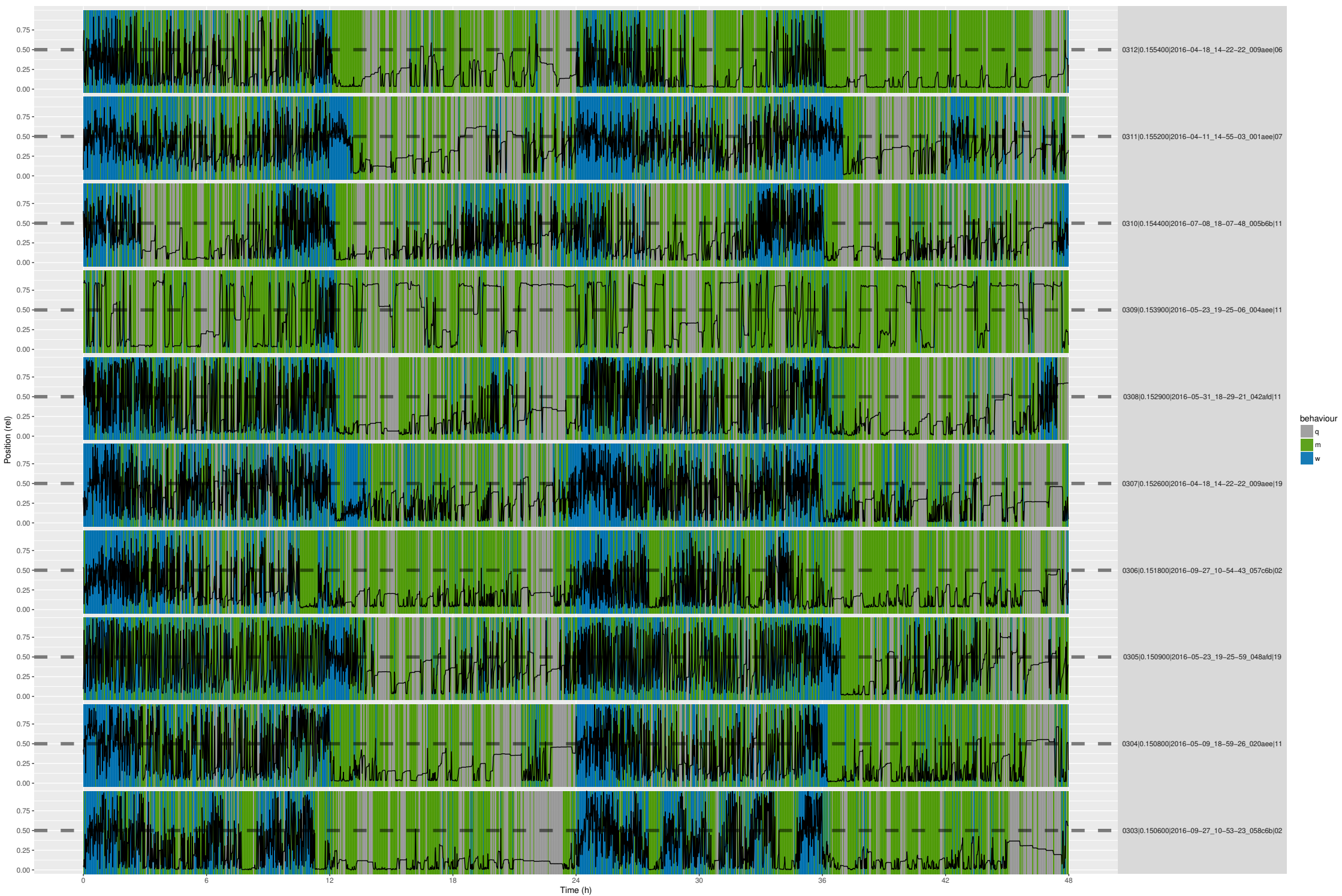

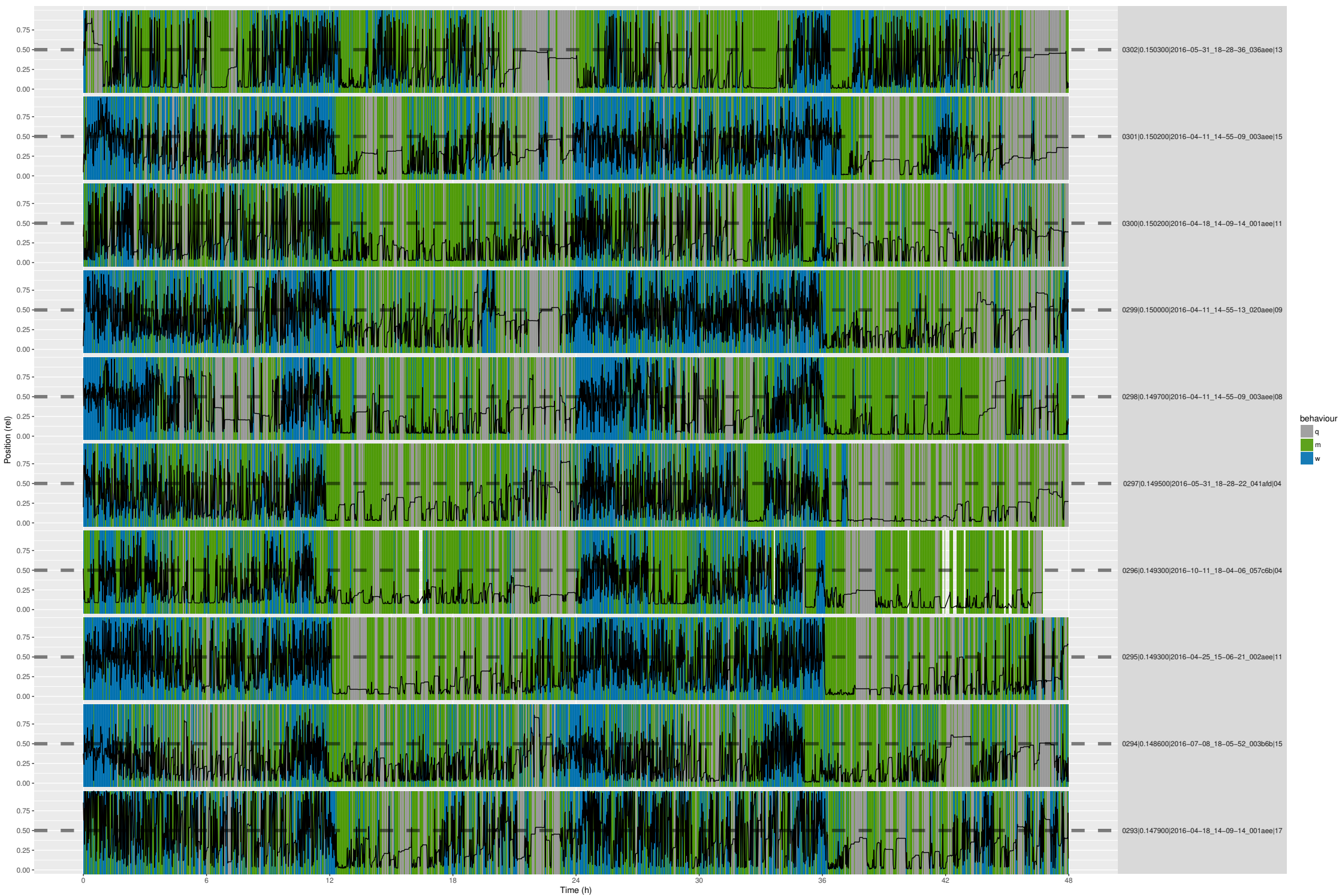

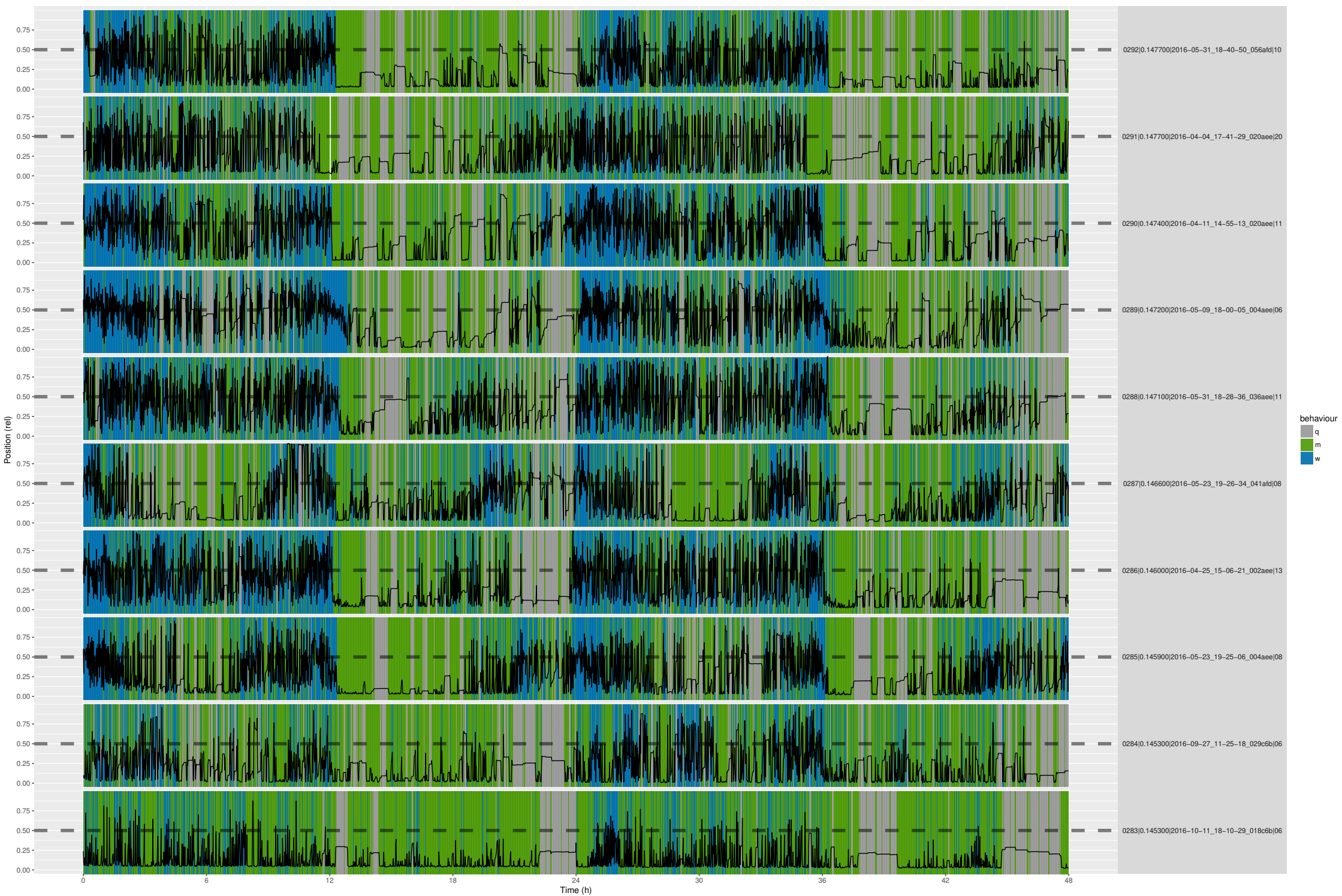

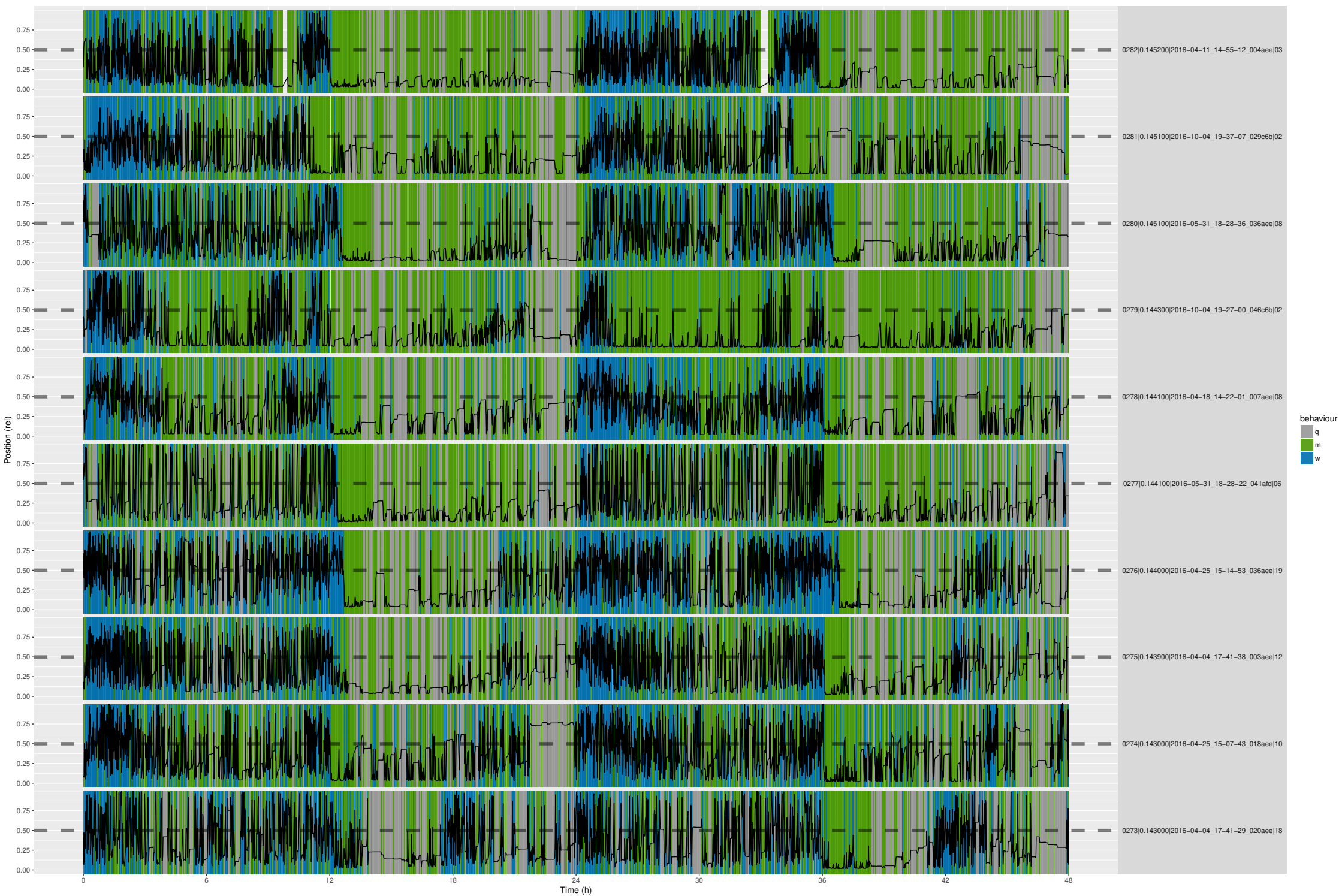

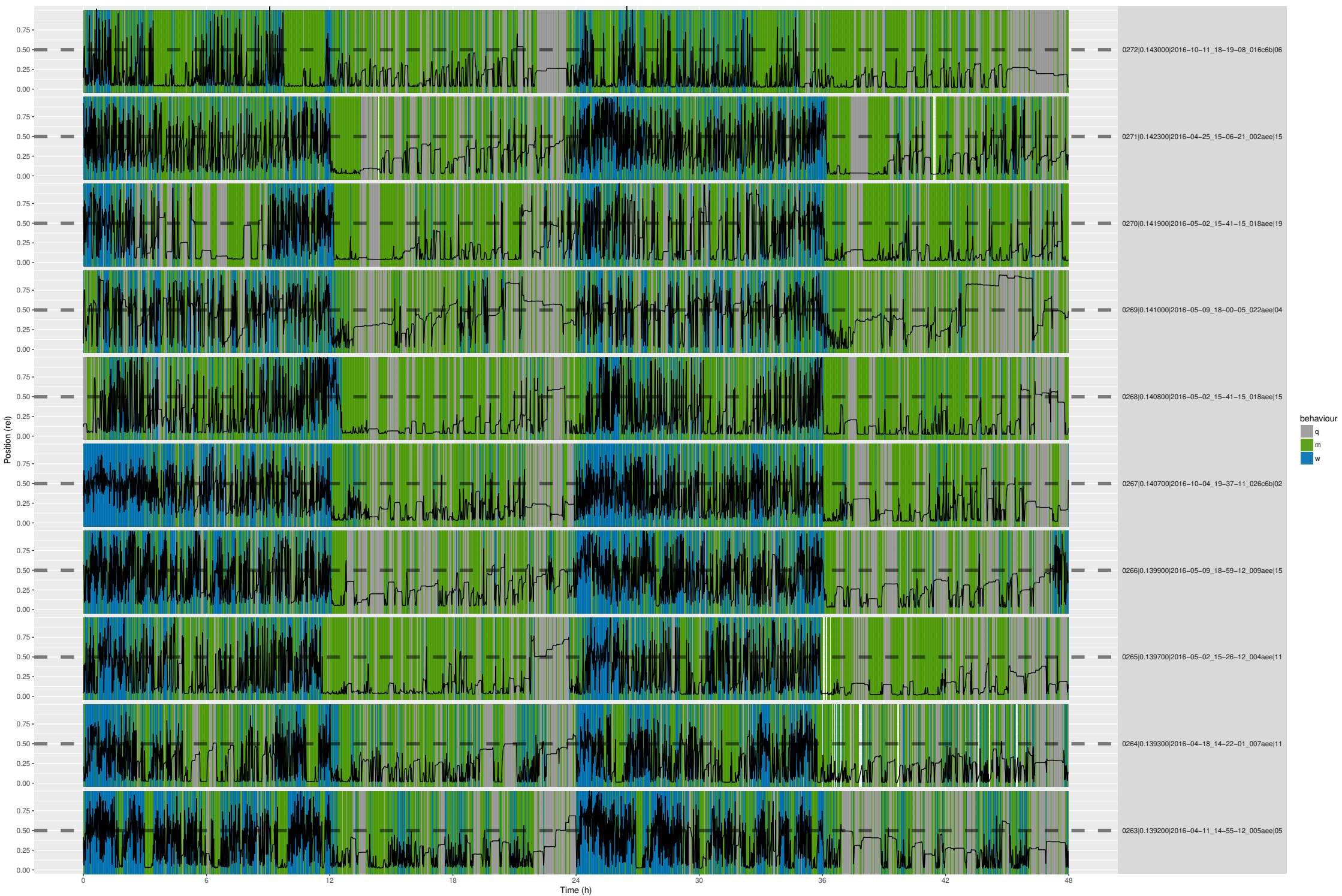

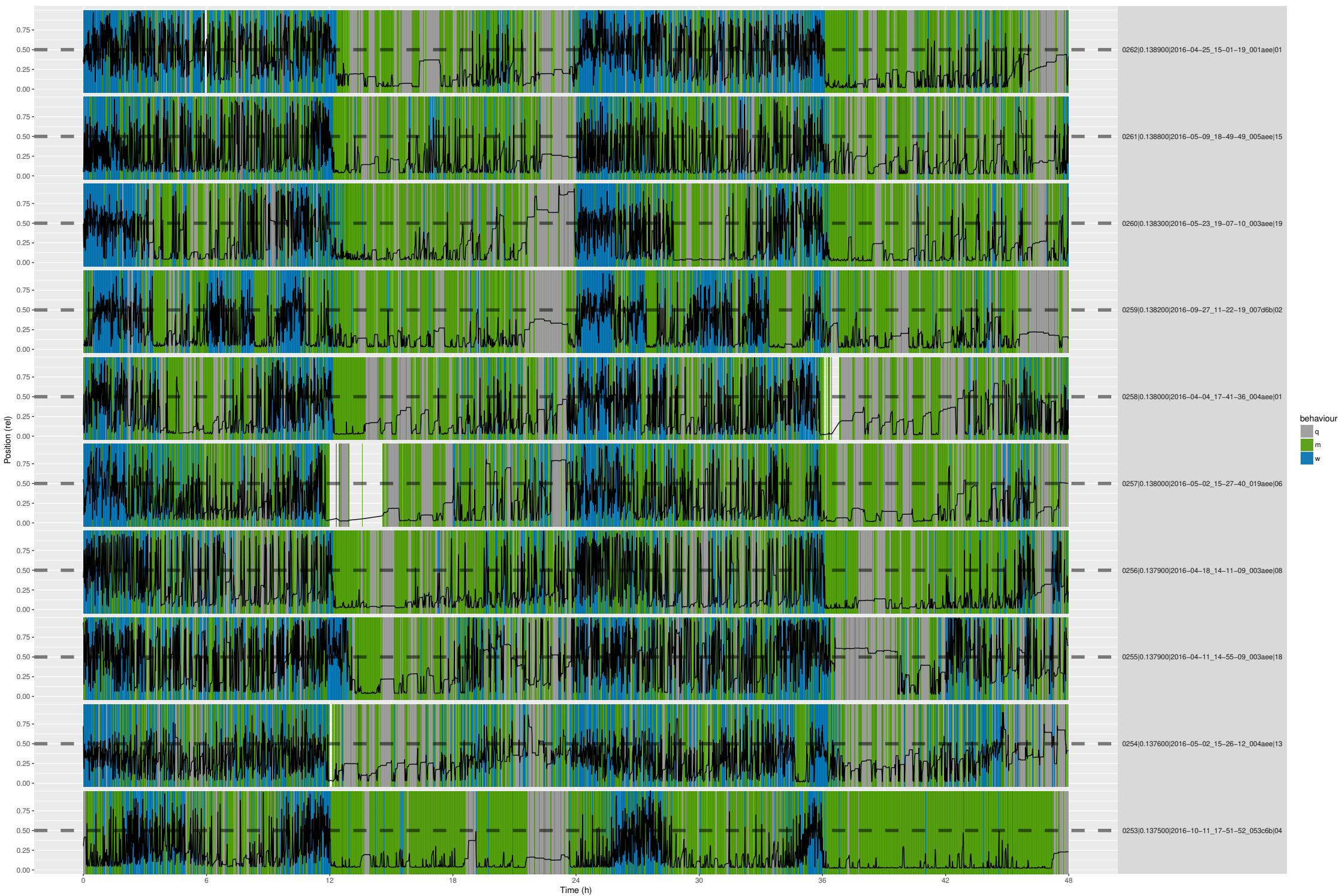

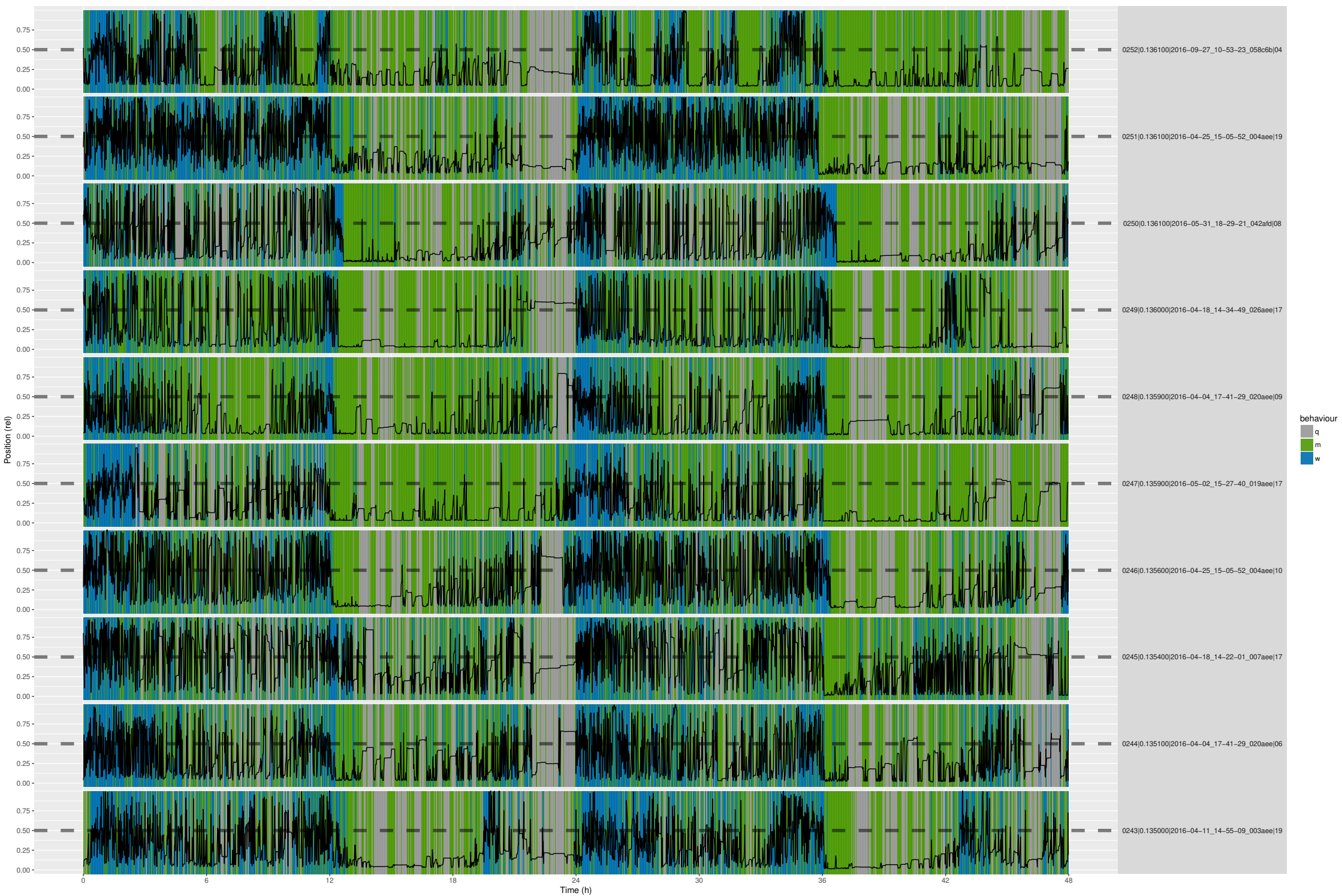

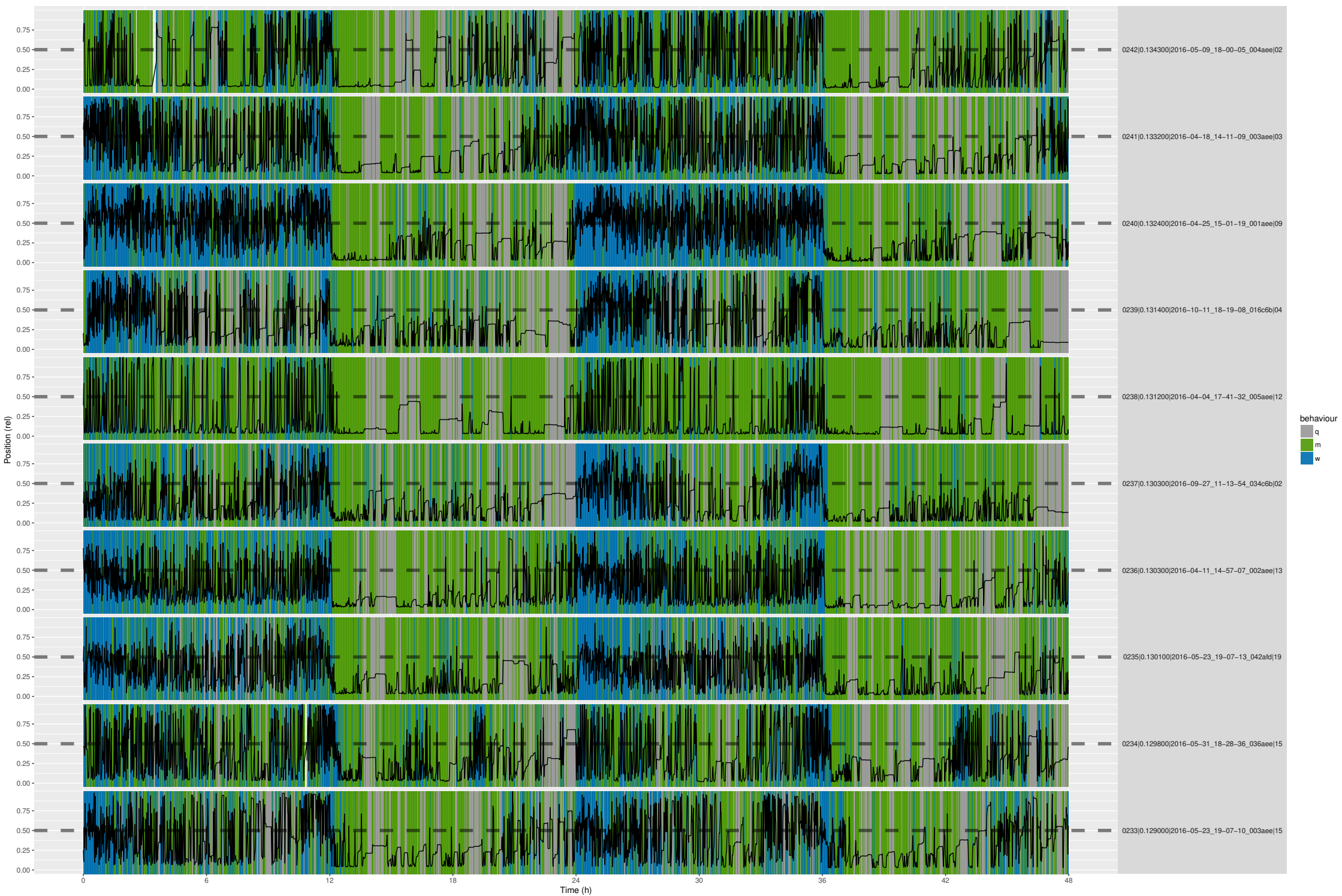

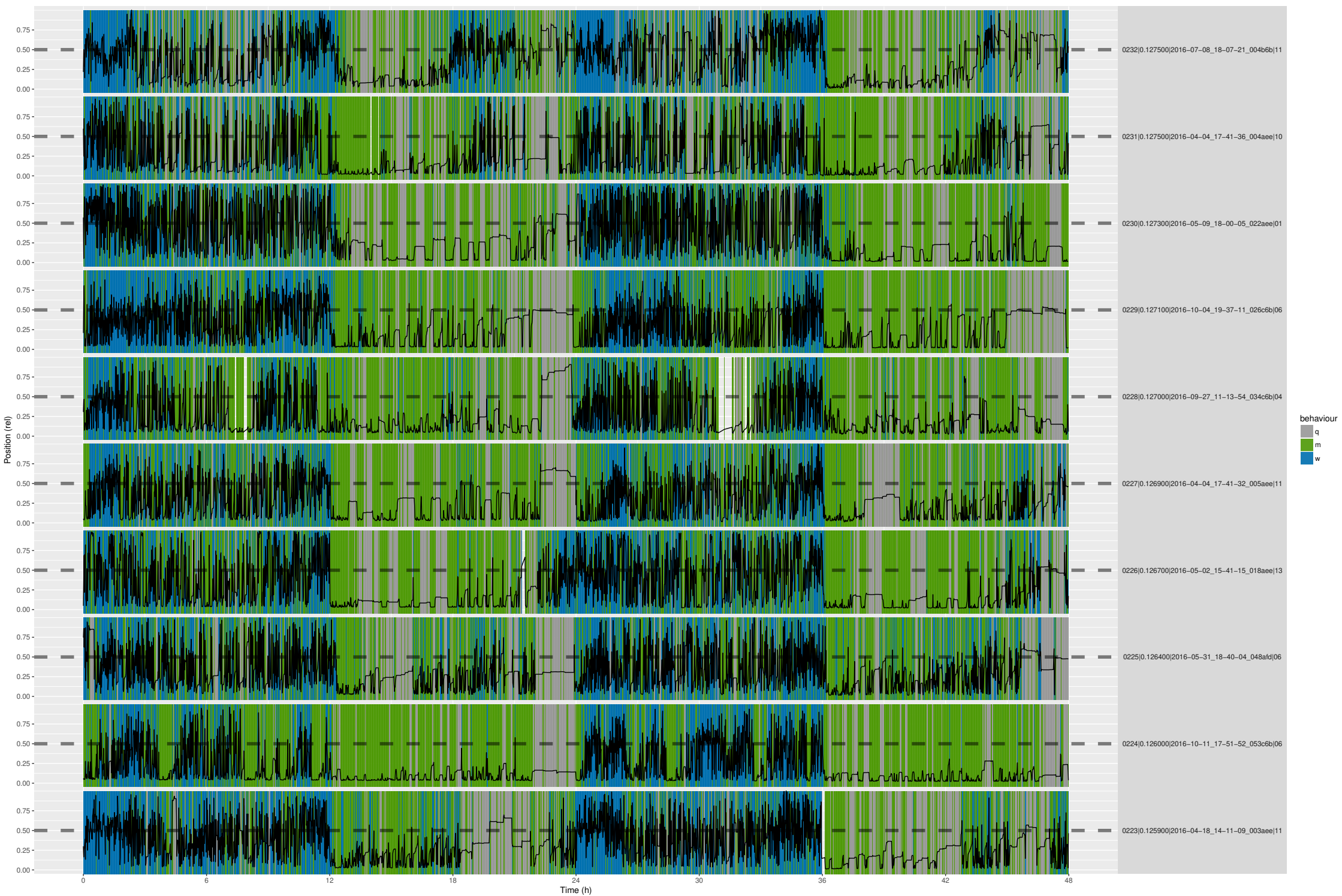

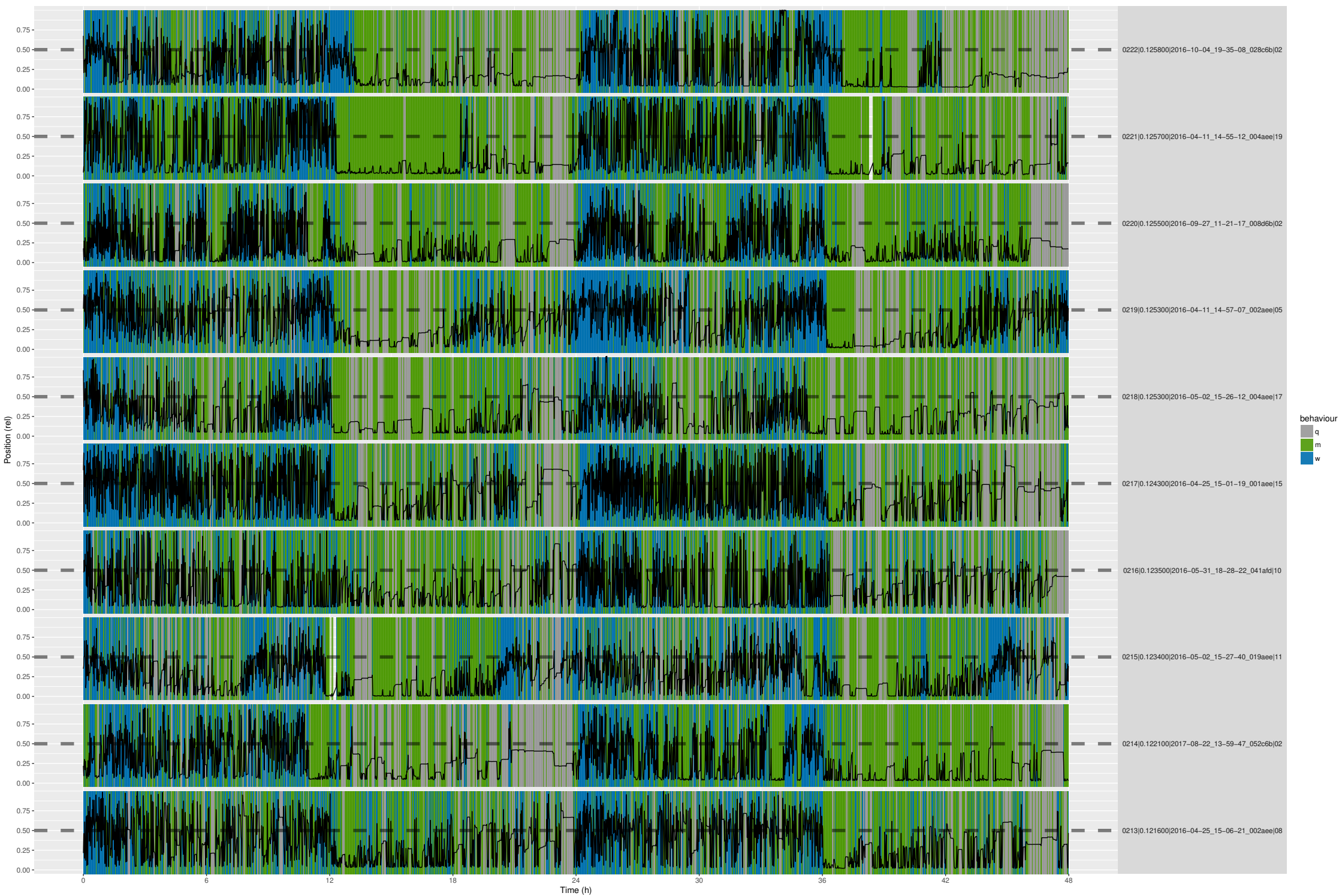

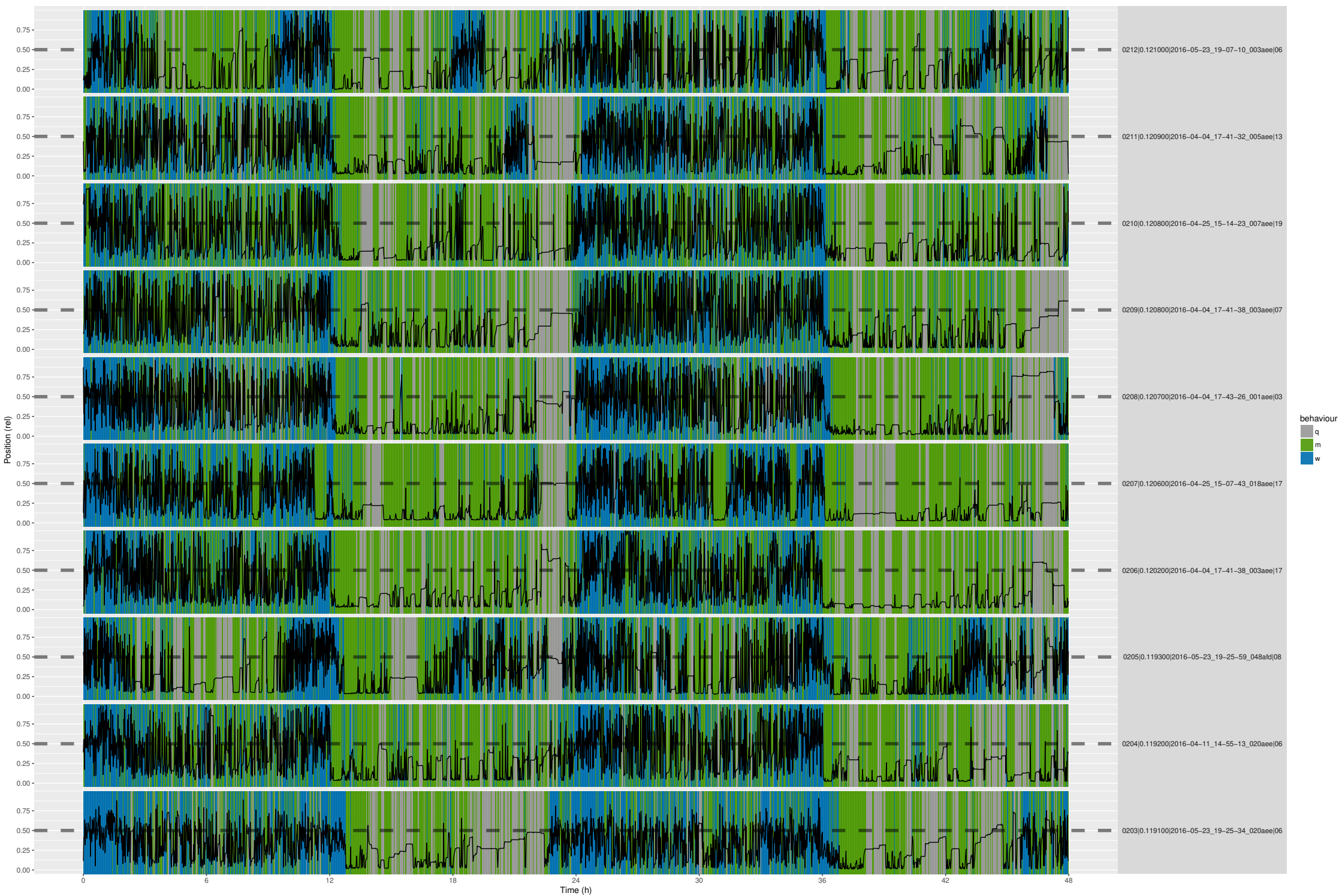

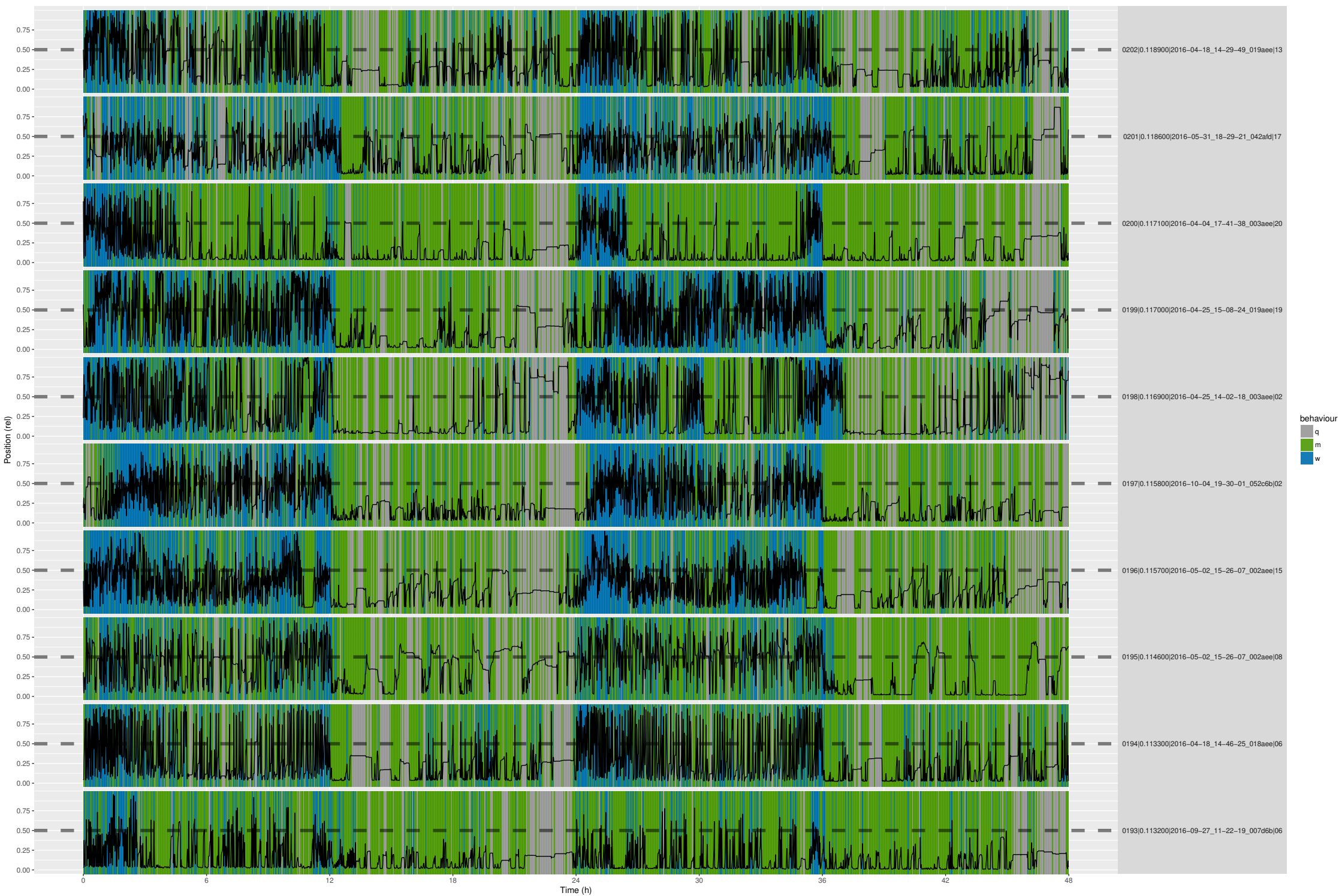

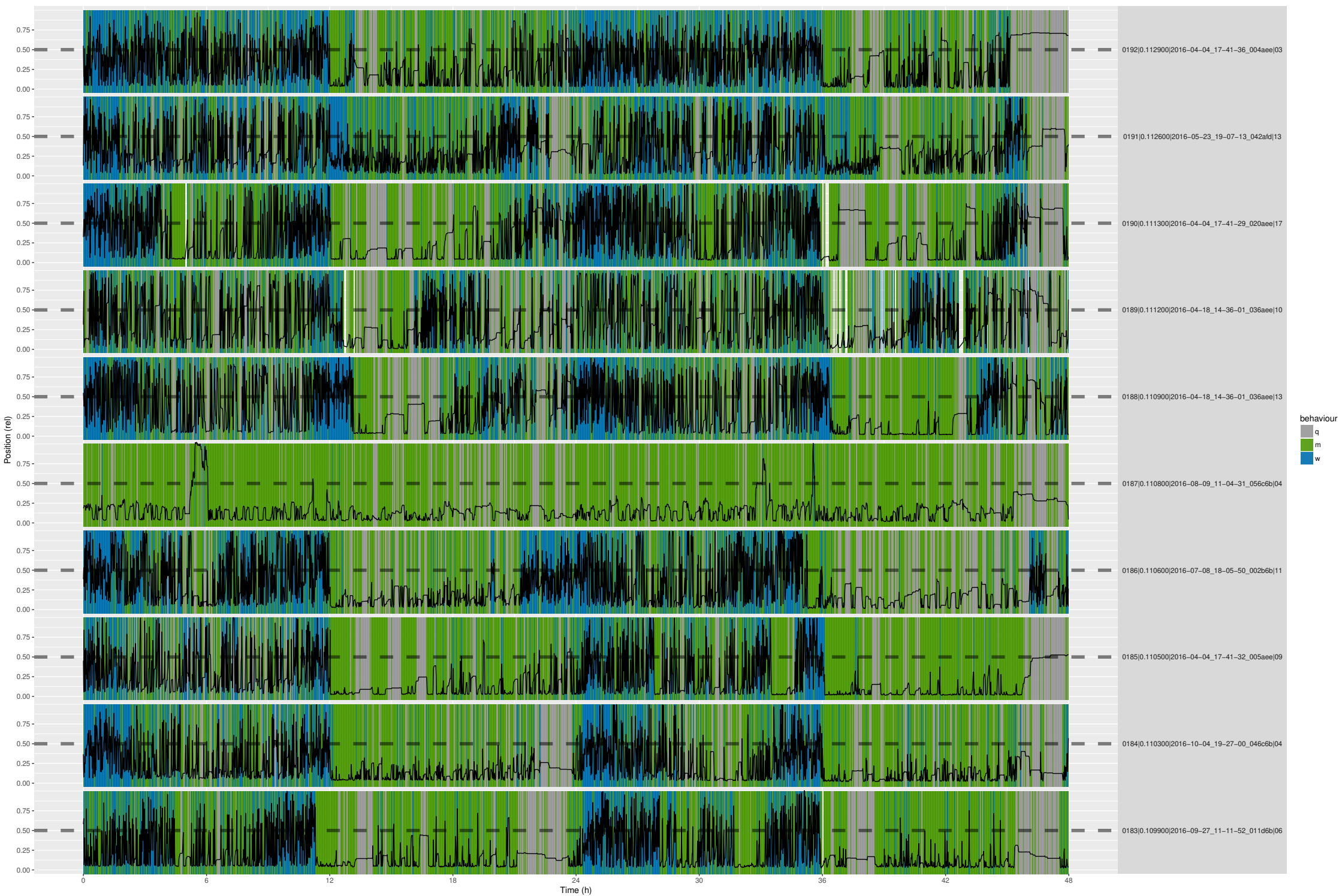

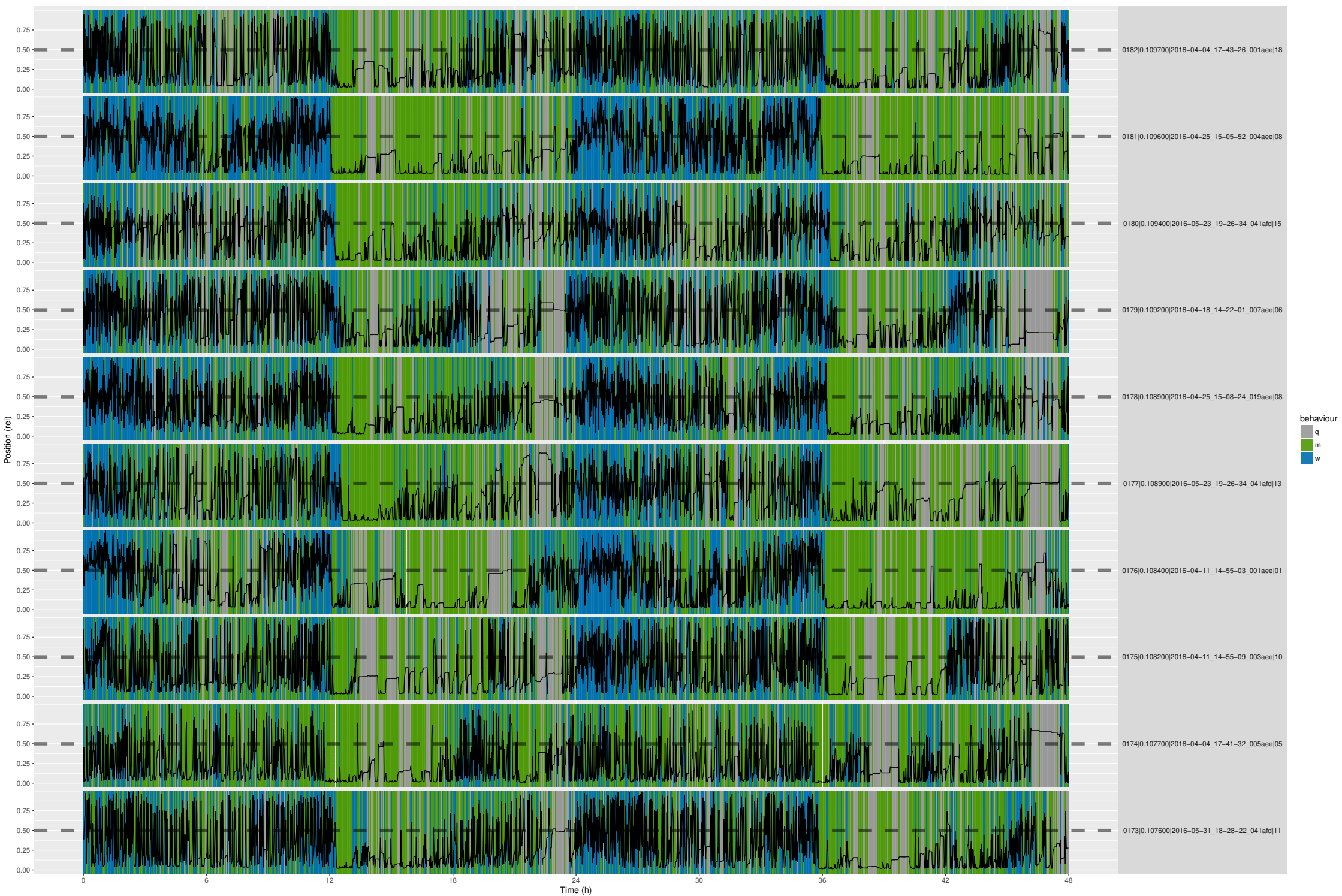

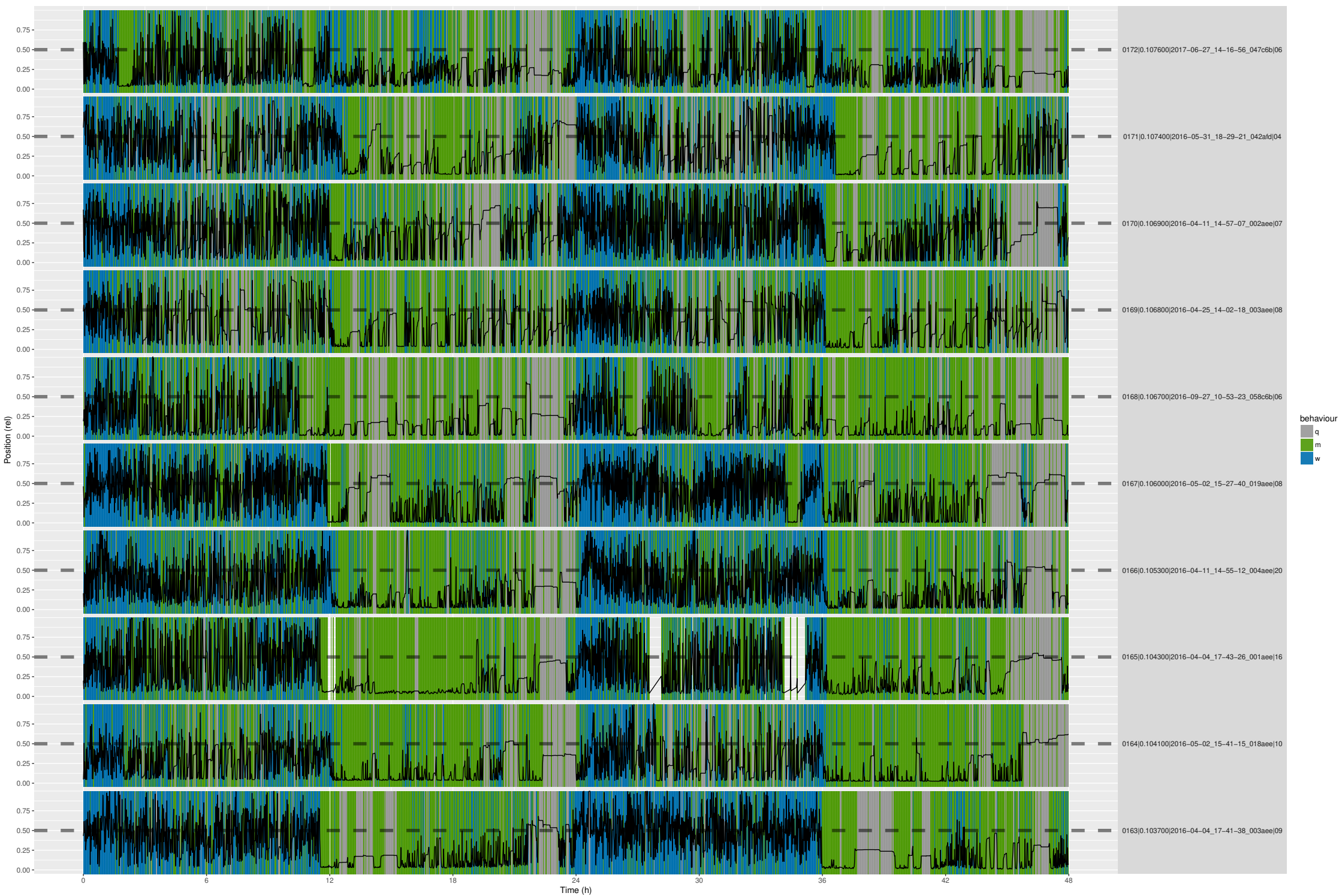

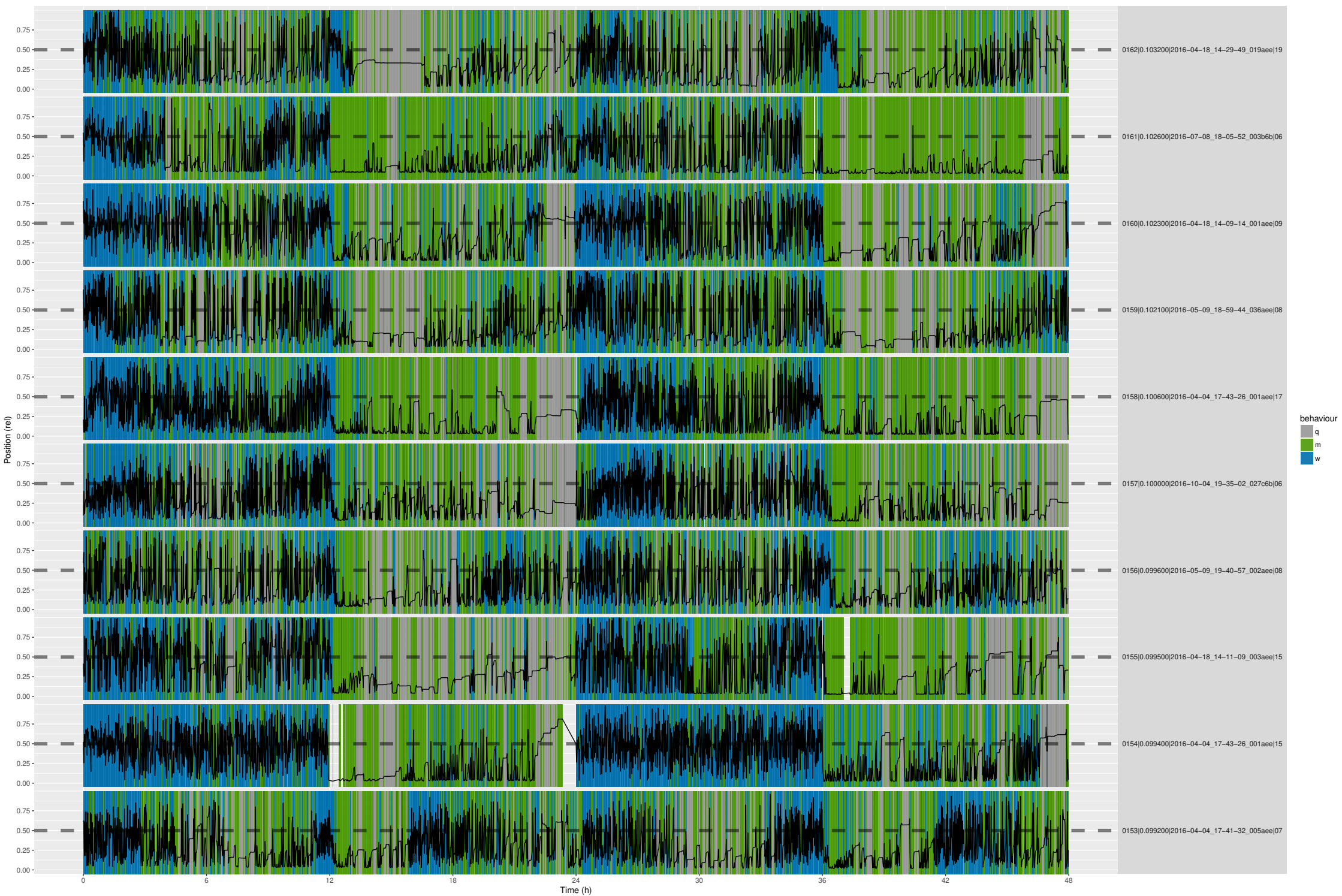

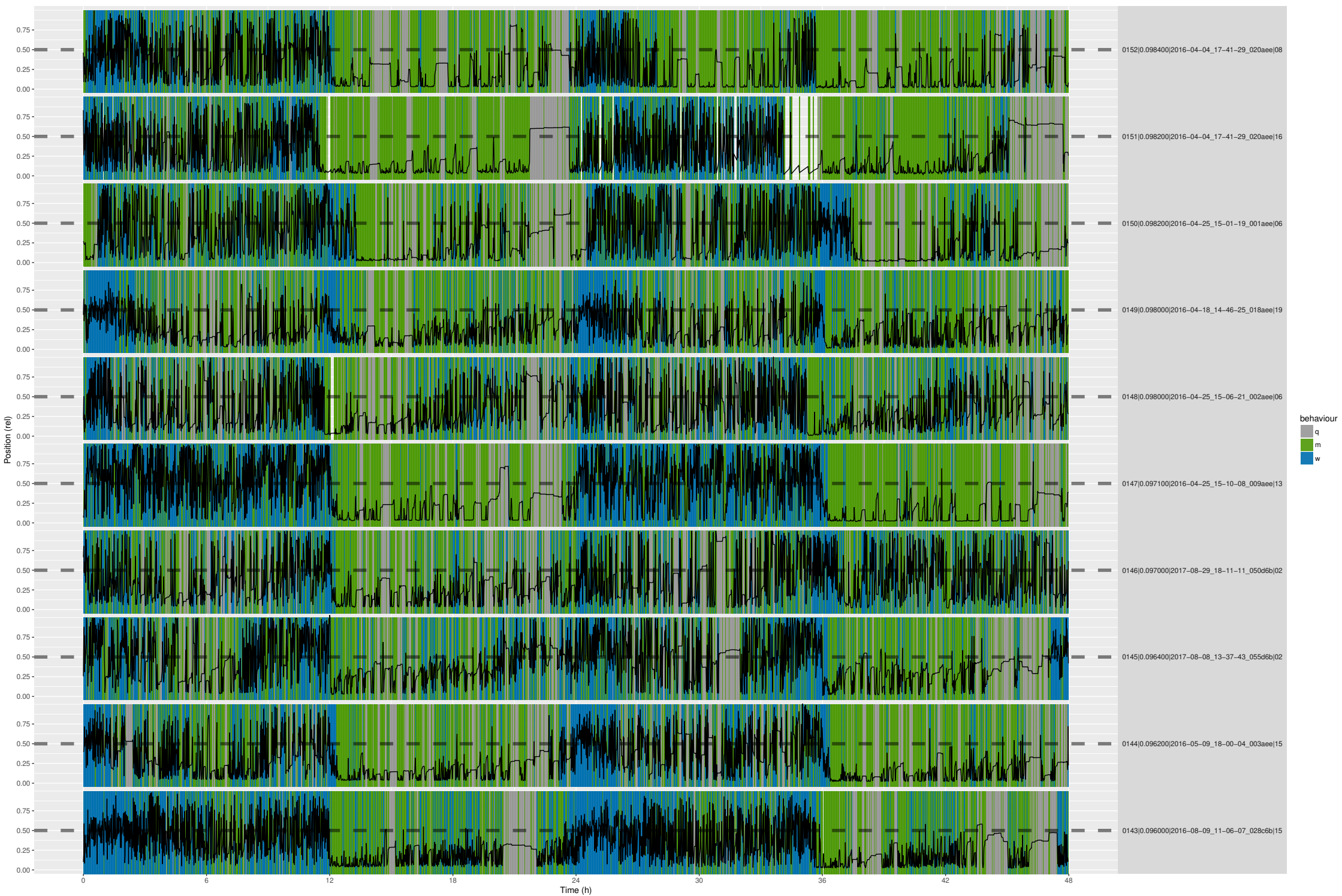

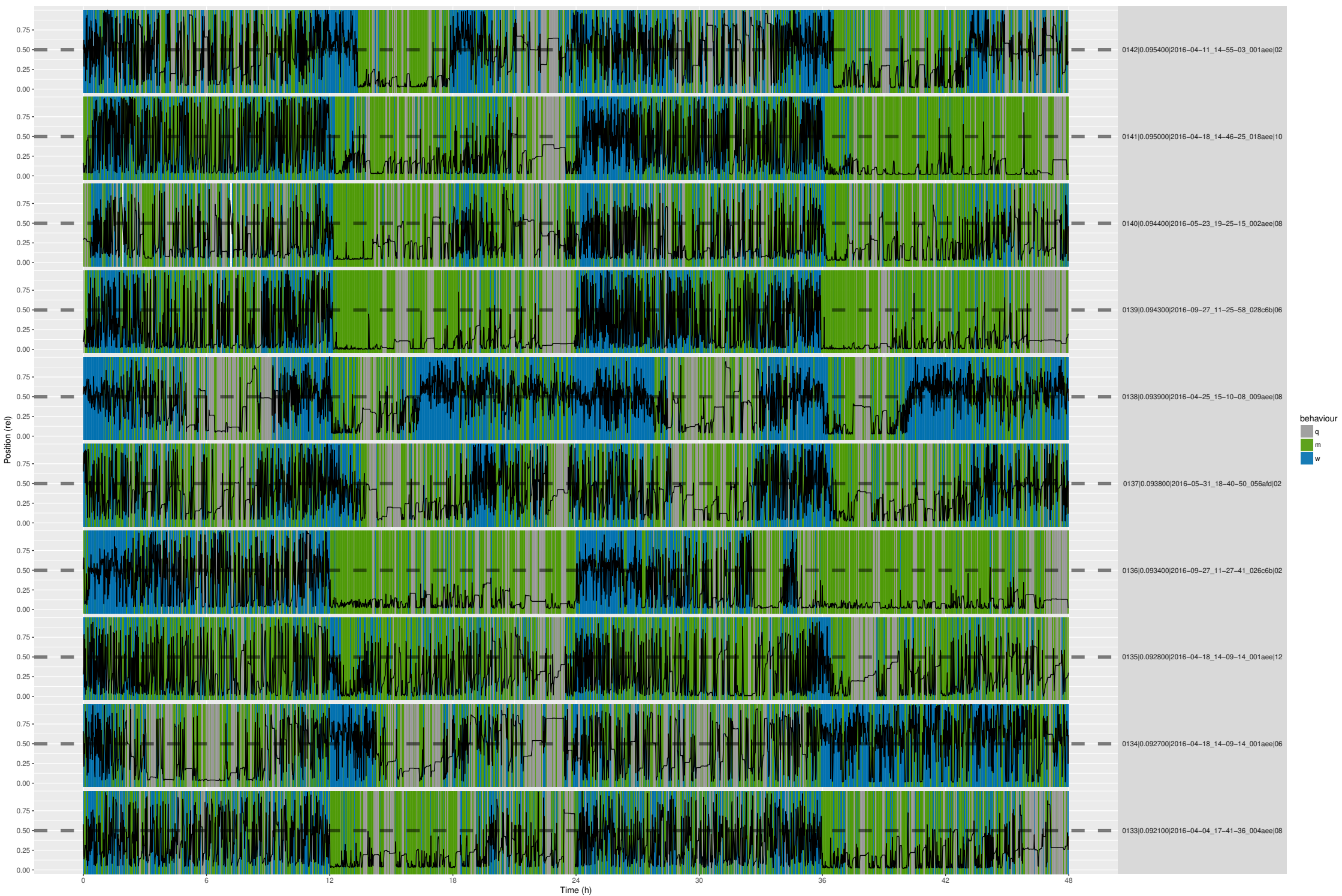

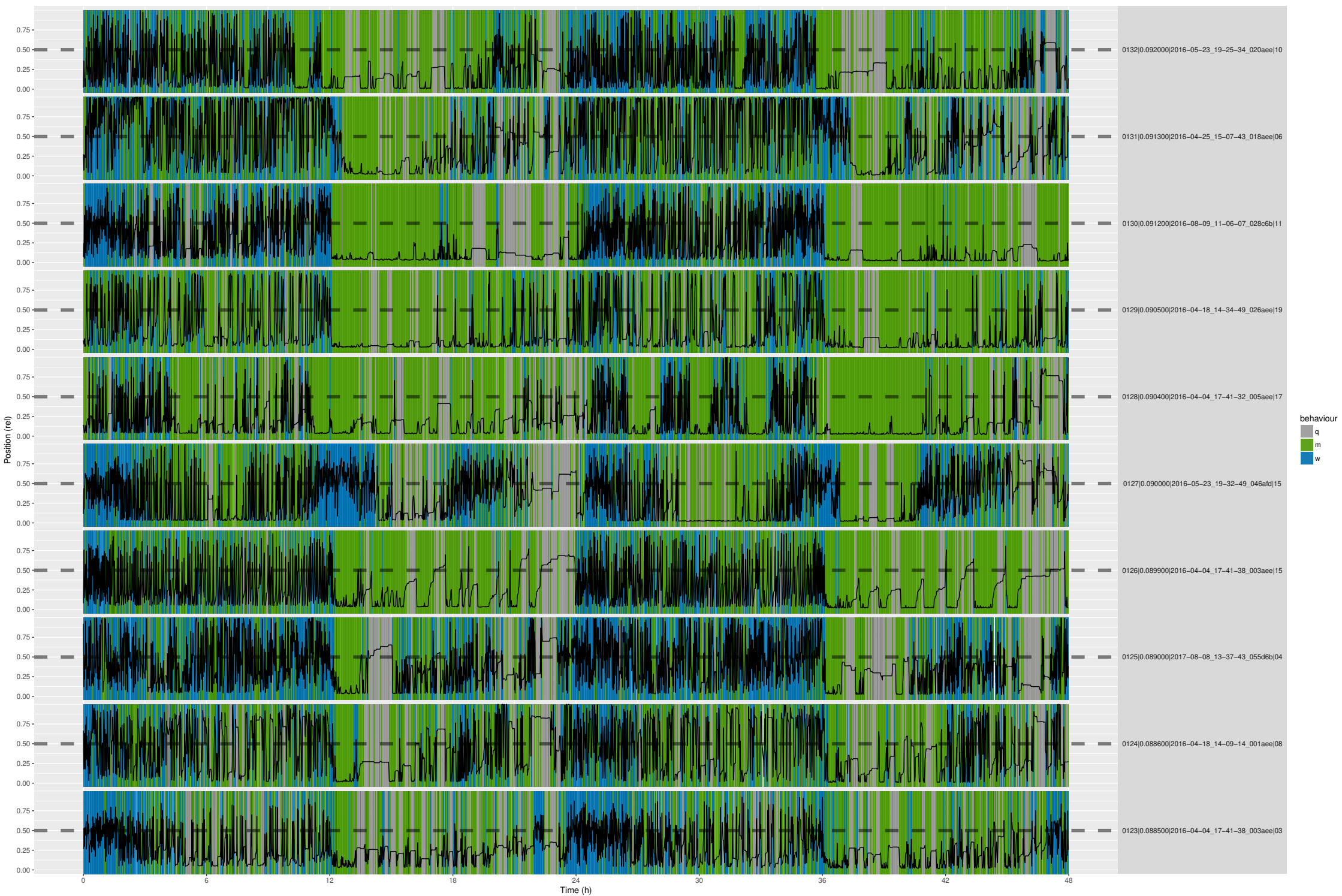

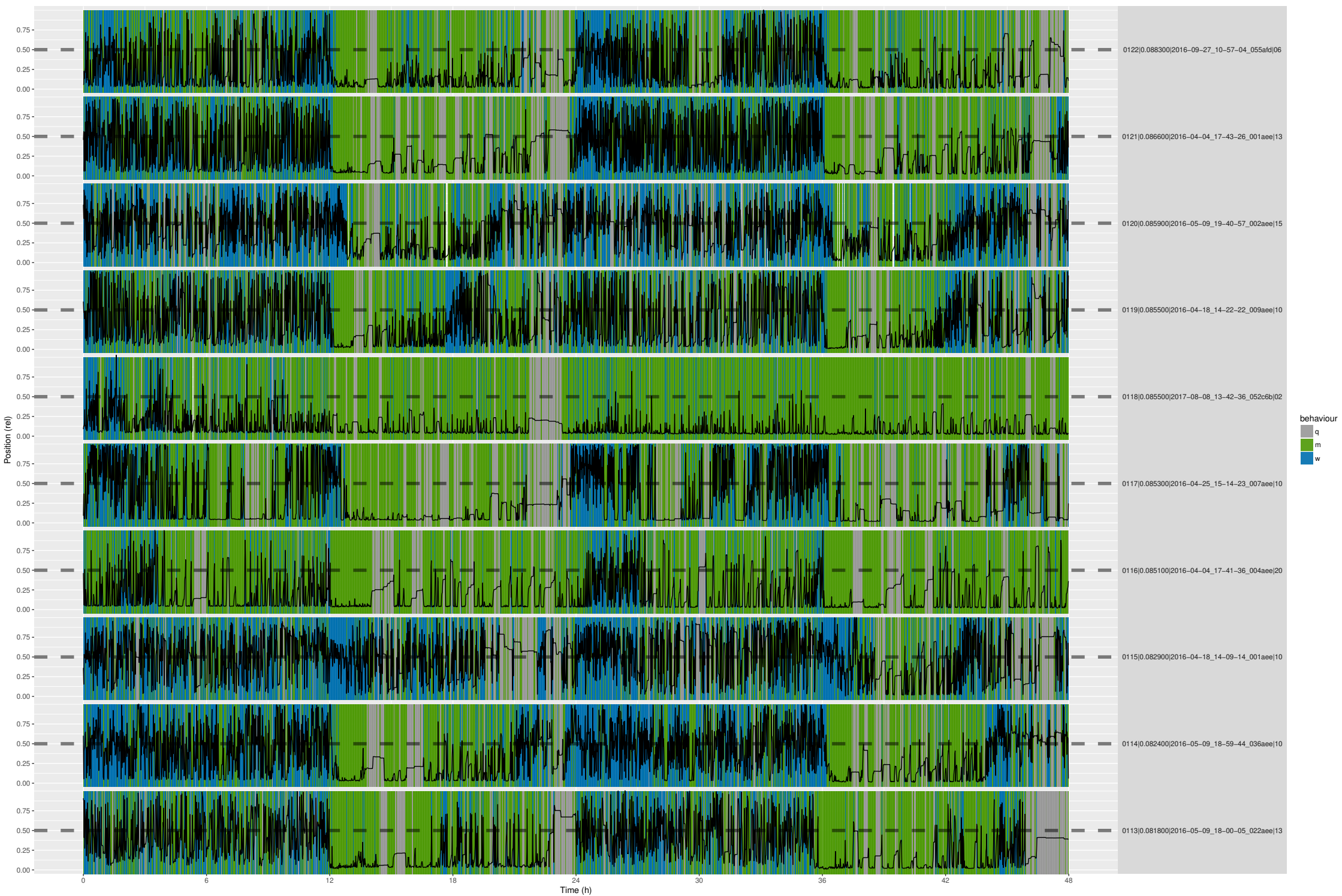

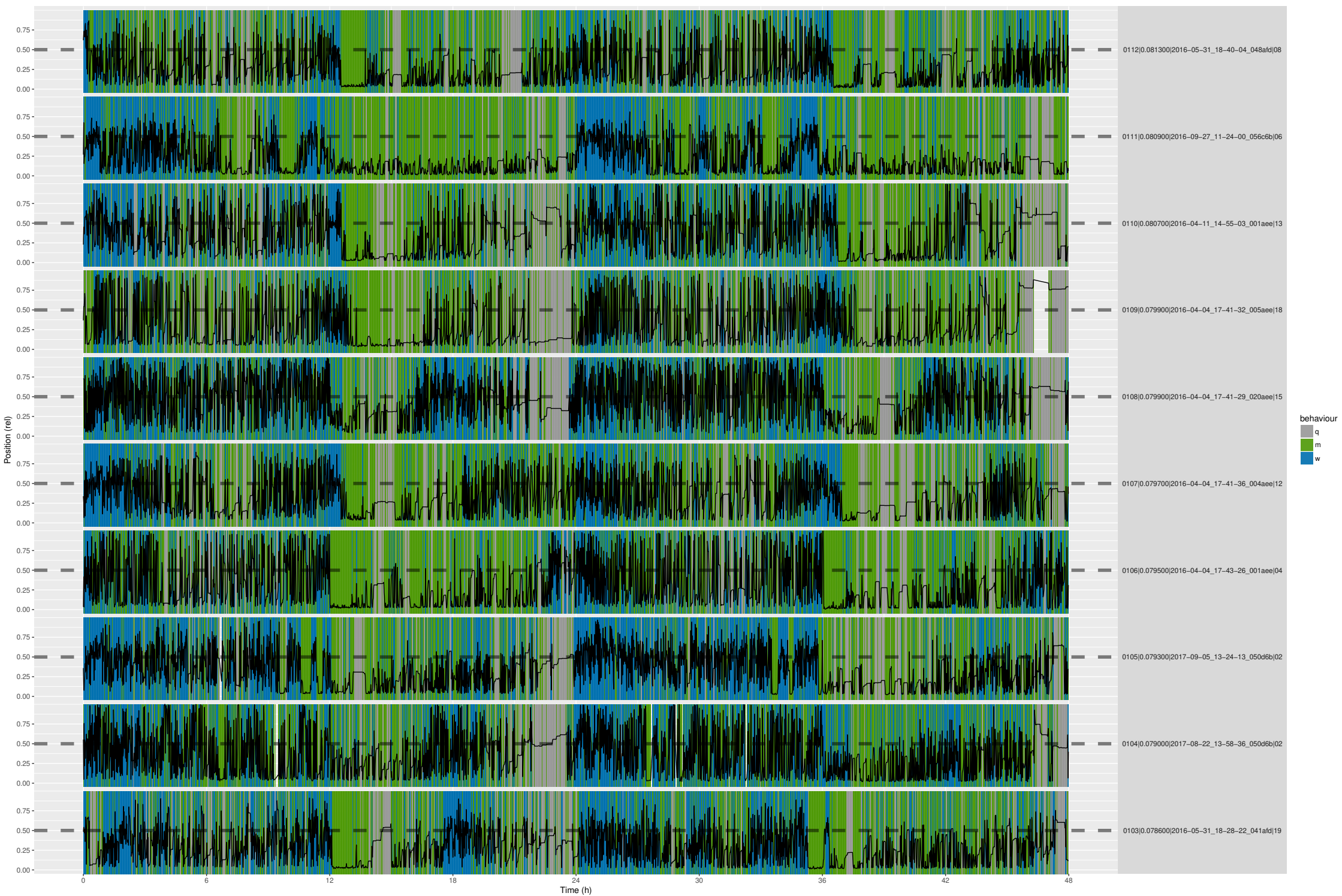

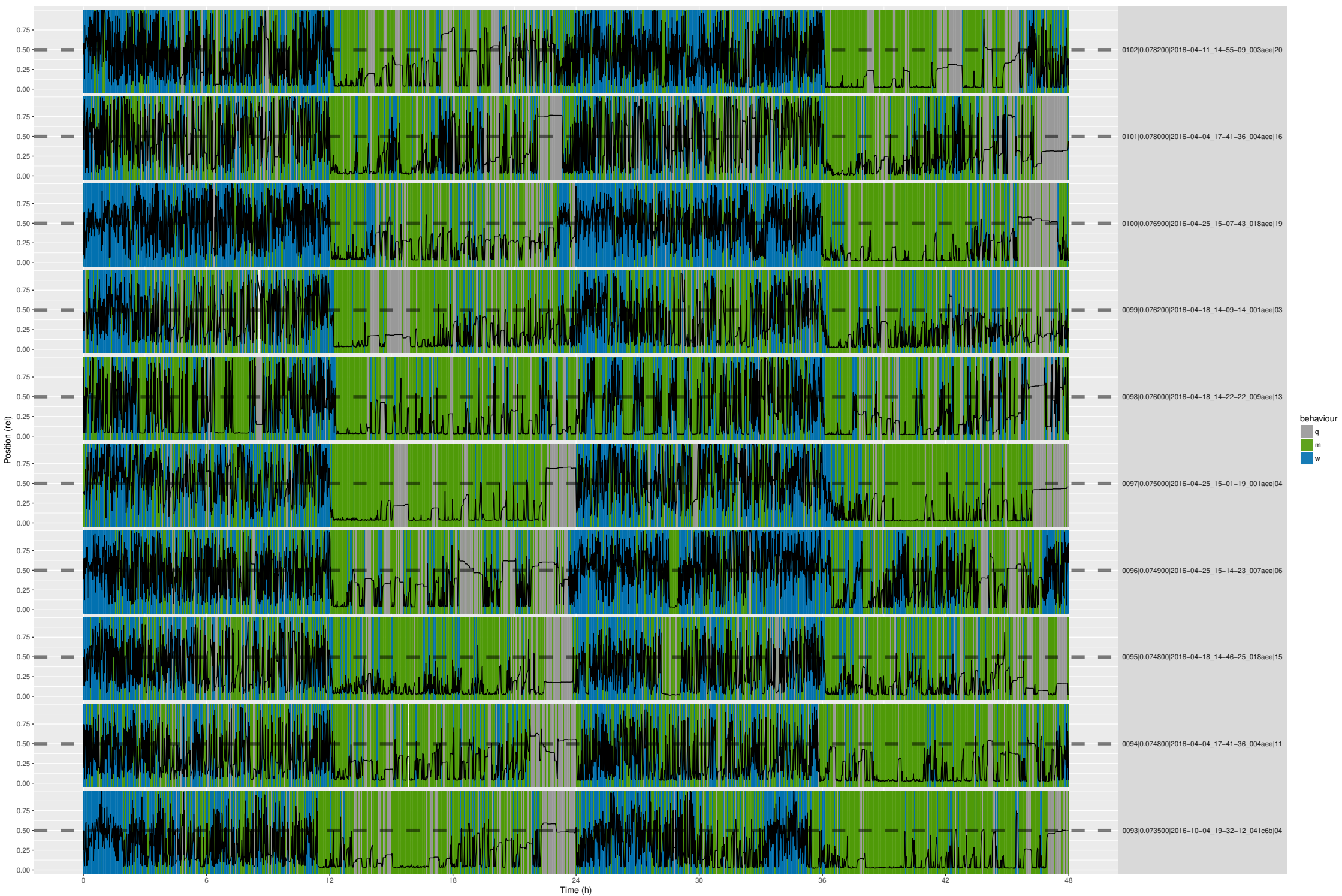

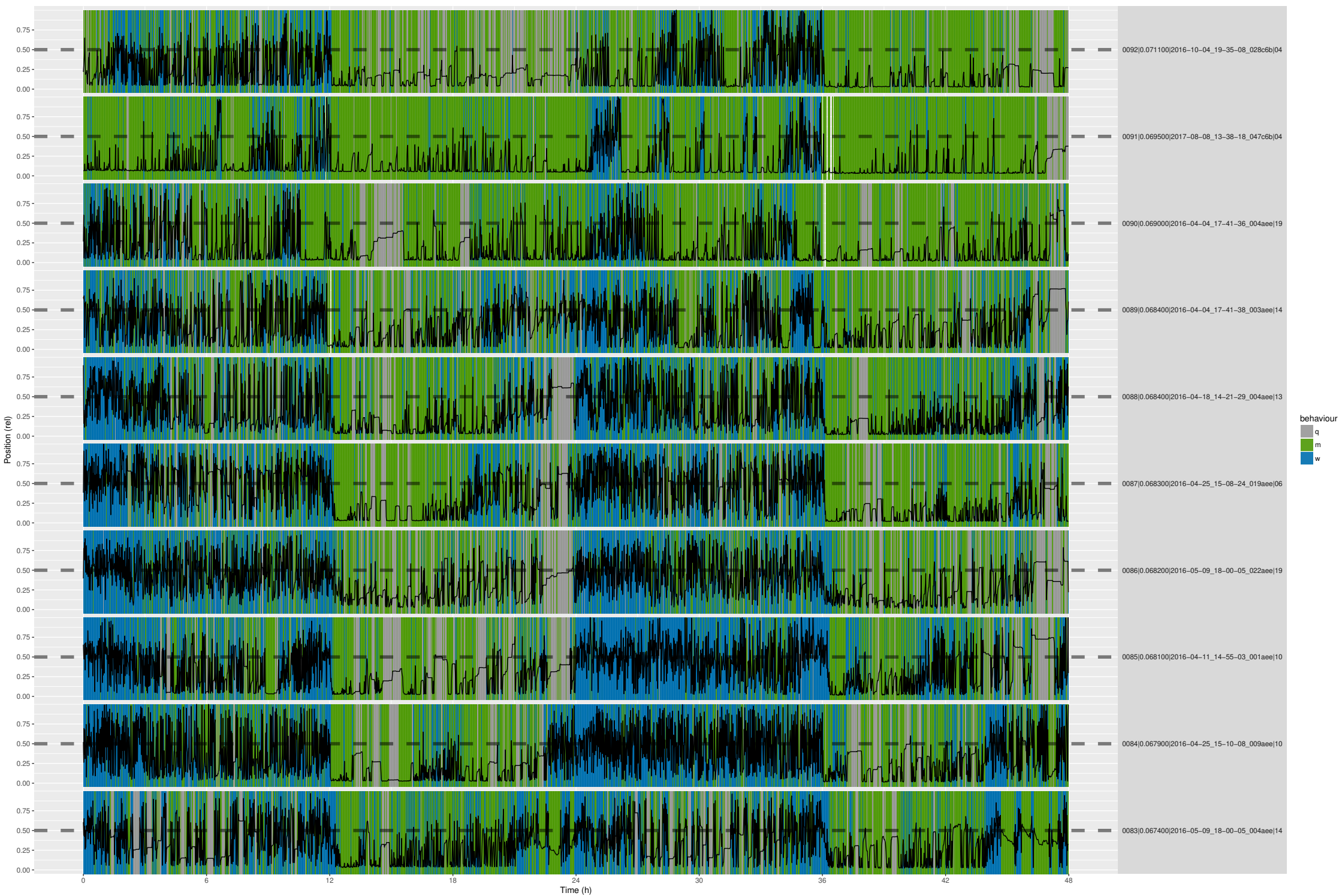

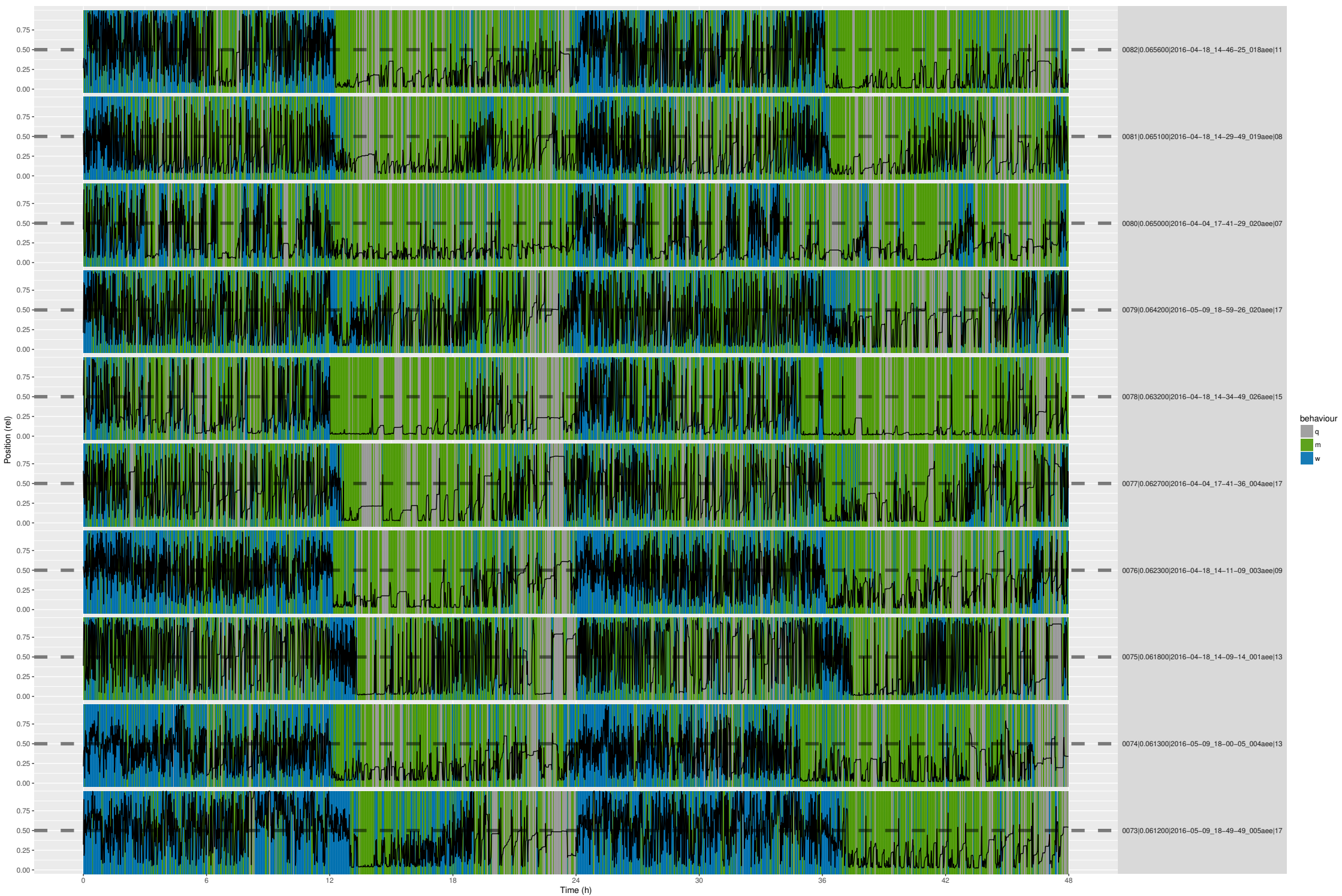

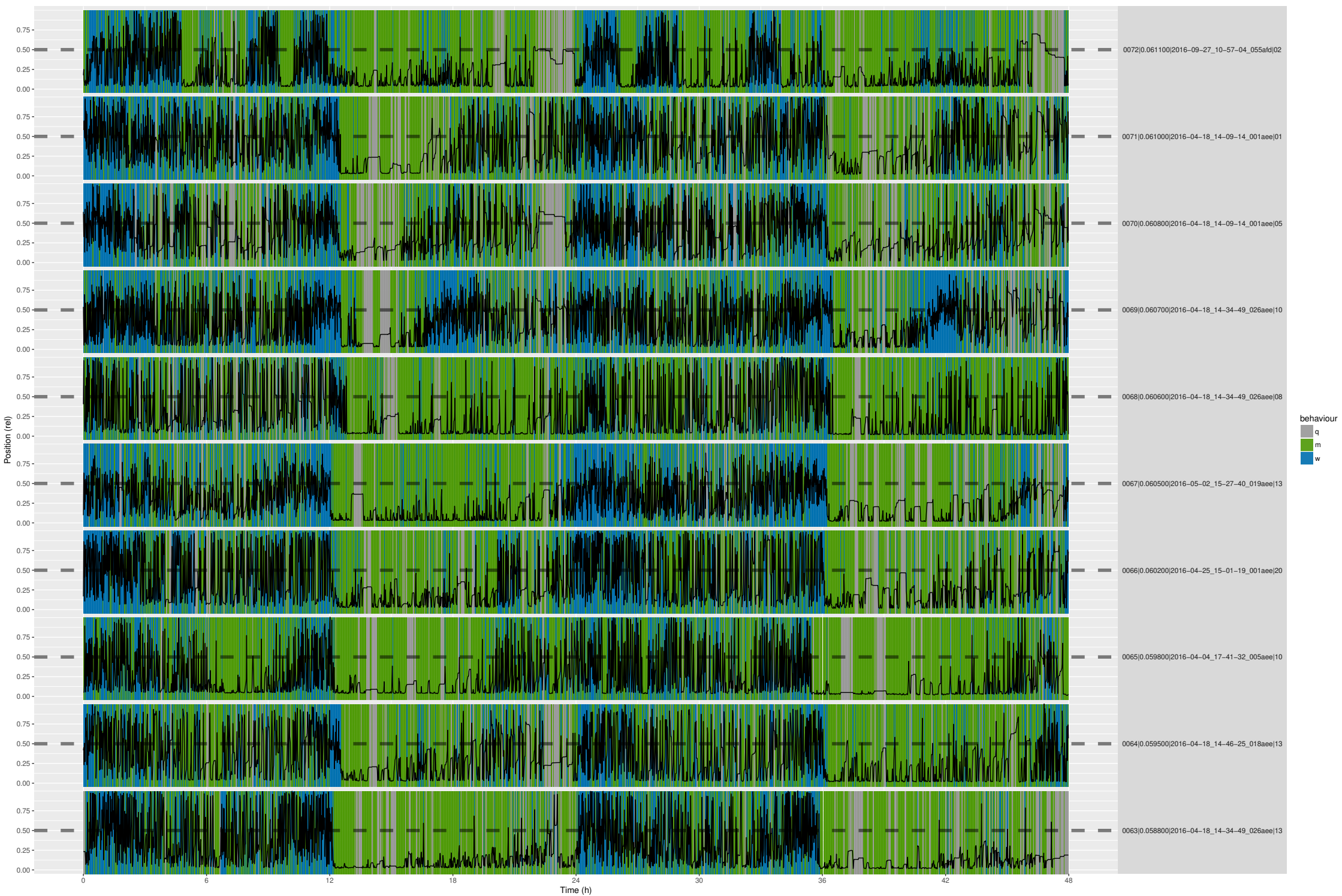

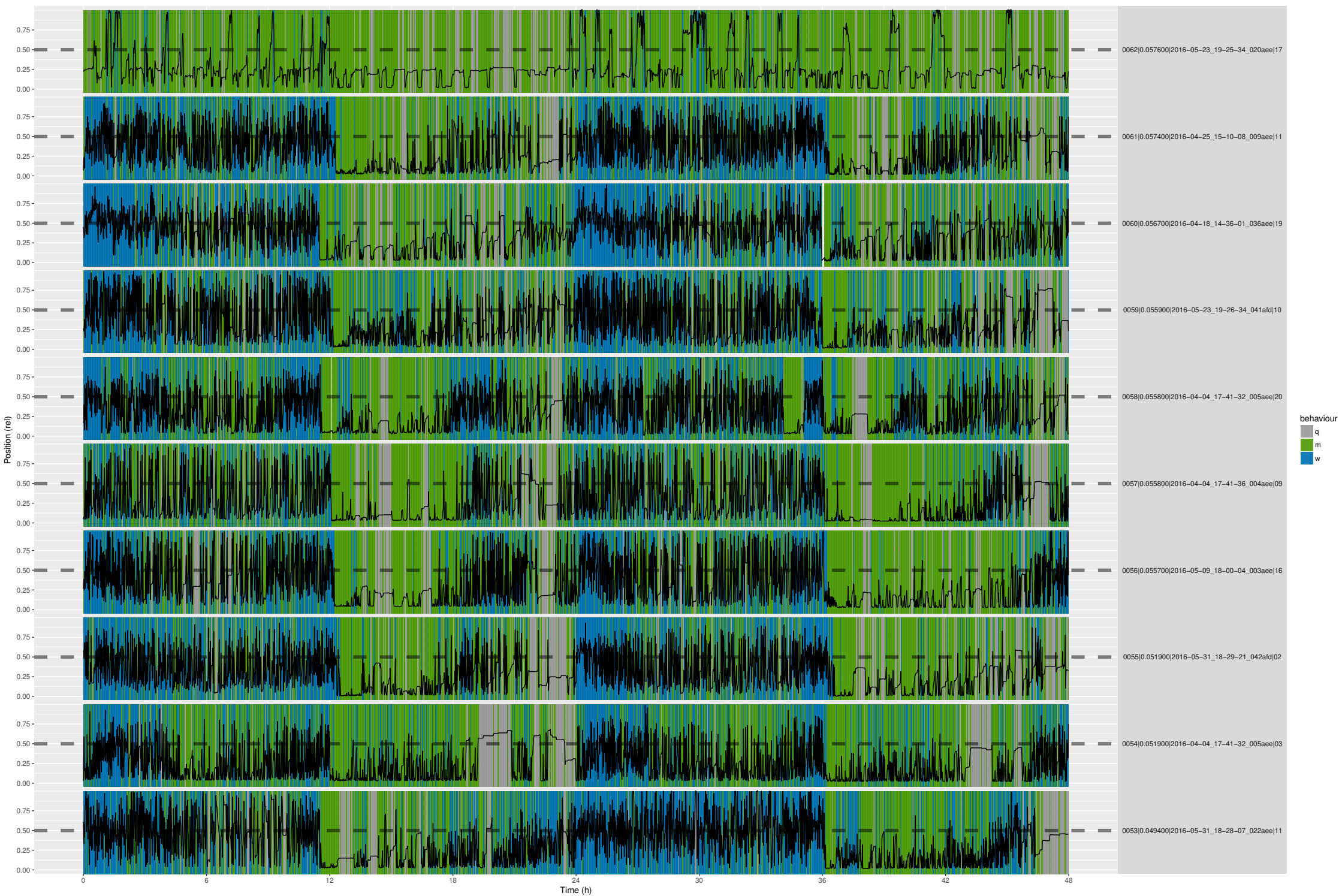

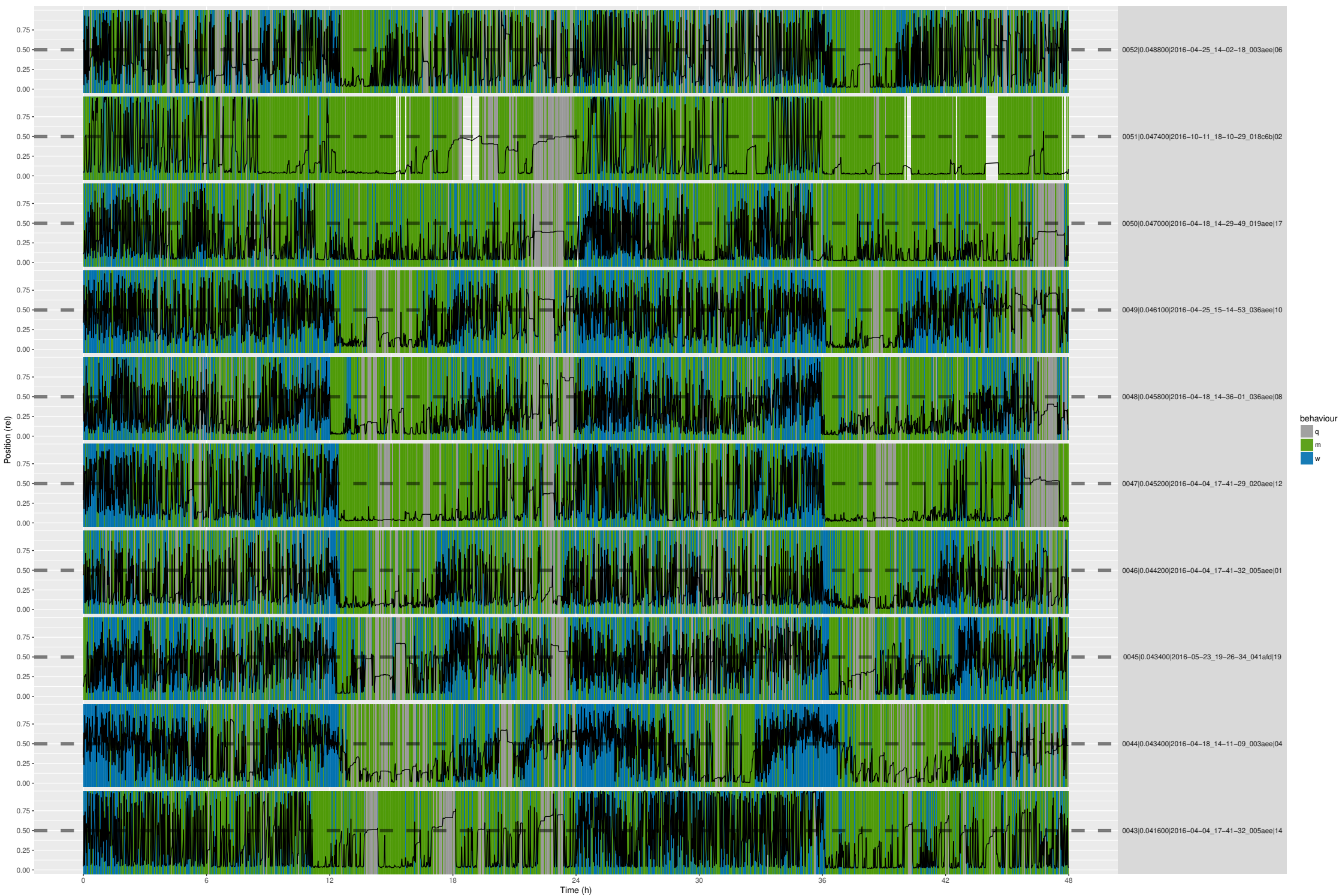

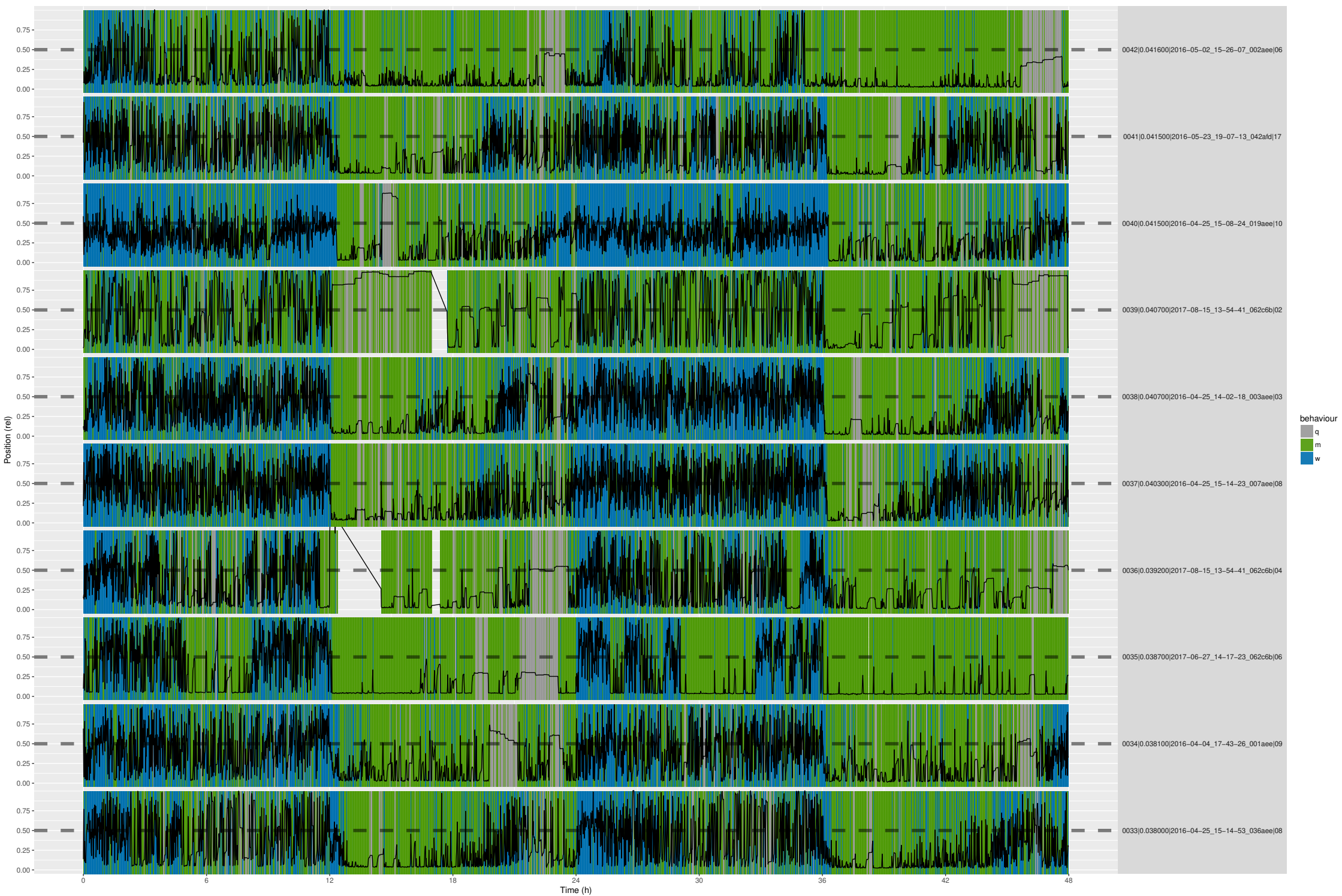

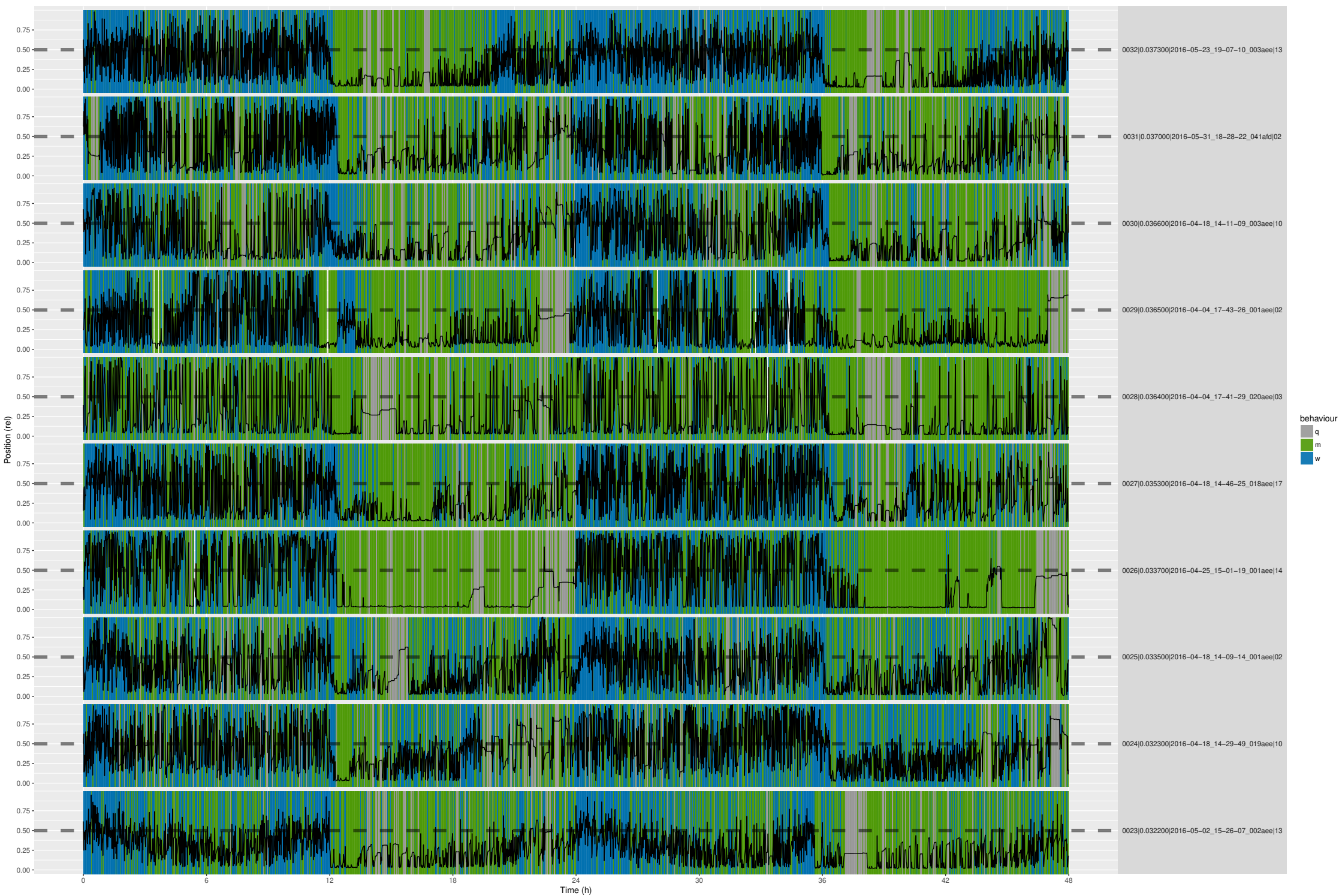

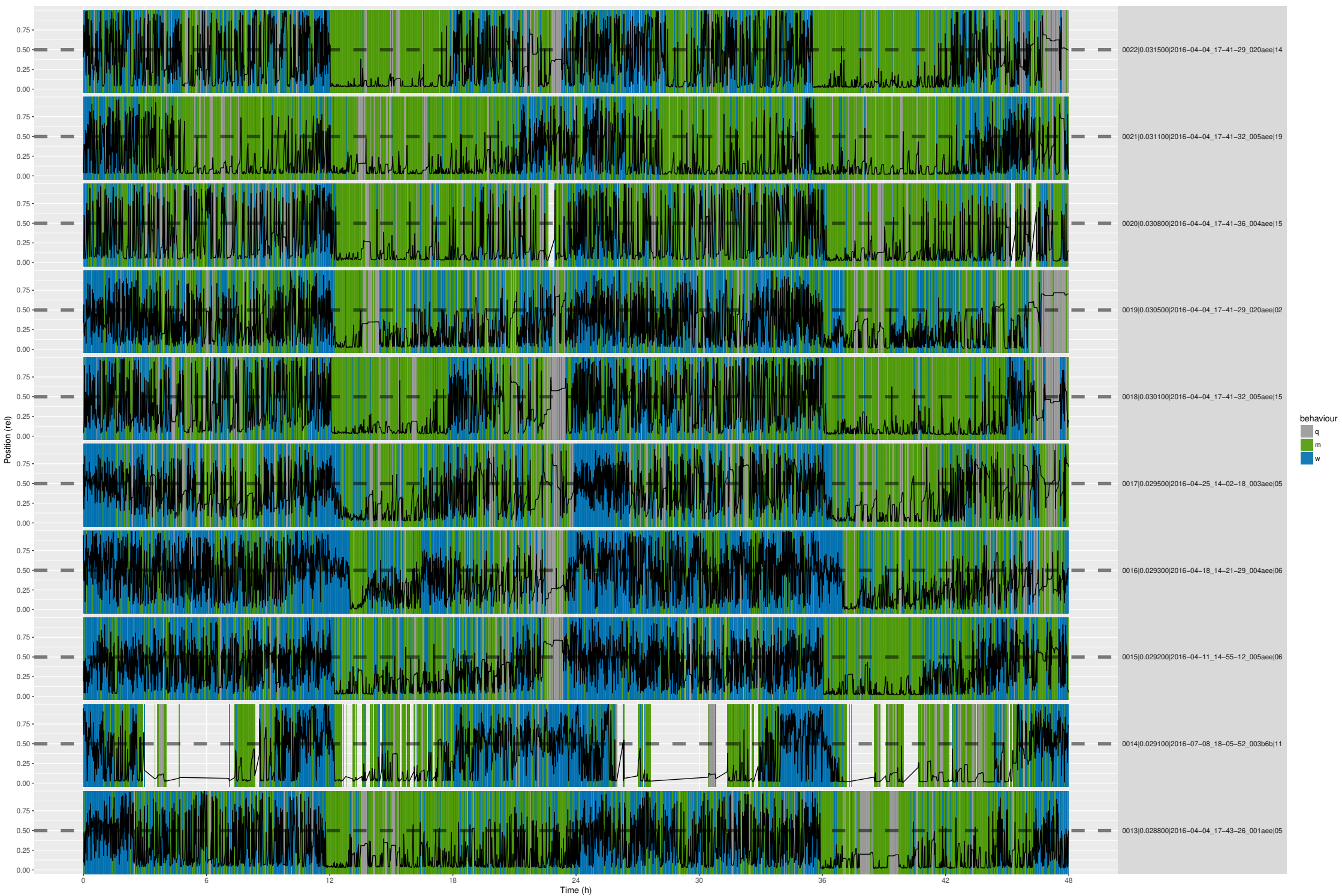

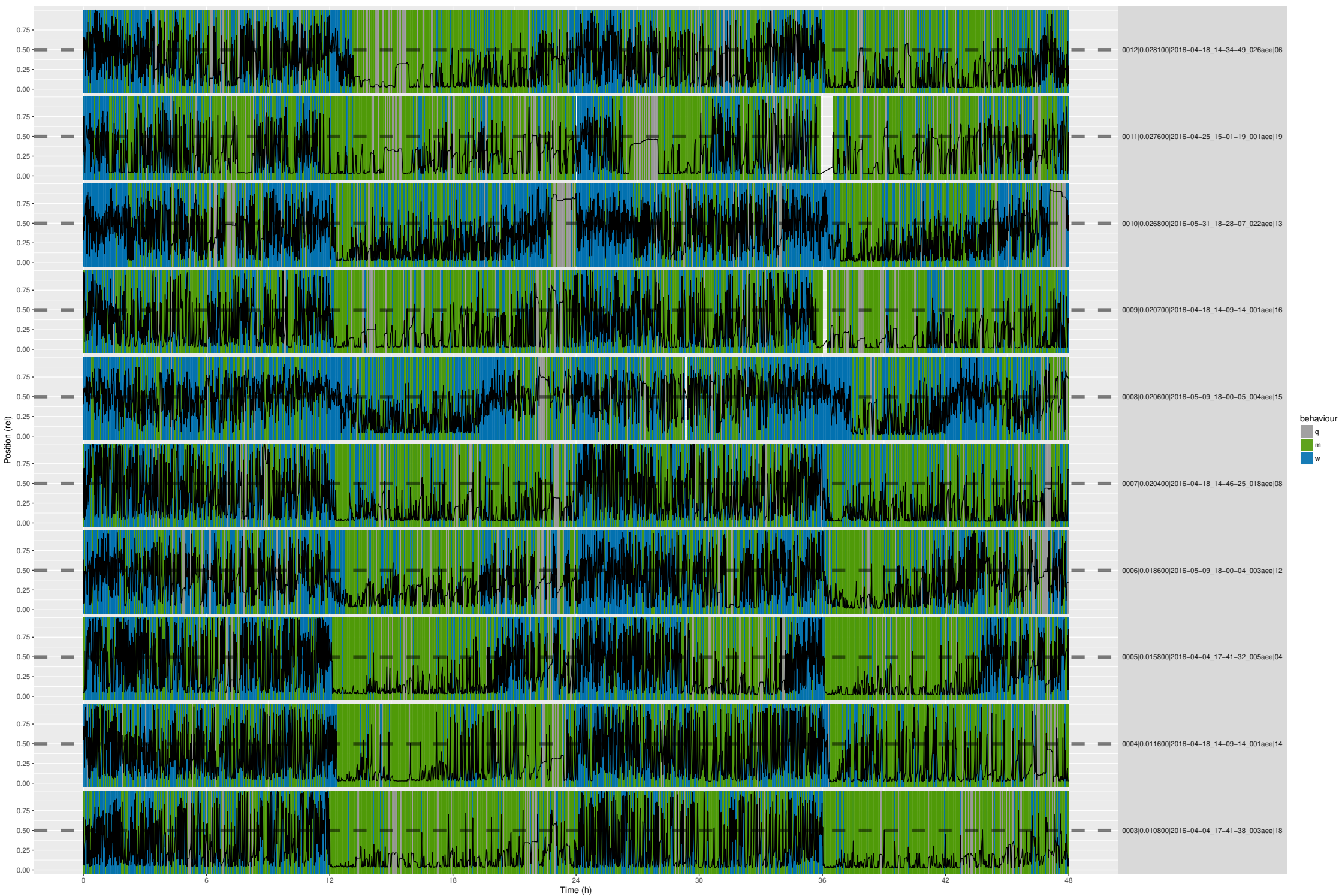

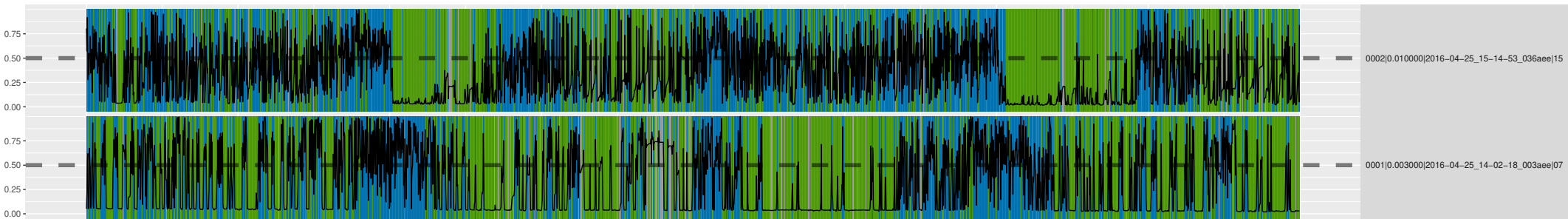

behaviour

q

m

w

Supplement: http://advances.sciencemag.org/cgi/content/full/5/2/eaau9253/DC1 [file aau9253_Figure_S1.pdf]
